# Impact of renal tubular *Cpt1a* overexpression on the kidney metabolome in the folic acid-induced fibrosis mouse model

**DOI:** 10.3389/fmolb.2023.1161036

**Published:** 2023-06-12

**Authors:** Paula Cuevas-Delgado, Verónica Miguel, Francisco J. Rupérez, Santiago Lamas, Coral Barbas

**Affiliations:** ^1^ Centre for Metabolomics and Bioanalysis (CEMBIO), School of Pharmacy, Universidad San Pablo-CEU, CEU Universities, Urbanización Montepríncipe, Madrid, Spain; ^2^ Program of Physiological and Pathological Processes, Centro de Biología Molecular “Severo Ochoa” (CBMSO, CSIC-UAM), Madrid, Spain

**Keywords:** kidney, metabolomics fingerprinting, metabolic phenotyping, mass spectrometry, mitochondria, murine model, biochemical pathways

## Abstract

**Background:** Chronic kidney disease (CKD) is characterized by the progressive and irreversible deterioration of kidney function and structure with the appearance of renal fibrosis. A significant decrease in mitochondrial metabolism, specifically a reduction in fatty acid oxidation (FAO) in tubular cells, is observed in tubulointerstitial fibrosis, whereas FAO enhancement provides protection. Untargeted metabolomics offers the potential to provide a comprehensive analysis of the renal metabolome in the context of kidney injury.

**Methodology:** Renal tissue from a carnitine palmitoyl transferase 1a (Cpt1a) overexpressing mouse model, which displays enhanced FAO in the renal tubule, subjected to folic acid nephropathy (FAN) was studied through a multiplatform untargeted metabolomics approach based on LC-MS, CE-MS and GC-MS analysis to achieve the highest coverage of the metabolome and lipidome affected by fibrosis. The expression of genes related to the biochemical routes showing significant changes was also evaluated.

**Results:** By combining different tools for signal processing, statistical analysis and feature annotation, we were able to identify variations in 194 metabolites and lipids involved in many metabolic routes: TCA cycle, polyamines, one-carbon metabolism, amino acid metabolism, purine metabolism, FAO, glycerolipids and glycerophospholipids synthesis and degradation, glycosphingolipids interconversion, and sterol metabolism. We found several metabolites strongly altered by FAN, with no reversion induced by Cpt1a overexpression (v.g. citric acid), whereas other metabolites were influenced by CPT1A-induced FAO (v.g. glycine-betaine).

**Conclusion:** It was implemented a successful multiplatform metabolomics approach for renal tissue analysis. Profound metabolic changes accompany CKD-associated fibrosis, some associated with tubular FAO failure. These results highlight the importance of addressing the crosstalk between metabolism and fibrosis when undertaking studies attempting to elucidate the mechanism of CKD progression.

## Graphical Abstract

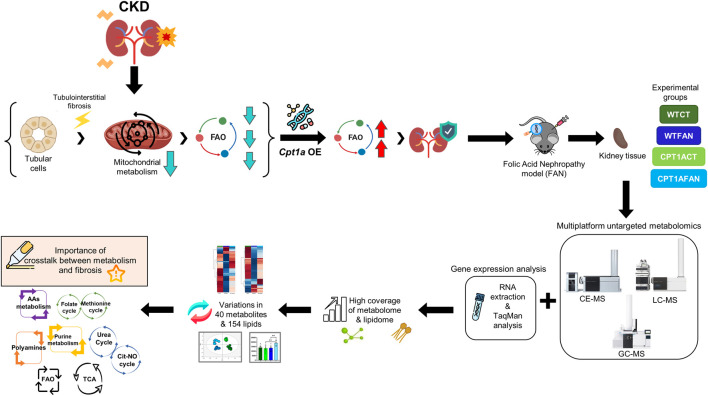

## 1 Introduction

Chronic kidney disease (CKD) is defined by the KDIGO guidelines as the presence of abnormalities in kidney structure or function for more than 3 months that affect the individual’s health, and is classified based on glomerular filtration rate (GFR) and albuminuria category (CGA) (Willis et al., 2013; AIRG-E et al., 2022). This condition is a complex disease that coexists with numerous pathologies and impacts on multiple organs, with a worldwide prevalence of more than 10% in the general population (Kovesdy, 2022). Due to its high mortality, increased incidence and lack of effective therapies, there is a huge need to develop therapeutic strategies to stop/revert its progression. Hence, it is critical to understand the molecular mechanisms of CKD progression (Ruiz-Ortega et al., 2020).

Regardless its etiology, CKD is characterized by the progressive and irreversible nephron loss, microvascular damage, a reduction of the renal regenerative capacity, oxidative stress environment, profound metabolic alterations and inflammation, which lead to tubulointerstitial fibrosis and glomerulosclerosis (Ruiz-Ortega et al., 2020). The process of fibrosis is part of the normal reparative response of the organ. However, this repair mechanism may become perpetuated in a deregulated way, promoting a cellular phenotype that produces an excess of extracellular matrix (ECM), which replaces the functional cellular components and reduces the organ function (Rayego-Mateos et al., 2021). In the last few years, there have been important advances in understanding the mechanisms leading to renal fibrosis, including new mediators involved and associated metabolic changes (Fontecha-barriuso et al., 2022).

Recently, a profound metabolic disturbance has been described as is a key contributor to the development of fibrosis (Doke and Susztak, 2022). In CKD, mitochondrial defects, particularly a reduction in β-oxidation, in renal tubules contribute to epithelial atrophy, inflammation, cell death and fibrosis development (Kang et al., 2015). In human and mouse models with tubulointerstitial fibrosis, there is a lower expression of the critical enzymes and regulators of fatty acid oxidation (FAO), resulting in energy deficit, altered lipid metabolism and lipotoxicity both in renal tissue and globally in plasma. This lends a basis for therapeutic strategies directed towards restoration or enhancement of fatty acid metabolism through a pharmacological or genetic approaches (Schlaepfer and Joshi, 2020). Thus, Miguel, V. et al. (Miguel et al., 2021) have demonstrated that the FAO gain of function (FAO-GOF) could protect from renal fibrosis in different animal models. For that, they obtained a conditional overexpression of the mitochondrial fatty acid shuttling enzyme carnitine palmitoyl-transferase 1A (CPT1A) in renal tubular epithelial cells (TECs), using a transgenic mouse model based on the doxycycline-inducible transgenic system Tet-On controlled with the transactivator (rtTA2s-M2) driven by the paired box 8 (Pax8) promoter, which significant mitigated renal fibrosis. This was related to enhanced mitochondrial mass, repaired architecture and bioenergetic recovery. This overexpression was also associated with a reduced inflammatory pattern and abrogation of TGF-β–induced epithelial cell damage (Miguel et al., 2021).

However, how the alterations in renal metabolism impact fibrosis development and CPT1A OE are not completely understood. The assessment of the renal metabolome in CKD and expected FAO-GOF by CPT1A OE may provide valuable information in understanding the complex interaction between metabolism and kidney disease. Previous studies in plasma, urine and renal tissue samples are present in the literature, but several issues hamper a clear-cut interpretation of the results. These include a broad etiological spectrum related to fibrosis and CKD, the use of different model organisms, the heterogeneity of patient cohorts and the employment of experimental animal and cell culture models (Cuevas-Delgado et al., 2022). In metabolomics, untargeted analyses allow to comprehensively measure the metabolic profile of a sample in a given physiological condition, offering the potential to provide a “snapshot” of a specific kidney state and the repercussion of CKD on the whole-body phenotype. Therefore, it is crucial to achieve the maximum possible metabolite coverage, a goal that may be accomplished by multiplatform analysis. In this study, we have used different separation techniques coupled to mass spectrometry, such as liquid chromatography, gas chromatography and capillary electrophoresis, to reveal the highest proportion of the affected renal metabolome and lipidome in a FAN murine model with CPT1A overexpression. We aim to determine the metabolic changes underlying CKD-associated fibrosis and evaluate the metabolic impact of the expected FAO-GOF in this context.

## 2 Material and methods

### 2.1 Study design

The animal model and procedures used in this study has adequately followed the Guide for the Care and Use of Laboratory Animals in Directive 2010/63/EU of the European Parliament (DIRECTIVE, 2010, 2010). All the animals and sample collection procedures were carried out at the CBMSO center under EU regulation. The conditional transgenic mouse model for renal tubular fatty acid oxidation gain-of-function (FAO-GOF) generated by Miguel V et al. (Miguel et al., 2021) was subjected to the folic acid-induced nephropathy (FAN) model. Briefly, animals were intraperitoneally injected with 250 mg folic acid per kg body weight dissolved in 0.3 M sodium bicarbonate (vehicle) as previously described (Fink et al., 1987; Miguel et al., 2021). The control animals group received only 0.3 ml of vehicle. After 15 days of folic acid administration, mice were sacrificed by CO_2_ overdose, and kidneys were harvested after perfusion with PBS (Miguel et al., 2021).

**FIGURE 1:**
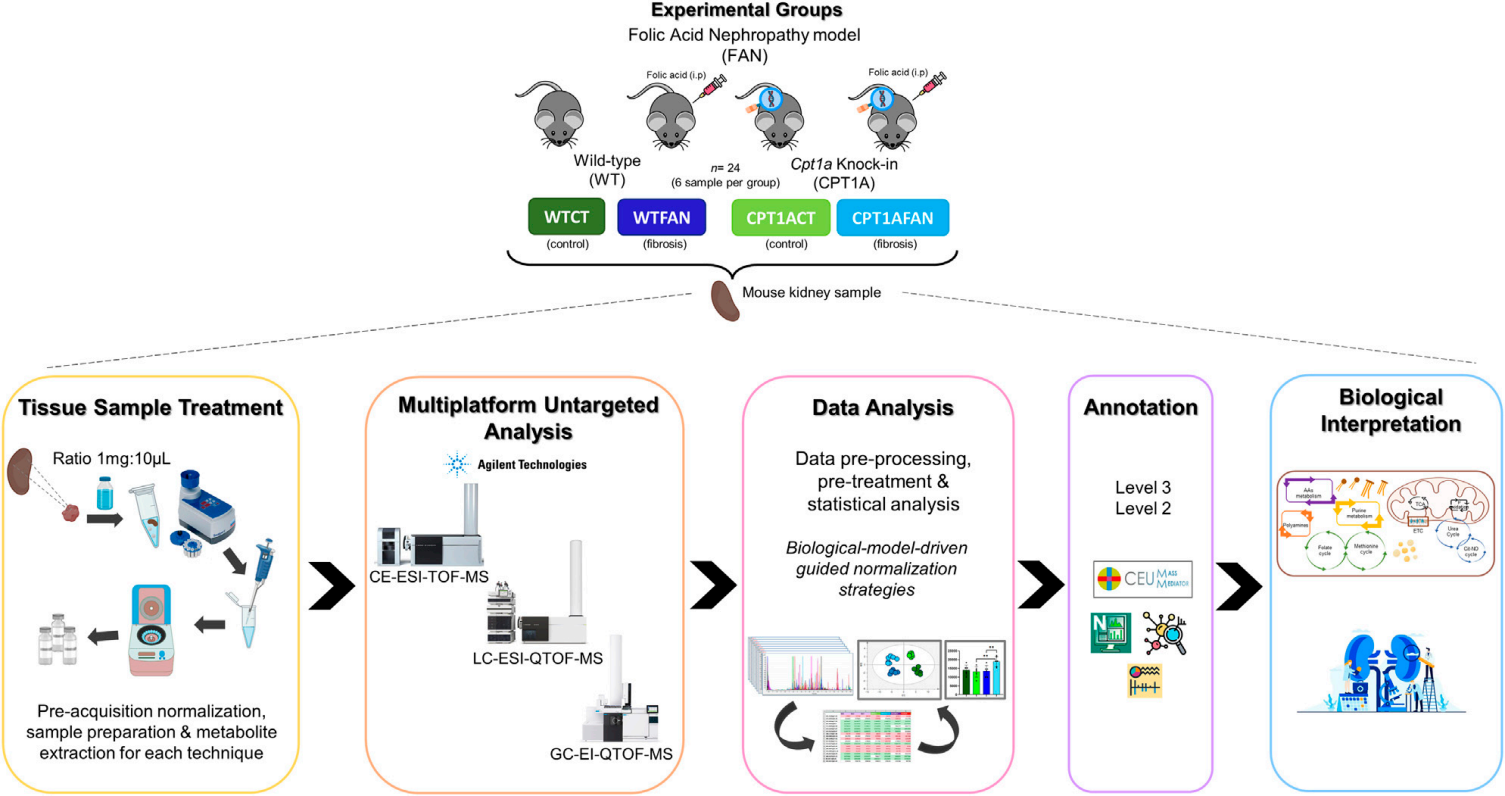
General workflow presented in this study. Experimental design used in this study, with the experimental groups, sample treatment, multiplatform metabolomics analysis, data analysis, annotation step and corresponding biological interpretation.

Kidney samples in this study were classified in four experimental groups (6 individuals in each group): wild type control (WTCT), wild type folic acid-induced nephropathy (WTFAN), *Cpt1a* overexpression control (CPT1ACT) and *Cpt1a* overexpression folic acid-induced nephropathy (CPT1AFAN) (Figure 1).

### 2.2 Multiplatform untargeted metabolomics analysis

#### 2.2.1 Chemicals

All organic solvents and chemicals were of analytical grade from Merck/Sigma-Aldrich (Germany), VWR International/BHD Prolabo (Spain) and Agilent Technologies (United States ). For more details, see Supplemental Material.

#### 2.2.2 Sample collection and processing

After kidney collection, they were dissected half lengthwise and crosswise to obtain four equal pieces of each kidney sample, which were immediately frozen in liquid nitrogen and stored at −80 °C until analysis.

Tissue disruption and homogenization were carried out by following our previous protocol (Naz et al., 2013) with some modifications, extensively explained in the Supplemental Material. Quality control samples (QCs) and blank samples were prepared for each analytical technique. Blank samples were analyzed at the beginning and at the end of the analytical sequence and QC samples were injected at the beginning of the sequence, after running 5-6 experimental samples, and at the end of the analysis sequence. All the experimental samples were randomized before the sample preparation and analytical run.

#### 2.2.3 Untargeted metabolomics fingerprinting by CE-TOF-MS

A 7100 capillary electrophoresis system (Agilent Technologies) coupled to a 6224 TOF Mass Spectrometer (Agilent Technologies), equipped with an electrospray ionization source (ESI), was used to carry out the analysis. The conditions of the analysis have been previously described (Cuevas-Delgado et al., 2020) (Supplemental Material). For general data acquisition the fragmentor was set up at a voltage of 125 V. However, at the end of the sequence, two methods, one with a fragmentor voltage of 200 V and another one with a range 125–200 V, were applied in a QC sample and in two samples of each experimental group, to obtain in-source fragmentation (ISF) pattern of metabolites (Mamani-Huanca et al., 2021).

#### 2.2.4 Untargeted metabolomics fingerprinting by UHPLC-QTOF-MS

For LC-MS metabolomics analysis, a 1,290 Infinity II UHPLC system (Agilent Technologies) coupled to a 6545-quadrupole time-of-flight (QTOF) mass spectrometer (Agilent Technologies) was used, with both ESI+ (positive) and ESI- (negative) modes to cover a broad number of metabolite ions. The conditions used for the analysis were the same as previously described (Gonzalez-Riano et al., 2021a) (Supplemental Material). At the end of the analytical sequence, using a QC sample, iterative-MS/MS runs were operated for both ion modes (Gonzalez-Riano et al., 2021b).

#### 2.2.5 Untargeted metabolomics fingerprinting by GC-QTOF-MS

GC-MS analysis was performed with an Agilent 7890B GC instrument (Agilent Technologies) coupled to a 7250 QTOF mass spectrometer system (Agilent Technologies) applying the conditions previously described (Fernández-García et al., 2020) (Supplemental Material).

#### 2.2.6 Data analysis

##### 2.2.6.1 Quality assurance procedure

Quality control and quality assurance procedures were applied according to published quality guidelines (Dudzik et al., 2017). MassHunter Qualitative analysis software (B.10.00, Agilent Technologies) was used to examine the quality of the acquired raw data (Supplementary Figure S1). The reproducibility of the different IS used in analyses was also checked at this point. After the platform-specific data pre-processing step, the resulting raw data matrix was used to perform Principal Component Analysis (PCA-X) to observe the signal drift, variation in QC samples and possible outliers, using SIMCA P+16 (Umetrics^®^, Umea, Sweden) software (Cuevas-Delgado et al., 2020). Detailed parameters and further evaluations carried out are described in Supplemental Material.

##### 2.2.6.2 GC-MS data pre-processing

In the case of GC-MS data, MassHunter Workstation GC-MS Translator (B.04.01, Agilent Technologies) and MassHunter Unknown Analysis (Tool 9.0, Agilent Technologies) were used for spectral deconvolution and metabolite identification (Gonzalez-Riano et al., 2017), more information about the followed GC-MS data pre-processing is found in Supplemental Material.

##### 2.2.6.3 LC-MS and CE-MS data pre-processing

Data acquired from LC-MS (positive and negative modes) and CE-MS analysis were pre-processed with Agilent MassHunter Profinder Software (B.08.00 and B.10.00, Agilent Technologies) following previous published steps (Gonzalez-Riano et al., 2019). For more details about LC-MS and CE-MS data pre-processing go to Supplemental Material.

##### 2.2.6.4 Data pre-treatment and statistical analysis

The four obtained data matrices (GC-MS, LC-MS+, LC-MS-, and CE-MS) were imported into Microsoft Excel (Microsoft Office 2016) for further calculations. Blank subtraction, curation of matrices, exclusion of metabolic features detected in less than 50% of QC samples, missing values imputation, and normalization step through biological model driven normalization strategies, were carried out in each of the matrices. The matrices were filtered by the coefficient of variation (CV) of QC samples, calculated as the standard deviation divided by the mean expressed as a percentage, and maintaining those features that presented a CV below 20% in LC-MS and CE-MS matrices and below 30% in GC-MS data. In the multivariate statistics analysis (MVA), unsupervised PCA-X models, and supervised partial least square-discriminant analysis (PLS-DA) method were performed using SIMCA P+16 (Umetrics^®^, Umea, Sweden) software. The values of correlation coefficient (*p(corr)*) and jackknife confidence interval were calculated, stablishing significance of those metabolites with *p(corr)* ≥ 0.5 in absolute value, and a jackknife not including 0. The software MATLAB R2018a (Mathworks, Inc., Natick, United States ) was used for the univariate statistical analysis (UVA). In this case, a Mann Whitney U test was applied to obtain significant signals (*p*-value <0.05) by comparing two groups, cases vs controls, and the false discovery rate (FDR) for multiple hypothesis testing was controlled using a standard Benjamini–Hochberg method (level α = 0.05). The percentage of change (%Change) and the logarithm in base two of the fold change (Log_2_FC) were calculated by comparing cases vs. controls for each comparison. In the case of LC-MS and CE-MS, all the statistically significant signals were tentatively annotated. More details about the performance of these steps are presented in Supplemental Material and in Supplementary Figure S2, and Supplementary Figure S3.

##### 2.2.6.5 Metabolite identification

First, for the tentative annotation (level 3) of the significant signals in LC-MS and CE-MS, the accurate m/z of the metabolic features were searched in online databases using the advanced CEU Mass Mediator (CMM) tool (Gil-De-La-Fuente et al., 2019). In the case of LC-MS data, an automated iterative MS/MS mode was used in the analysis to obtain MS/MS spectra and to obtain a putative metabolite annotation (level 2). For CE-MS data, the ion fragmentation information together with relative migration time (RMT, migration time of a feature by the migration time of the IS methionine sulfone) were compared with the CEMBIO in-house CE-MS database, available in CMM tool (Mamani-Huanca et al., 2021), to achieve the putative identification (level 2). More details about metabolite identification procedure are described in Supplemental Material. The list of metabolites annotated with different confidence levels is presented in Supplementary Table S1.

#### 2.2.7 RNA extraction and TaqMan gene expression analysis

The miRNeasy Mini Kit (Qiagen) was used to extract total RNA from mouse kidneys tissue samples. TaqMan array plates (Thermo Fisher Scientific) were selected as platform for gene expression analysis. These panels consisted of 13 unique TaqMan probes specific for mouse genes related to amino acid metabolism, lipids metabolism, nucleotides metabolism and energy metabolism (Table 1 and Supplementary Table S4). Information about the protocol followed for this analysis is presented in Supplemental Material.

**TABLE 1:** TaqMan results of gene expression of genes associated to the studied metabolic pathways in WTFAN vs. WTCT and CPT1AFAN vs. WTFAN comparisons.

		WT FAN		CPT1A FAN	
		vs. WT CT		vs. WT FAN	
Metabolic Pathaway	Gene	Fold Change	q-value	Fold Change	q-value
**TCA Cycle**	***Acly***	0.36	**0.0047**	2.24	*0.1234*
	***Cs***	0.35	**0.0013**	2.69	**0.0375**
**Urea cycle**	***Ass1***	0.21	**0.0007**	9.54	*0.0870*
**Folate and methionine cycles**	***Bhmt***	1.11	*0.2546*	0.54	**0.0069**
	***Gnmt***	0.21	**0.0002**	1.42	*0.5058*
**MAAs**	***Plod3***	0.73	*0.1998*	2.13	*0.1709*
**Polyamines**	***Odc1***	1.44	**0.0191**	2.54	**0.0179**
	***Sms***	1.26	*0.2487*	0.98	*0.9021*
	***Azin1***	0.27	**0.0160**	7.80	**0.0383**
**Lipid Metabolism**	***Fasn***	0.46	**0.0034**	1.72	*0.1799*
	***Acsm2***	0.08	*0.0729*	12.43	*0.0998*
	***Dgat2***	0.18	**0.0001**	8.53	**0.0212**
	***Srebf1***	0.90	*0.6865*	2.23	*0.1243*

MAAs: Modified amino acids; *Acly*: ATP-citrate lyase, *Cs*: citrate synthase, *Ass1*: Argininosuccinate synthetase 1, *Bhmt:* Betaine-homocysteine S-methyltransferase, *Gnmt*: glycine N-methyltransferase, *Plod3*: Procollagen-Lysine,2-Oxoglutarate 5-Dioxygenase 3, *Odc1*: Ornithine decarboxylase, *Sms*: Spermine synthase, *Azin1*: Antizyme Inhibitor 1, *Fasn*: Fatty acid synthase, *Acsm2*: Acyl-CoA, synthetase medium chain family member 2, *Dgat2:* diacylglycerol O-acyltransferase 2, *Srebf1*: sterol regulatory element-binding protein 1. mRNA, levels of these genes were determined by qRT-PCR, using TaqMan qPCR, probes in kidneys from FAN model. Statistical significance in each comparison was determined using non-parametric two-tailed Mann-Whitney test. Relative mRNA, expression was determined using 2^−ΔΔCt^, method and fold changes were normalized to values of the control group. Fold change higher than 1 indicates that the gene expression is upregulated in case group, fold change equal to 1 means no differences in the expression of the gene, while fold changes between 0 and 1 indicates a gene expression downregulation in the “case” group compared to control group. The q-values of comparisons with statistically significant results are highlighted in bold; those that are not significant are in italics. Gene abbreviations are indicated in bold italics.

## 3 Results and discussion

A multiplatform untargeted analysis, using LC-MS, GC-MS, and CE-MS analytical platforms, has been applied to evaluate the differences in the metabolomic and lipidomic profiles between fibrotic kidney tissue (FAN) and healthy renal tissues from both WT and *Cpt1a* overexpressing mice.

Considerable coverage of metabolites was attained with the multiplatform analysis: 1,157 and 1,495 features (mainly lipids) were obtained with LC-MS in positive and negative mode, respectively; 907 features with CE-MS and 214 features with GC-MS analysis. After performing signal processing and several data pre-treatment, the data matrices obtained for statistical analysis included 864 features from LC-MS (+), 670 features for LC-MS (−), 216 features from CE-MS data and 116 features from GC-MS.

In the MVA, PCA-X models for each technique were built on normalized data with suitable scaling and transforming processes. The overall quality of the data is shown in the PCA-X plots (Supplementary Figure S2) by the tight clustering of QC samples and the trends and distribution of the experimental samples.

To highlight sample grouping and to reveal the association of metabolites with the conditions, we built supervised models (Supplemental Figure S3). The *R* ^2^ and Q^2^ of the PLS-DA models (0.52 and 0.23 for LC-MS (+); 0.57 and 0.23 for LC-MS (−); 0.61 and 0.43 for GC-MS; 0.58 and 0.49 for CE-MS) indicated that the models have moderate goodness of fit but a low predictive quality. JK and p (corr) values for all compounds were obtained in these models and used for the evaluation of statistical significance of the metabolites. Moreover, the *p*-value for each comparison was obtained through UVA.

After MVA and UVAs, 40 metabolites and 154 lipids that were statistically significant were annotated (Supplementary Table S1). The comparisons and statistical values of these metabolites are represented in Supplementary Table S2 (comparisons WTFAN vs. WTCT; and CPT1AFAN vs. WTFAN) and Supplementary Table S3 (comparisons CPT1AFAN vs. CPT1ACT and CPT1ACT vs. WTCT), organized by biochemical ontologies and lipid categories.

**FIGURE 2:**
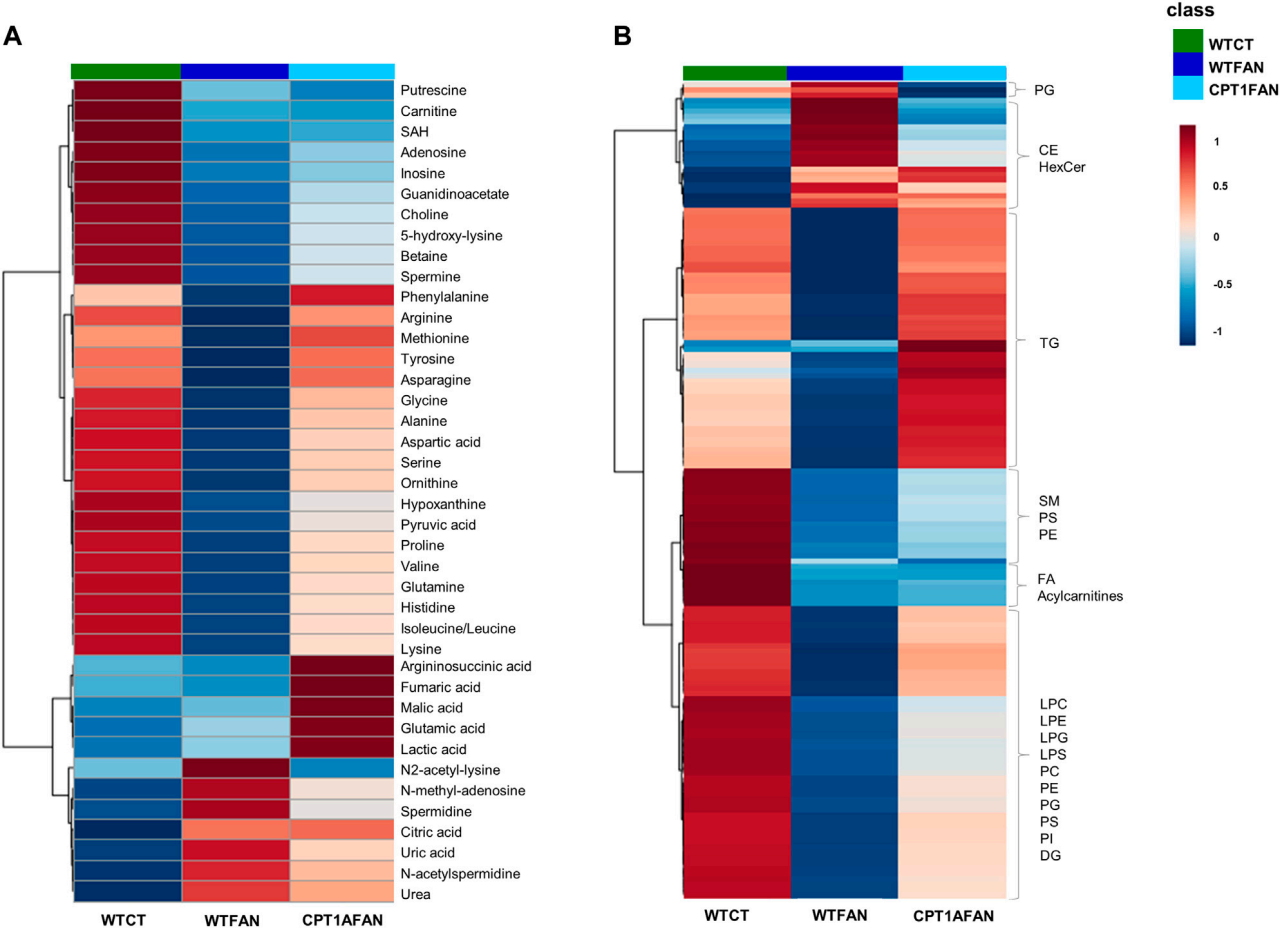
Heatmaps with group average of significantly different metabolites and lipids detected in kidney tissue from FAN model. Hierarchical clustering with Euclidean distance measure and Ward clustering. **(A)** statistically significant metabolites obtained by CE-MS and GC-MS; **(B)** statistically significant lipids obtained by LC-MS (+) and (−). These heatmaps were generated with Metaboanalyst 5.0 tool, https://www.metaboanalyst.ca (Pang et al., 2021).

The average intensities of the significant metabolites and lipids in the WTFAN, CPT1AFAN and WTCT group are depicted in two heatmaps (Figure 2).

Most of the significantly different metabolites belong to key biochemical pathways, such as the TCA cycle, urea cycle, one-carbon, polyamines and purine metabolisms. Supplementary Figure S4A shows the main metabolites with significant changes in the different comparisons. In panel B, this metabolic map is organized by pathways and cycles. Amino acids and amino acid-related compounds are the most prominent compounds in Figure 2A chart, whereas Figure 2B is comprised only of the significantly different lipids. These lipids belong to families such as fatty acyls (fatty acids, its conjugates, and fatty esters, FA), glycerophosphocholines (PC), glycerophosphoethanolamines (PE), glycerophosphoglycerols (PG), glycerophosphoserines (PS), glycerophosphoinositols (PI), diglycerides (DG), triglycerides (TG), ceramides (Cer), glycosphingolipids (HexCer), phosphosphingolipids (SM) and sterols (CE).

Different metabolic patterns can be clearly associated with fibrosis development (WTCT and WTFAN groups). Most metabolites and lipids are downregulated in the WTFAN group compared to the WTCT one, except for N2-acetyl-lysine, N-methyl-adenosine, spermidine, citric acid, uric acid, N-acetylspermidine, urea, lipids of the sterol family and the phosphoglycerols class, which are significantly upregulated in the fibrosis group.

The OE of *Cpt1a* in the presence of fibrosis induces changes in the trends of some metabolites and lipids. We can observe the upregulation of some metabolites (mainly amino acids) and some lipids (triglycerides and glycerophospholipids) in the CPT1AFAN group compared to the WTFAN one, while the metabolic profile of CPT1AFAN resembles that of the WTCT group. However, changes in sphingolipids and FA are not reverted by *Cpt1a* OE, remaining similar in both fibrosis groups (WTFAN and CPT1AFAN). It is also important to note that certain metabolites in the CPT1AFAN group present an increase in their levels compared to WTFAN, but also in comparison to WTCT. This is the case of argininosuccinic, fumaric, malic, glutamic and lactic acid. On the contrary, a downregulation in the case of PG is observed when compared to both WTFAN and WTCT groups.

A TaqMan analysis was performed to correlate the changes in metabolites with the expression of some genes coding for key enzymes involved in the metabolic pathways (Table 1; Supplementary Table S4). The most revealing gene expression results and their consistency, concerning metabolite and lipid changes will be described in the following sections.

### 3.1 Kidney fibrosis causes citric acid accumulation

Regarding the tricarboxylic acid cycle (TCA cycle), a large increase in citric acid levels is observed in the presence of fibrosis compared to control group. This increase is maintained in the group of *Cpt1a* OE with renal damage. The rest of intermediates of the cycle do not present changes in WTFAN vs. WTCT but are slightly increased in the CPT1AFAN group compared to WTFAN one. Directly related to the TCA cycle, pyruvic acid does show a significant decrease in fibrosis, while it increases in *Cpt1a* OE with fibrosis compared to the WTFAN group. Lactic acid levels show a slight increase in CPT1AFAN kidney tissue compared to the WTFAN one (Supplementary Table S2). Genes coding for the two enzymes involved in citrate production and consumption, citrate synthase (CS) and ATP-citrate lyase (ACLY), present a downregulated expression in the WTFAN group compared to WTCT one, while the OE of *Cpt1a* along with fibrosis induced an upregulation of these genes in comparison with the WTFAN group, although only statistically significant in the case of CS (Table 1).

**FIGURE 3:**
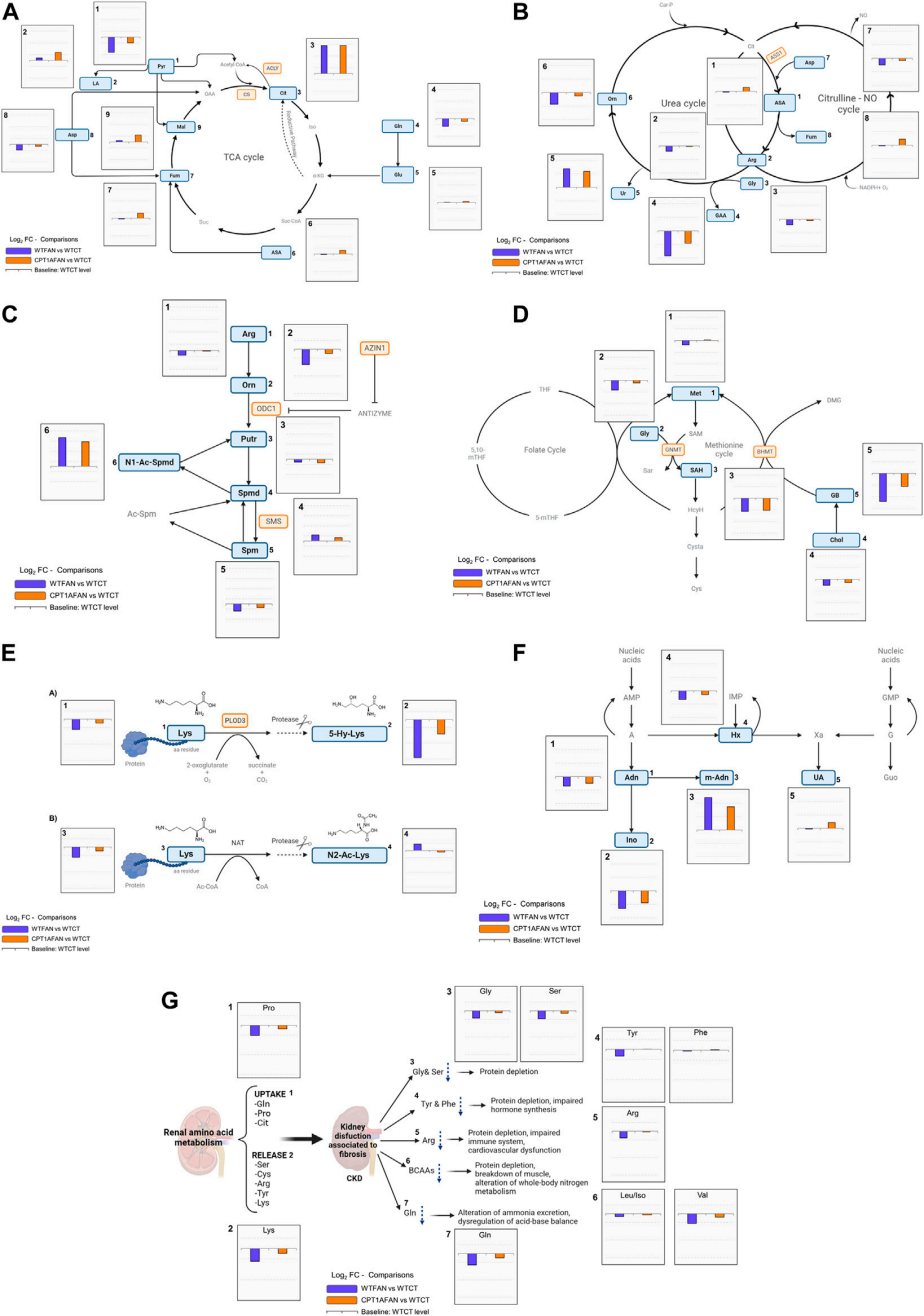
General overview of biochemical pathways highlighting changes of metabolites. Annotated metabolites indicated in Supplementary Tables S2, S3 are represented in blue rectangles. Metabolites not detected or not significant are represented in grey color. The enzyme genes analyzed highlighted in orange. WTFAN vs. WTCT (majorelle blue) and CPT1AFAN vs. WTCT (orange) comparisons are shown as Log_2_FC. **(A)** TCA cycle, bar graphs with *y*-axis scaling from 2.5 to −1.5, 1) Pyr: pyruvic acid, 2) LA: lactic acid, 3) Cit: citric acid, 4) Gln: glutamine, 5) Glu: glutamic acid, 6) ASA: argininosuccinic acid, 7) Fum: fumaric acid, 8) Asp: aspartic acid, and 9) Mal: malic acid, CS: citrate synthase, and ACLY: ATP-citrate lyase. **(B)** Urea and Citrulline-NO cycles, bar graphs with *y*-axis scaling from 2 to −2, 1) ASA: argininosuccinic acid, 2) Arg: arginine, 3) Gly: glycine, 4) GAA: guanidinoacetate, 5) Ur: urea, 6) Orn: ornithine, 7) Asp: aspartic acid, 8) Fum: fumaric acid, ASS1: argininosuccinate synthase 1. **(C)** Polyamines, bar graphs with *y*-axis scaling from 3.5 to −1.5, 1) Arg: arginine, 2) Orn: ornithine, 3) Put: putrescine, 4) Spmd: spermidine, 5) Spm: spermine, and 6)N1-Ac-Spmd: N1-acetylspermidine, ODC1: ornithine decarboxylase 1, SMS: spermine synthase, and AZIN1: antizyme inhibitor 1. **(D)** Methionine and folate cycles, bar graphs with *y*-axis scaling from 1 to −1.5) Met: methionine, 2) Gly: glycine, 3) SAH: S-adenosyl-homocysteine, 4) Chol: choline, 5) GB: glycine-betaine, BHMT: betaine-homocysteine S-methyltransferase, and GNMT: Glycine-N methyltransferase. **(E)** MAAs, bar graphs with *y*-axis scaling from 1 to −2.5, 1) Lys: lysine, 2) 5-Hy-Lys: 5-Hydroxy-lysine, 3) Lys: Lysine, and 4) N2-Ac-Lys: N2-acetyl-lysine. The enzyme gene analyzed in TaqMan analysis is highlighted in orange PLOD3: procollagen-lysine,2-oxoglutarate 5-dioxygenase 3. **(F)** Purines, bar graphs with *y*-axis scaling from 2.5 to −2, 1) And: adenosine, 2) Ino: inosine, 3) m-And: methyl-adenosine, 4) Hx: hypoxanthine, and 5) UA: uric acid. **(G)** AAs, bar graphs with *y*-axis scaling from 1 to −2.5, 1) Pro: proline, 2) Lys: lysine, 3) Gly: glycine and Ser: serine, 4) Tyr: tyrosine and Phe: phenylalanine, 5) Arg: arginine, 6) Leu/Iso: Leucine/Isoleucine and Val: valine, and 7) Gln: glutamine.

Figure 3A shows the changes observed in metabolites of the TCA cycle, representing the Log_2_FC of the WTFAN vs. WTCT and CPT1AFAN vs. WTCT comparisons, always considering as baseline the level of the metabolite in the WTCT group. With this representation, we can observe how fibrosis and overexpression of *Cpt1a* affect metabolite levels.

Citric acid shows one of the biggest differences for any comparison in this study (Figure 3A). In most of the studies performed in animal models of renal fibrosis, citric acid has been found significantly altered. Likely in our case, previous studies report an increase in citric acid in renal fibrosis (Ke et al., 2021), although, reported changes depend on the type of sample.

In our model, folic acid-induced renal fibrosis could promote citrate accumulation in renal tissue due to alterations in the fatty acid synthesis pathway. The acetyl-CoA carboxylase (ACC) enzyme produces malonyl-CoA from Acetyl-CoA, which is the building block for new fatty acids. As the ACC is the point of control mid-and long-chain fatty acid synthesis, it is subject to two types of regulation, phosphorylation and allosteric regulation (Wang et al., 2022). Allosteric regulation occurs as feedback, with inhibition by palmitoyl-CoA and activation by citrate (Brownsey et al., 2006). Regarding the phosphorylation of ACC, is controlled by the action of the AMPK enzyme. When the cell energy status is low, the AMPK enzyme is phosphorylated and activated, producing the phosphorylation of ACC, which is inactive (Brownsey et al., 2006; Wang et al., 2022). But still, the phosphorylated ACC can be partially activated by the allosteric regulation of citrate. The data present in the study of V. Miguel (Miguel et al., 2021) about the AMPK and ACC phosphorylation in the FAN model’s renal tissue show an increase in the ACC inactivated form. It is possible that the accumulation of citric acid herein detected derives from homeostatic responses directed towards the restoration of ACC activity as an essential step for fatty acid synthesis.

Additionally, in some studies of diabetes-associated CKD, the expression of genes coding for enzymes involved in the TCA cycle, are decreased in CKD (Jiménez‐uribe et al., 2021). In our data, CS is reduced in the WTFAN group. We hypothesize that this level could be a consequence of its inhibition by a reduction in expression, due to the large amount of citrate present. However, it should also be noted that this enzyme is commonly used as a marker of oxidative and respiratory capacity, indicating mitochondrial integrity and mass by measuring its activity (Locatelli et al., 2022), although, its expression levels should also be considered. Therefore, in our results, the decrease in *Cs* expression in fibrosis and its increase with *Cpt1a* OE in kidney damage could be related to mitochondrial integrity and function. In addition, the expression of the gene of ACLY in the WTFAN group is also reduced, indicating that the citrate catabolism is reduced, and contributing to increased citrate levels, as well as reducing FAS synthesis, but the *Cpt1a* OE, would attempt to restore normal citrate metabolism.

The significant decrease of pyruvic acid in the WTFAN group (Figure 3A) suggests pyruvate consumption. Pyruvate can be consumed to form different metabolites, for example, pyruvate can produce lactic acid via anaerobic metabolism. Although in the case of tubulointerstitial fibrosis, fibroblasts are reported to undergo a metabolic shift favoring aerobic glycolysis, producing lactate in the presence of oxygen (Hewitson and Smith, 2021). In our data, lactic acid only significantly increases when *Cpt1a* is overexpressed compared to WTFAN. Thus, in CPT1A OE along with fibrosis condition, aerobic and anaerobic lactate production may occur simultaneously, similar to what has been observed in studies of other nephropathy models (Ding et al., 2017).

### 3.2 Reduced expression/levels of genes/metabolite from the urea and citrulline-NO cycles associated to renal fibrosis is/are restored by *Cpt1a* overexpression

In the urea cycle, directly related to the Citrulline-NO cycle and to the production of guanidinoacetate, arginine and glycine are decreased in the WTFAN kidney tissue compared to control one. However, in the CPT1AFAN group the level of both amino acids increased compared with the WTFAN group. In addition, a clear increase in urea can be observed in the presence of fibrosis, in both WT and CPT1A kidneys, showing that OE of *Cpt1a* does not counteract it. Ornithine and guanidinoacetate show similar trends: they are significantly decreased in WTFAN (vs. WTCT) and non-significantly increased in CPT1AFAN (vs. WTFAN) (Supplementary Table S2). Related to these cycles, the expression of the gene encoding for Argininosuccinate synthetase 1 (*Ass1*) was reduced in the WTFAN group compared to the WTCT one. By contrast, *Cpt1a* overexpression led to a large increase in *Ass1* expression compared to WTFAN, although it is not statistically significant (Table 1).

ASS is responsible for the third step in the urea cycle, and the depletion of its expression has been observed in previous studies of autosomal dominant polycystic kidney disease (ADPKD) and in renal cell carcinoma (Varga et al., 2018). ASS is involved in the *de novo* synthesis of arginine in the kidney. The changes we observed in arginine and in argininosuccinic acid in our biological model are consistent (Figure 3B), indicating that renal fibrosis negatively impacts on the urea and citrulline-NO cycles and that OE of *Cpt1a* counteracts these changes.

Arginine is involved in the renal production of guanidinoacetate, a guanidino compound that is a precursor for creatine synthesis, and whose plasma, urine and tissue concentrations are affected by renal damage, because this metabolite is synthesized primarily in the kidneys. In this study, the levels of guanidinoacetate in fibrosis are significantly reduced, with opposite change after CPT1A OE (Figure 3B). In agreement, the levels of guanidinoacetate have been found to be reduced in plasma and urine after kidney nephrotoxicity or injury (Taes et al., 2008).

Thus, in general, the OE of *Cpt1a* partially restores the levels of these metabolites, suggesting that mitochondrial function is boosted by the OE of *Cpt1a*.

### 3.3 Polyamines catabolism is altered in renal fibrosis with the increase of N1-acetylspermidine

In our results, polyamines spermidine and spermine present statistically significant changes in fibrosis compared to control, although spermidine increases and spermine decreases in this group. Putrescine decreases when the *Cpt1a* is overexpressed compared to its control. The most remarkable change in the level of a metabolite within this pathway is the case of N1-acetylspermidine, which presents much higher levels in the fibrosis groups (WTFAN and CPT1AFAN) than in the control (WTCT), with no reduction associated to the *Cpt1a* OE (Supplementary Table S2; Supplementary Table S3; Figure 3C).

The expression of genes that codify for ornithine decarboxylase (ODC1), the rate-limiting enzyme of polyamine biosynthesis catalyzing ornithine to putrescine conversion; spermine synthase (SMS), which catalyzes the production of spermine from spermidine; and antizyme inhibitor 1 (AZIN1), a protein that regulates ODC; are shown in Table 1. *Odc1* displays higher expression in fibrosis-related groups, being more expressed in the CPT1AFAN group than in WTFAN one. In contrast, *Azin1*, which inhibits the antizyme protein, which is an inhibitor of ODC1, is reduced in WTFAN compared to WTCT. However, OE of CPT1A promoted a 7-fold increase in its levels in the context of fibrosis (CPT1AFAN vs. WTFAN). Finally, *Sms* presents an increased expression only in the CPT1AFAN group compared with the CPT1ACT.

In this regard, the OE of *Cpt1a* under fibrotic conditions enhanced the expression of *Odc1* and *Azin1*, indicating a higher activity of ODC and synthesis of polyamines, which could be related to a reduction in the TGF-β/Smad3 fibrosis response. MiR-433 has been identified as an important component of this pathway and Azin1 was identified target. OE of miR-433 suppresses Azin1 expression, while, in turn, Azin1 upregulation could suppress the TGF-β/Smad3 signaling and the fibrotic response (Li et al., 2013). Our data support the importance of this pathway in the development of renal fibrosis.

Polyamines are catabolized through their back-conversion via the spermidine/spermine N1-acetyltransferase/N1-acetylpolyamine oxidase cascade or oxidized by spermine oxidase. It can generate toxic intermediates, such as H_2_O_2_ and aminoaldehydes, which disrupt the integrity of lysosomal and mitochondrial membranes, inducing endoplasmic reticulum stress response (ERSR) (Zahedi et al., 2017).

Moreover, studies of renal damage with spermine supplementation support kidney damage mitigation, suggesting that spermine may exert a protective role in the progression of damage by promoting autophagy, inhibiting inflammatory reactions, oxidative stress and ERSR (Zhang et al., 2021).

### 3.4 One-carbon metabolism: folate and methionine cycle alterations in renal fibrosis

Few changes were observed in metabolites belonging to the folate and methionine cycles (Supplementary Table S2; Figure 3D). *N,N,N-*trimethyl-glycine (glycine-betaine) was largely decreased in the WT fibrosis group compared to the control one. OE of *Cpt1a* increased its levels, but not significantly. S-adenosylhomocysteine (SAH) was significantly decreased in fibrosis condition compared to control. Also, a non-significant trend of decrease is observed in choline and methionine levels in fibrosis compared to control. However, *Cpt1a* OE only restored methionine and choline levels without affecting the levels of the other metabolites (Supplementary Table S3).

In this pathway, the genes analyzed were related to the metabolism of the methylated derivatives glycine, such as betaine-homocysteine S-methyltransferase (*Bhmt*) and glycine N-methyltransferase (*Gnmt*) (Table 1; Supplementary Table S4). *Bhmt* expression was not different between the WTFAN and WTCT groups, but the OE of *Cpt1a* decreased the expression of *Bhmt*, affecting both CPT1AFAN and CPT1ACT groups. This enzyme catalyzes the transfer of a methyl group from glycine-betaine to produce DMG. Assuming a correlation between expression and function, this would explain the observed accumulation of glycine-betaine. In the case of *Gnmt*, which is an enzyme involved in transmethylation, the expression was reduced in both fibrosis groups but not affected by the OE of *Cpt1a*. In this way, gene expression analysis of *Bhmt* and *Gnmt* yields changes that are consistent with those of glycine-betaine. It seems that the OE of *Cpt1a* partially restores the basal methylation process.

The literature highlights the importance of one-carbon metabolism in kidney damage. Different studies have indicated that changes in metabolites such as glycine-betaine, SAM, SAH, homocysteine, methionine and cysteine are associated with different cellular mechanisms and could be used as biomarkers or therapeutic targets. For instance, glycine-betaine is well known for its role as osmoprotectant and methyl donor. However, accumulating evidence has shown that glycine-betaine also has anti-inflammatory functions. Mechanisms involve the counteraction of sulfur amino acid metabolism induced oxidative stress, via stimulation of methionine production; inhibition of the transcription nuclear factor KB, inhibition of the NLRP3 inflammasome; and activation and mitigation of ER stress and apoptosis, by avoiding misfolded proteins and downregulating the transcription factor ATF-3 (Zhao et al., 2018).

### 3.5 A general decrease in amino acid levels associated to CKD-fibrosis is thwarted by *Cpt1a* OE

As a general pattern, fibrosis induced by folic acid involves a decrease in the abundance of the amino acids (AAs). In most cases, OE of *Cpt1a* prevents this downregulation reflected in a slight increase of AAs in CPT1AFAN group compared to WTFAN group. For instance, alanine, proline, valine, histidine, tyrosine, lysine, serine and aspartic acid follow this trend. Nevertheless, there was no significant decrease in isoleucine/leucine, phenylalanine, asparagine, and glutamic acid levels in WTFAN vs. WTCT, with no further modification by *Cpt1a* OE, and comparable levels between WTFAN and CPT1AFAN (Figure 3G; Supplementary Table S2).

Kidney plays an important role in the synthesis and interorgan exchange of numerous amino acids (Li et al., 2020). Therefore, alterations in their levels associated to fibrosis may affect homeostasis in the whole organism (Li et al., 2020).

Renal amino acid metabolism plays a key role in acid-base balance regulation via glutamine hydrolysis and ammonia excretion, and in whole-body nitrogen metabolism by the regulation of branched-chain amino acids (BCAA) pools. Some catabolic factors, such as inflammation, are responsible for the alteration of the renal acid-base balance and BCAA metabolism (Li et al., 2020). In our database glutamine and valine are decreased in the fibrosis group compared to its control (Figure 3G). Moreover, FAN also affects the conversion of phenylalanine into tyrosine. The postabsorptive state of the kidney plays a major role in the uptake of phenylalanine, its hydroxylation and release of tyrosine (Kopple, 2007). Thus, alterations in aromatic amino acids levels in kidney disease may result in phenylalanine overloading and tyrosine deficiency with subsequent generalized consequences in the whole organism. This imbalance is reflected in a reduction of tyrosine levels in plasma and many tissues, with either no changes or increase in phenylalanine levels (Kopple, 2007; Li et al., 2020). Our observations are consistent with this scenario (Figure 3G; Supplementary Table S3).

Overall, it is noteworthy that with the OE of the *Cpt1a* gene there is a restoration of all annotated amino acids. Such global impact points out the functions of CPT1A which go beyond its canonical function as acylcarnitine transferase, which could be related with the fact that *Cpt1* knockdowns had altered mitochondrial morphology and impaired mitochondrial coupling, whereas cells in which CPT1 had been pharmacologically inhibited did not (Yao et al., 2018). Therefore, *Cpt1a* OE would maintain mitochondrial integrity, preserving mitochondrial amino acid catabolism and synthesis (Miguel et al., 2021).

### 3.6 Alterations in hydroxylated and acetylated lysine levels are associated to the renal fibrosis

Two MAAs of lysine, namely, 5-hydroxy-lysine and N2-acetyl-lysine were impacted by fibrosis and *Cpt1a* OE (Supplementary Table S2; Supplementary Table S3). The hydroxylated AA presents a remarkable decrease in the WTFAN group compared to the WTCT one, while their levels in the CPT1AFAN group were higher than in THE WTFAN one (Figure 3E panel A). These changes showed the same tendency as the expression of the *Plod3* which codes for procollagen-lysine,2-oxoglutarate 5-dioxygenase 3, enzyme that mediates the hydroxylation in collagen lysine residues (Table 1). Although the changes were not statistically significant, a decrease in the WTFAN group compared to control was observed, while OE of *Cpt1a* during fibrosis was accompanied by a 2-fold increase.

5-hydroxylysine is present uniquely in collagen-like peptides whose free forms arise through proteolytic degradation (Yamauchi and Sricholpech, 2012). It is produced by the action of three different isoforms PLOD (Yamauchi and Sricholpech, 2012). Collagen is one of the main components of the ECM, which plays an important role in the pathogenesis and progression of chronic fibrosis diseases. These diseases involve changes in collagen composition, modification, crosslinking, and turnover. Consequently, its modifications accumulate in fibrotic states (Mavrogeorgis et al., 2022). As there are no significant changes in the expression of *Plod3* in our data, alteration in this MAA level may be associated with the decrease in the turnover and degradation of collagen due to the promotion of collagen deposition over degradation in fibrosis.

The level of N2-acetyl-lysine presents no significant changes in WTFAN vs. WTCT. However, when the *Cpt1a* is overexpressed in fibrosis, its levels decrease compared to the WTFAN group (Supplementary Table S2) and a large increase is observed in the CPT1AFAN group compared to CPT1ACT one (Supplementary Table S3). N2-acetyl-lysine residue derives from the degradation of proteins that have been post-translationally modified (PTM) on their lysine residues by targeted or untargeted acetylation. As this PTM impacts protein function, an equilibrated balance between acetylation and deacetylation is required. Recently, aberrant lysine acetylation has been associated with CKD, metabolic syndrome and other diseases (Gessner et al., 2019). In our data (Figure 3E panel B), levels of N2-acetyl-lysine decrease with OE of *Cpt1a* and achieve an abundance near similar values to the WTCT group. The modification of some gene variants coding for N-acetyltransferases (NAT) in the kidney has recently been associated with different concentrations of N-acetyl amino acids in plasma, which, correlates with renal failure (Luo et al., 2021). Whether the increase and reduction are due to dysfunction of NATs or variations in the availability of Acetyl-CoA is a matter for future research.

### 3.7 N-methyladenosine production is exacerbated in renal fibrosis

Some significant differences were found regarding the purine pathway. Inosine, adenosine and hypoxanthine were decreased in the WTFAN group in comparison with the WTCT one. Remarkably, N-methyladenosine was largely increased in the WTFAN group compared to its control, as well as in the CPT1AFAN group compared to the CPT1ACT one (Supplementary Table S2; Supplementary Table S3).

In our data, fibrosis was accompanied by a decrease in adenosine, inosine, and hypoxanthine (Figure 3F), with no differences associated to *Cpt1a* OE. The activity of xanthine oxidase (XO), the enzyme that catalyzes the oxidation of hypoxanthine to xanthine and to uric acid, is altered in kidney disease conditions (Terawaki et al., 2018). This catabolic enzyme is also a physiological source of superoxide ions, hydrogen peroxide and NO, which can function as second messengers and are related to redox signaling and oxidative stress (ROS) (Terawaki et al., 2018).

Furthermore, in our experimental model, an increase in methyl-adenosine in fibrosis groups was also found (Figure 3F). Several studies have reported that alterations in the levels of methylated metabolites and in the expression or activity of the enzymes that generate or degrade them are associated with various pathologies, including kidney cancer, ADPKD, acute kidney injury or obstructive renal nephropathy (Lu et al., 2021). This methylated purine base produces epigenetic modifications in DNA and RNA nucleotides, playing critical roles in the evolution of renal diseases (Shima and Igarashi, 2020).

### 3.8 Fibrosis and *Cpt1a* OE have a profound lipidomic impact

**FIGURE 4:**
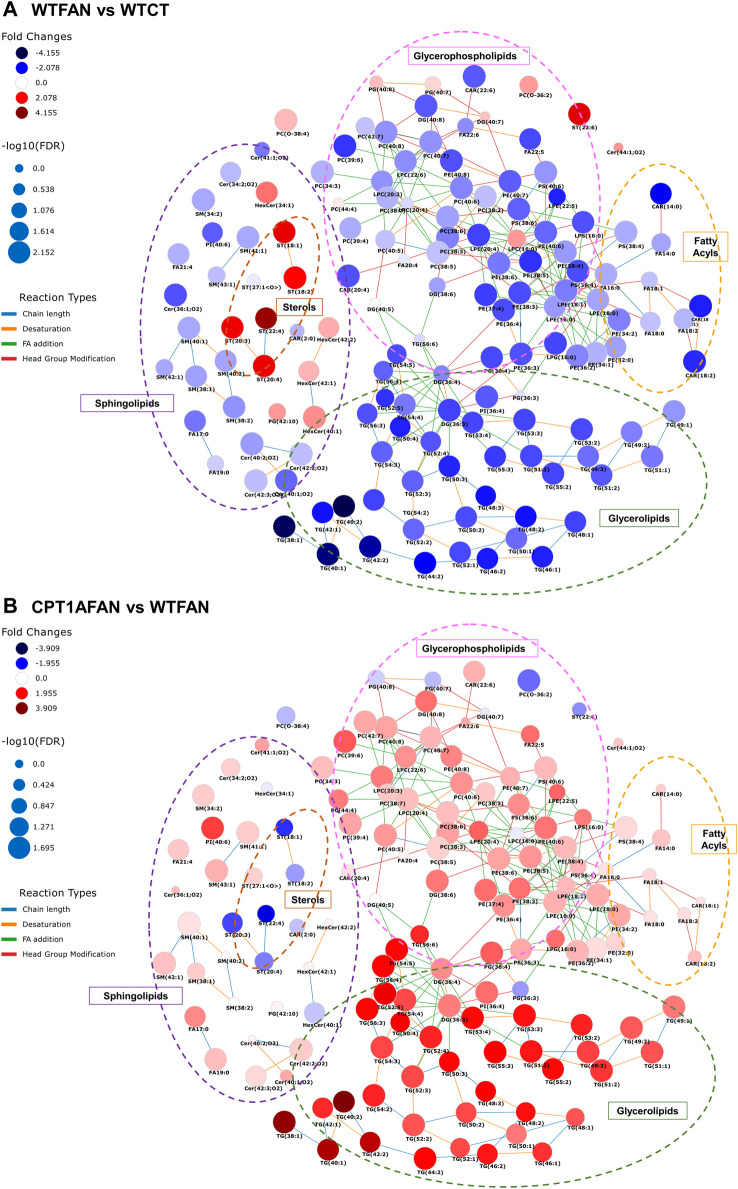
Scheme of changes in lipid metabolism in the FAN model with and without *Cpt1a* overexpression. Shown are interactive networks for WTFAN vs. WTCT **(A)** and CPT1AFAN vs. WTFAN **(B)** comparisons representing the changes in the different lipid classes. Node size is scaled by negative log_10_ of *p*-values corrected by Benjamini–Hochberg (FDR). Fold changes of lipids are represented as follows: blue colors indicate lower levels of lipids in the case condition (first group indicated in the comparison) and red colors indicate higher levels of lipids in case samples (first group in the comparison). Connectors of reaction types among lipids are colored as indicated. These networks were obtained using the LINEX web-tool (Köhler et al., 2021) (LINEX - LipidNetworkExplorer (https://exbio.wzw.tum.de/linex/)).

Both fibrosis and *Cpt1a* OE under fibrotic conditions have an impact on different lipid families in renal tissue, which are depicted in Figure 4. Panel A shows that in the WTFAN group, the levels of most molecules belonging to the different classes of lipids are decreased. Specifically, glycerophospholipids, fatty acyls, glycerolipids and sphingolipids are decreased in the fibrosis condition (WTFAN) when compared to its control. By contrast, there is an increase in sterols and hexosylceramides lipids. Of note, OE of *Cpt1a* induces quite opposite changes in lipid levels (panel B) reflected in a large increase in glycerolipids, a general increase in glycerophospholipids, sphingolipids, a slight increase in fatty acyls and a decrease in sterols and hexosylceramides. These variations support that the OE of the *Cpt1a* in the presence of fibrosis would counteract the changes in the lipid profile associated to renal damage, promoting a restorative response in the homeostasis of lipid metabolism.

Understanding how these lipids are dysregulated in CKD is challenging owing to the broad diversity of lipid structures, alteration in both synthesis and catabolism, and the different direction of changes according to the organism compartment (Baek et al., 2022). Previous studies have shown that plasma levels of fatty acids, acylcarnitines, triglycerides and ceramides are increased in patients with different CKD stages (Baek et al., 2022), while cholesteryl ester levels are decreased (Marczak et al., 2021). In our data, the changes obtained are opposite to the alterations seen in plasma, with a general decrease in most of the classes except for cholesterol esters which are increased. These results would highlight the complexity of the association of changes in the lipid profile with CKD, considering that the alterations in the kidney and plasma levels do not necessarily coincide (Sas et al., 2018).

### 3.9 FAN-induced alterations in carnitine, acyl-carnitines and fatty acid levels are mildly attenuated by *Cpt1a* overexpression

Carnitine and all acyl-carnitines detected were significantly decreased in the WTFAN group compared to WTCT. Comparing results between both fibrosis conditions, the acylcarnitine levels were slightly increased, but not significantly. In addition, comparing CPT1AFAN vs. CPT1ACT groups, differences were not statistically significant (Supplementary Table S2; Supplementary Table S3). The global trend supports that overexpression of *Cpt1a* would lead to increased levels of acylcarnitines. Furthermore, fatty acids show a decrease in their levels in the WTFAN group with respect to their control. Also, we could observe a slightly trend of augment in all fatty acids in the CPT1AFAN group compared with the WTFAN one, although without statistically significant changes. Moreover, there is a significant decrease of the two fatty esters annotated when comparing WTFAN to its control, which are significantly increased in CPT1AFAN compared with WT fibrosis (Supplementary Table S2).

We evaluated the expression of the fatty acid synthase gene (*Fasn*), the mitochondrial acyl-CoA synthetase gene (*Acsm2*), and the sterol regulatory element binding transcription factor 1 gene (*Srebf1*) (Table 1), genes that code for essential enzymes in the synthesis and degradation of fatty acids. Both *Fasn* and *Acsm2* were decreased in the WTFAN group compared to its control, although the change is only significant in the case of *Fasn*. However, with the OE of *Cpt1a*, only a remarkable 12-fold increase in the *Acsm2* expression was observed in CPT1AFAN vs. WTFAN comparison, although not reaching statistical significance. In addition, there was a non-significant slight increase in the expression levels of *Srebf1* in this comparison. The decreased levels in the expression of *Fasn*, which codes for the enzyme that catalyzes fatty acid synthesis, and *Acsm*2, involved in the first step of fatty acid metabolism through the activation of fatty acids by CoA, would most likely explain the downregulation in both the synthesis and activation of fatty acids, reflected in the decrease of fatty acids and acylcarnitines levels in the WTFAN group (Figure 4).

These data are concordant with the results described for the TCA cycle. In this way, the decreased expression of the *Acsm2* gene in the renal fibrosis group, as well as the decreasing trend observed in acylcarnitines and fatty acids, would indicate a deficiency in the pathways that catabolize fatty acids, as previously documented in kidney damage (Watanabe et al., 2020). However, overexpression of *Cpt1a* would lead to FAO-GOF through increased gene expression of *Acsm2*, which would promote β-oxidation of fatty acids in synergy with the increased presence of CPT1A itself. In addition, the slight trends of increased expression of *Fasn* and *Srebf1*, a transcription factor that controls fatty acid synthesis, would indicate that there is also an increase in the synthesis of these lipids, whose catalysis would serve for energy production of tor the synthesis of other complex lipids.

### 3.10 Glycerophospholipid family members undergo extensive changes induced by tubulointerstitial fibrosis

Different types of glycerophospholipids, *i.e.*, glycerophosphocholines, glycerophosphoethanolamines, glycerophosphoglycerols, glycerophosphoserines and glycerophosphoinositols were affected by FAN (Supplementary Table S2; Supplementary Table S3).

Within the glycerophosphatidylcholines class, there was a significant decrease in the monoacylglycerophosphocholines, *i.e.*, LPC in the WTFAN group compared with its control. This situation was reversed with *Cpt1a* OE, showing a significant increase of these lipids in the CPT1AFAN group compared with WTFAN one. The diacylglycerophosphocholines, such as PC, follow the same pattern of changes as LPC. Of note, plasmalogens from LPC and PC followed a different trend than the other glycerophosphocholines in all comparisons. In the WTFAN group, plasmalogens are increased.

The LPE, PE, LPS, PS and PI followed the same pattern as the glycerophosphocholine class in the four comparisons.

In this study, changes in the levels of LPG and PG change oppositely to the rest of glycerophospholipids. A significant increase in the levels of these lipids was found in the WTFAN group compared to their control, while a decreased trend in the OE of *Cpt1a* during fibrosis was observed compared to the WTFAN group.

Alterations in phospholipid levels in relation to kidney damage, renal fibrosis and CKD have been previously described (Zhao et al., 2015). However, the results vary according to the biological model used, the stage of the disease and the nature of the biological sample (Chen et al., 2017). Most of the studies have been performed in plasma or serum, assessing the overall increase or decrease of different classes of phospholipids within a specific kidney pathological context. Nevertheless, it should be kept in mind that changes in plasma phospholipid levels do not necessarily mirror those in urine or kidney (Sas et al., 2018). This fact precludes a clear-cut interpretation of these data and hence, a full understanding of the causes or consequences of the changes in the phospholipid metabolism related to kidney disease and/or fibrosis progression.

Our data from the FAN model show a general decrease in glycerophospholipid levels in the kidney under fibrosis (Figure 4). It is possible that these changes observed in kidney tissue reflect the increase of phospholipids in plasma and urine as renal fibrosis causes damage and disruption of kidney cell membranes, resulting in the release of their constituent phospholipids, which are eliminated into the urine or plasma to prevent their free accumulation in the tissue (Harzandi et al., 2021). Noteworthy changes in the opposite direction were detected in our study regarding PG, LPG and plasmalogens of PC. PG and LPG are both cardiolipins-synthesis intermediates and degradation products. Cardiolipins are the characteristic lipids of mitochondria, and their variations could be related to the integrity of the organelle, hence becoming altered in fibrosis and restored towards physiological levels when CPT1A is overexpressed (Yao et al., 2018).

### 3.11 Lipotoxicity vs. lipid-overload: role of FAO-GOF in the modulation of triglyceride levels in renal fibrotic tissue

The FAN model produced alterations in the glycerolipids category, more precisely in diacylglycerols (DG) and triacyclglycerols (TG) levels (Supplementary Table S2; Supplementary Table S3). DG and TG significantly decreased in the WTFAN group. By contrast, the *Cpt1a* OE under fibrosis conditions resulted in increased DG and TG, but mainly in TG level. Comparing the CPT1AFAN and WTFAN groups, TG levels showed a 2- to 31-fold increase in the CPT1FAN group.

Several enzymes are involved in the process of DG and TG synthesis. Among these are the previously mentioned ACSM2 and the diglyceride acyltransferase (DGAT), which catalyzes the last key step in TG synthesis by using DG and fatty acyl CoA substrates. In our analysis, the expression of *Dgat2* gene is decreased in the WTFAN group compared to its control, but the OE of *Cpt1a* in fibrosis condition remarkably increased its expression (Table 1).

The classical theory of renal lipotoxicity advocates that intrarenal lipid accumulation can affect the structure and function of kidney cells, causing the progression and development of renal damage (Jang et al., 2020). However, numerous studies have shown that different biological responses (protective or deleterious) can be triggered depending on the lipid species accumulated (Stadler et al., 2015; Noels et al., 2021). Therefore lipid deposition is not lipotoxic by itself, and as a consequence the concept of lipid-overload has been coined to describe the accumulation of non-toxic esterified lipids in lipid droplets (Stadler et al., 2015).

Our results on FA, DG and TG levels in this FAN model could explain the recovery produced by the *Cpt1a* OE in fibrosis in the context of lipid-overload (Figure 4). Fibrosis triggers a series of changes in renal metabolism that leads to the degradation and consumption of complex lipids such as TG and prevents their synthesis consuming FFA for other purposes. When *Cpt1a* OE counteracts fibrosis and the β-oxidation pathway is restored, TG levels increase dramatically, compared to both WTFAN and control group. Such lipid accumulation together with ongoing β-oxidation capacity does not necessarily cause renal damage (Stadler et al., 2015; Noels et al., 2021) and may be interpreted as an adaptive response to lower the concentration of lipotoxic intermediates and to control the amount of energy supply without jeopardizing the cellular and mitochondrial redox state (Engin, 2017).

In addition, results obtained in the TaqMan analysis for enzymes involved in TG synthesis and degradation follow the same pattern as that of the metabolomic analysis. *Fasn*, *Acsm2* and *Dgat2* are decreased in the WTFAN group, and upon *Cpt1a* OE there is an increase in their expression, reaching significance for *Dgat2*. Hence, this may partially explain the observed increase in TG levels in the kidneys of the CPT1AFAN group. It should be kept in mind that cellular TG levels presumably reflect a complex interplay between substrate availability, DGAT-mediated TG assembly and catabolism. Therefore, the concept that TG accumulation represents a flux of excess FFAs into TG stores is probably an oversimplification (Johnson et al., 2005).

### 3.12 Renal fibrosis produces a general decrease in sphingolipids except for the glycosphingolipids which are increased in damaged kidney tissue

Kidney fibrosis induced alterations in different classes of sphingolipids, such as ceramides, glycosphingolipids and phosphosphingolipids (Supplementary Table S2; Supplementary Table S3). Ceramides (Cer) levels were significantly decreased in the WTFAN vs. WTCT comparison. Furthermore, this decrease is also observed in sphingomyelins (SM). By contrast, in the case of glycosphingolipids, we observed a significant increase in the hexosylceramides (HexCer) levels. When *Cpt1a* is overexpressed, only a slight increase in Cer and SM was observed in CPT1AFAN compared to WTFAN group (Figure 4).

Sphingolipids are essential lipids that regulate critical cellular functions (Tanaka et al., 2022). In particular, Cer are intermediates in the formation of multiple types of sphingolipids and are structural components of the cell membrane. However, they are also critical bioactive lipids involved in a wide variety of cellular processes, such as the cellular response to stress by promoting apoptosis and inflammation (Sas et al., 2015). In several kidney disease models, the level of the Cer and HexCer is increased, most likely due to their involvement in the progression of kidney injury (Sas et al., 2015; Tanaka et al., 2022). Hexosylceramides are particularly abundant in the kidney, where they play critical roles in metabolism. In APKD studies, it has been demonstrated that inhibiting the accumulation of glycosphingolipids, prevents the progression of the disease (Natoli et al., 2010).

In our data, we observed that the accumulation of HexCer is likely related to the progression of fibrosis and renal damage. The decrease detected in Cer and SM could be due to their increased utilization in situations of advanced fibrosis or advanced stages of CKD, leading to the generation of hexosylceramides. Although alterations in sphingolipid levels are present in kidney disease, there is a general absence of correlation between sphingolipid levels in plasma and renal tissue. While plasma sphingolipid levels may primarily reflect their hepatic metabolism, especially in the case of Cer (Sas et al., 2015), their pathogenic role in kidney disease is still unclear.

### 3.13 Homogenous increase in sterol ester lipids in tubulointerstitial fibrosis

Analysis of the cholesteryl ester class (CE) revealed that, in the presence of fibrosis, a significant increase (∼6-fold compared to WTCT) takes place. However, *Cpt1a* OE does not affect these changes (Supplementary Table S2; Figure 4).

Cholesterol performs essential cellular functions, and total cellular cholesterol levels are carefully regulated, controlled by its synthesis *de novo*, influx, efflux and subcellular distribution (Zager et al., 2001)*.* The cholesterol which constitutes cellular membranes is not static, so it can be transported as free cholesterol (FC). It can also be enzymatically esterified in the ER, generating CE. These CE can be hydrolyzed both in the cytosol and in lysosome systems to generate FC again, in the so-called “cholesterol ester cycle”. When cellular FC levels increase, CE formation is induced since FC accumulation can induce toxicity. Thus, although CEs are generally considered a form of cytosolic storage of cholesterol, they are also implicated in the regulation of cellular processes (Zager et al., 2001).

In this way, the CE formation could be considered protective. However, in some studies, the increase in the concentration in CE was related to the progression of damage. The inhibition of its producing enzyme reduced the content of CE and protected against kidney damage in DKD and Alport’s syndrome (Liu et al., 2020). In an acute ischemic kidney injury model, CE accumulation in the renal cortex occurs as an immediate consequence of injury. The alterations in CE levels, but not in FC, may indicate a disturbance in the cholesterol ester cycle and in its recycling, suggesting there is specific damage to the plasma membrane (Zager et al., 2001).

## 4 Conclusion

**FIGURE 5:**
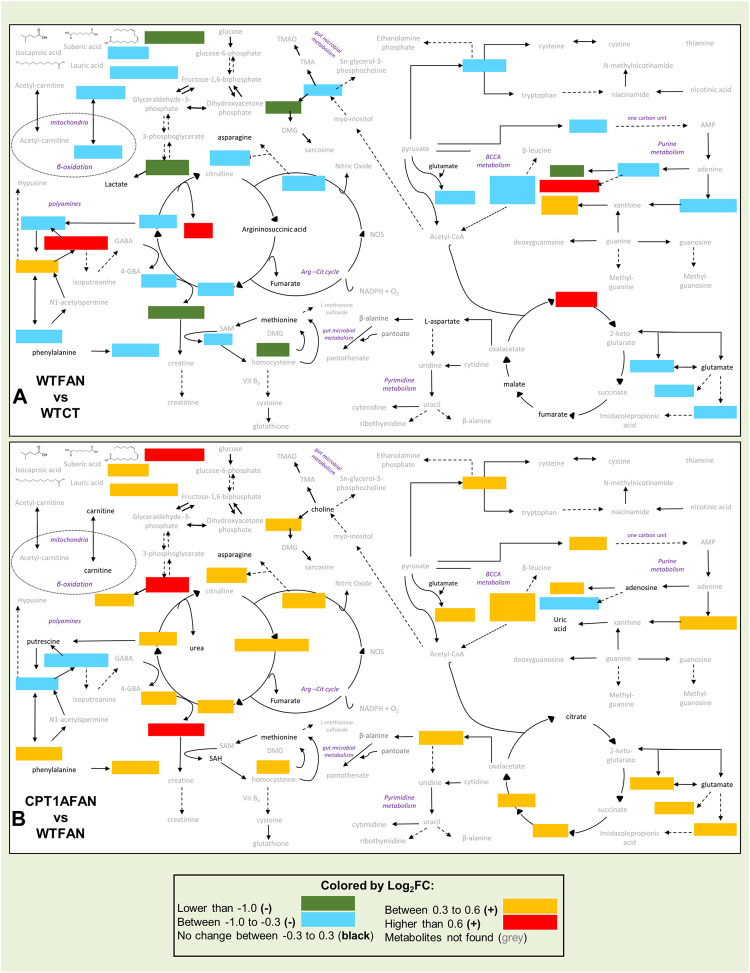
Schematic representation of altered metabolites related to fibrosis and Cpt1a overexpression in **(A)** WTFAN vs. WTCT and **(B)** CPT1AFAN vs. WTFAN comparisons. Metabolism’s general scheme includes the changes in metabolites for each comparison. Metabolites with significant changes are indicated with colors. Metabolites with a Log_2_FC lower than −1.0 are in green color, metabolites with a Log_2_FC between −1.0 and −0.3 are blue color, metabolites with no change are indicated with black names, orange color for metabolites with a Log_2_FC between −0.3 and 0.3, metabolites with a Log_2_FC higher than 0.6 are in red color and finally metabolites not detected in these analyses are represented with grey names.

In this study, we have established a successful untargeted metabolomic multiplatform approach for the analysis of CKD-associated renal fibrosis samples. By this methodology, we have detected multiple metabolic alterations produced by renal fibrosis in the kidney of a murine folic acid nephropathy model. Our findings support that tubulointerstitial fibrosis profoundly impacts the levels of metabolites and lipids involved in important biochemical pathways of renal metabolism (Figure 5A). Additionally, our results show that in this kidney fibrosis model, the overexpression of the *Cpt1a* gene significantly modulates the changes in many compounds related to fibrosis, globally shifting the metabolic profile towards control conditions (Figure 5B). This supports that under kidney damage, overexpression of *Cpt1a* promotes a recovery of the physiological metabolic phenotype. The mechanisms involved in such recovery include the corresponding increase in fatty acid oxidation and preservation of mitochondrial integrity. Although biological assays confirming these mechanisms need to be performed in future studies. Alterations found in the levels of specific compounds (citric acid, acetylspermidine, methyladenosine, cholesteryl esters, triglycerides, glycine-betaine, amino acid metabolism, and hydroxylated and acetylated lysine derivatives) withing fibrosis with and without *Cpt1a* OE, lend the ground to investigate in depth their pathogenetic role in kidney fibrosis.

## Figures and Tables

**Table:** 

AAs	amino acids
ACC	acetyl-CoA carboxylase
Acly	ATP-citrate lyase
Acsm2	acyl-CoA synthetase medium chain family member 2
Ass1	argininosuccinate synthetase
Azin1	antizyme inhibitor
BCAAs	branched-chain amino acids
Bhmt	betaine-homocysteine S-methyltransferase
CE	cholesteryl esters
CE-MS	capillary electrophoresis mass spectrometry
Cer	Ceramides
CKD	Chronic Kidney Disease
Cpt1a	carnitine palmitoyl transferase 1a gene
CPT1A	carnitine palmitoyl transferase 1a protein
CPT1ACT	Cpt1a overexpression control
CPT1AFAN	Cpt1a overexpression folic acid-induced nephropathy
Cs	citrate synthase
CV	coefficient of variation
DG	diacylglycerols
Dgat2	diacylglycerol O-acyltransferase 2
DKD	diabetic kidney disease
ECM	extracellular matrix
EI	electron ionization source
ER	endoplasmic reticulum
ERSR	endoplasmic reticulum stress response
ESI	electrospray ionization source
ESRD	end-stage renal disease
FA	Fatty acids
FAN	folic acid nephropathy
FAO	fatty acid oxidation
FAO-GOF	fatty acid oxidation gain of function
Fasn	fatty acid synthase
FC	free cholesterol
GC-MS	gas chromatography mass spectrometry
Gnmt	glycine N-methyltransferase
HexCer	Hexosylceramides
IRI	ischemia reperfusion injury
IS	internal standard
ISF	in-source fragmentation
JK	jackknife
LC-MS	liquid chromatography mass spectrometry
MAAs	modified amino acids
medianFC_4Gr	median fold change normalization group by group
MT	migration time
MVA	multivariate analysis
Odc1	ornithine decarboxylase
OE	overexpression
PC	glycerophosphocholines
PCA-X	Principal component analysis
*p(corr)*	correlation coefficient *p(corr)*
PE	glycerophosphoethanolamines
PG	glycerophosphoglycerols
PI	glycerophosphoinositols
PKD	polycystic kidney disease
Plod3	procollagen-lysine,2-oxoglutarate 5-dioxygenase 3
PLS-DA	partial least square-discriminant analysis
PS	glycerophosphoserines
QC	quality control
Quant_4Gr	Quantile normalization group by group
RMT	relative migration time
ROS	oxidative stress
RT	retention time
SAH	S-adenosyl homocysteine
SAM	S-adenosyl methionine
SM	sphingomyelins
Sms	spermine synthase
Srebf1	sterol regulatory element-binding protein 1
TCA	tricarboxylic acid
TG	triacylglycerols
UVA	univariate analysis
WTCT	wild type control
WTFAN	wild type folic acid-induced nephropathy

## Data Availability

The original contributions presented in the study are included in the article/Supplementary Material, further inquiries can be directed to the corresponding author.
