# Supplementary material for: Impact of renal tubular Cpt1a overexpression on the kidney metabolome in the folic acid-induced fibrosis mouse model
Source: Front Mol Biosci. 2023 Jun 12;10:1161036. doi: 10.3389/fmolb.2023.1161036 (PMC10291237; doi:10.3389/fmolb.2023.1161036)
Supplement: Supplementary file 1 [file Table1.docx]

2.1 Study design

The study approval was granted by the Centro de Biología Molecular “Severo Ochoa” (CBMSO) Ethics Committee for Animal Experimentation in Madrid, the Consejo Superior de Investigaciones Científicas (CSIC) ethics committee, and the Regulatory Unit for Animal Experimental Procedures from the Comunidad de Madrid (PROEX 098.0/22) (1,2). Mice were housed in the specific pathogen-free animal facility at the CBMSO under EU regulation. The procedure previously published was followed to generate the FAN murine model (3,4).

## 2.2 Multiplatform Untargeted Metabolomics Analysis

### 2.2.1 Chemicals

Organic solvents and chemicals were of analytical grade from Sigma-Aldrich (Germany). Sialylation-grade pyridine was purchased from VWR International BHD Prolabo (Spain). Reference mass solutions for LC-MS and CE-MS were obtained from Agilent Technologies. Finally, deionized water (Milli-Q) used throughout the complete study was obtained by a Milli-Q PLUS system from Millipore (Austria).

2.2.2 Sample collection and processing

In summary the kidney sample treatment applied was, cold methanol 50% was added at a tissue-weight volume ratio of 1:10 for homogenization using TissueLyser LT bead-mill homogenizer device (QIAGEN, Hilden, Germany). The homogenization was carried out with 2.8 mm (mean diameter) steel beads, vibrating at a maximum power of 50Hz for 5 min, during 4 cycles, with a 1-minute break on ice between cycles. The weight range of the kidney tissue varied between 20 and 30 mg (WTCT, 23.5–28.4 mg; WTFAN, 22.7–30.2 mg; CPT1ACT, 17.3– 32.8 mg; CPT1AFAN, 24–31.1 mg). Once the homogenate was obtained, the metabolite extraction process followed different protocols depending on the analytical platform, CE-MS, LC-MS or GC-MS (5–7). For CE-MS analysis, 100 μL of the homogenate was taken into a new Eppendorf tube and added with 100 μL of 0.2 M formic acid, vortex-mixed, and centrifuged (16,000×g 10 min, 4 °C). Then the supernatant was transferred to a Centrifree ultracentrifugation device (Milipore Ireland Ltd., Cork, Ireland) with 30-kDa protein cutoff filter for deproteinization process through centrifugation step (2000×g, 70 min, 4 °C). The filtrate was transferred to a chromacol vial, evaporated by a SpeedVac Concentrator (Thermo Fisher Scientific, Waltham, MA, USA), and finally resuspended in 50 μL of 0.1 M formic acid containing 0.2 mM methionine sulfone as internal standard (IS). For LC-MS and GC-MS analysis, a further 100 μL of homogenate was transferred to a new Eppendorf tube, to which 320 μL of cold methanol was added. Then the samples were vortex-mixed for 2 min and 80 μL of methyl tert-butyl ether was added. Afterwards, samples were vortex-mixed for 1h at room temperature, centrifuged at 4000 g at 20ºC for 20 min, and the supernatants obtained were divided for LC-MS and GC-MS analysis. In the case of LC-MS analysis, 100 μL of supernatant (50 μL for positive mode and 50 μL for negative mode analysis) were taken to inject them directly into the system UHPLC-MS. For GC-MS analysis, 300 μL were transferred to a chromacol vial and evaporated to dryness using a SpeedVac Concentrator system to start the derivatization process. The samples were reconstituted with 20 μL O-methoxyamine hydrochloride (15 ng/mL) in pyridine for the methoximation step. Following vigorous ultrasonication and vortex, the vials were left in the dark at room temperature for a 16-hour incubation. Next for the silylation step, 20 μL of BSTFA:TMCS (99:1) were added, followed by vortex-mixing for 5 minutes, and incubation of 1h at 70ºC in the oven. In the final step 100 μL of heptane containing 20 ppm of tricosane (IS) were added (1,7).

Quality control samples (QCs) were prepared for each analytical technique by pooling equal volumes of each homogenized kidney tissue, together with blank samples were also prepared along with the rest of the experimental samples. Blank samples were analyzed at the beginning and at the end of the analytical sequence. QC samples were injected at the beginning of the sequence to equilibrate and adapt the analysis system, after running 5-6 experimental samples to verify stability and performance of the analysis, and at the end of the analysis sequence.

2.2.3 Untargeted metabolomics fingerprinting by CE-TOF-MS

In brief, the conditions used in the CE-MS analysis were, a fused-silica capillary (Agilent Technologies; total length 96 cm; i.d.; 50 μm), previously flushed for 5 min with background electrolyte (BGE) (1 M formic acid in 10% methanol solution), was used for the separation analysis. Samples were injected over 50s at 50mbar, and BGE was co-injected for 20s at 100mbar after each sample injection to improve the reproducibility of the analysis. The composition of the sheath liquid included methanol/water (1/1, v/v), formic acid (1 mM), and two reference masses (purine, m/z 121.050873; HP-0921, m/z 922.009798). The total time of the analytical run was 23 min. The separation was carried out at a pressure of 25 mbar and a voltage of +30kV, in positive ionization mode, with a flow rate of 0.6 mL/min and a split set to 1/100. The MS was operated in positive polarity mode, with a full scan range from m/z 70 to 1000 at a rate of 1.36 scan/s. The drying gas was set to 10 L/min at 200 ºC temperature, nebulizer to 10 psi, voltage to 3.5 kV, fragmentor to 125 V and skimmer to 65 V (1).

2.2.4 Untargeted metabolomics fingerprinting by UHPLC-QTOF-MS

For the LC-MS analysis, 1.5 μL of samples were injected with a multiwash option using the Agilent 1290 Infinity II Multisampler system. During the analytical run, samples were kept at 15ºC in the sampler to avoid lipid precipitation or compound degradation. Reversed-phase chromatography was used with an InfinityLab Poroshell 120 EC-C8 (2.1 x 150 mm, 2.7 µm) (Agilent Technologies) column and with a suitable guard column (UHPLC Guard 3PK) (Agilent Technologies) maintained at 60ºC. Mobile phases for the positive ionization mode were composed by an for aqueous phase (solvent A) of 10 mM ammonium formate in Milli-Q water and an organic phase (solvent B) of 10mM ammonium formate in methanol/isopropanol (85/15, v/v) with a flow rate of 0.5 mL/min. For negative ionization mode, solvent A was 0.1% formic acid in Milli-Q water and solvent B was 0.1% formic acid in methanol/isopropanol (85/15, v/v). The mobile phases gradient started at 75% of solvent B, increasing to 96% B at minute 23 and kept for 8 min. The gradient then increased to 100% of solvent B by minute 31.5 and was maintained until minute 32.5. At minute 33, the initial condition was returned, followed by a 7 min-re-equilibration time, with a total run time of 40 min. In this analysis, a multiwash strategy with three solvents was applied, using as S1 a mixture of mobile phases A and B (25/75, v/v) to come back to starting conditions, S2 a mixture of methanol and water (1:1), and as S3 isopropanol, with a wash time set at 60 seconds. The parameters set in the mass spectrometer for the analysis in the positive and negative mode were as follows: 3500 V capillary voltage, 175 V fragmentor, 65 V skimmer, 750 V octupole radio frequency voltage, 11 L/min for drying gas flow rate at 290 ºC gas temperature, and 40 psi nebulizer pressure, 11L/min sheath gas flow, and 370ºC sheath gas temperature. Data were collected in positive and negative ESI modes in separate runs and the centroid mode at a scan rate of 1.0 scan/s. The MS operated in full scan mode from 100 to 1700 m/z. The reference masses used over the whole analysis were m/z 121.0509 (protonated purine), m/z 149.0233 (protonated phtalic anhydride), and m/z 922.0098 (protonated HP-921) for positive ionization mode. Instead, for negative ionization mode were m/z 112.9856 (proton-abstracted TFA anion) and m/z 966.0007 (formate adduct of HP-921). An automated Calibrant Delivery System (CDS) constantly infused these reference masses at a 0.8 mL/min flow rate to allow constant mass correction, using a Dual Agilent Jet Stream Electrospray Ionization (Dual AJS ESI) source for continuously introducing calibrant solution. At the end of the analytical sequence, 10 iterative-MS/MS runs were performed with an MS and MS/MS scan rate of 3 spectra/s, 50-1700 m/z mass window, a narrow (~ 1.3 amu) MS/MS isolation width, and a cycle time of 3.1 s, an MS/MS threshold of 5000 counts and 0.001% and with collision energy set in 20 V (for 5 iterative runs) and 40V (for other 5 iterative runs). Reference masses and contaminants present in blanks were excluded from Iterative-MS/MS analysis (8).

2.2.5 Untargeted metabolomics fingerprinting by GC-QTOF-MS

In Summary, 1 μL of the derivatized sample was injected through an Agilent DB5-MS GC Capillary Column (30 m length, 0.25 mm, 0.25 µm film 95% dimethylpolysiloxane/5% diphenylpolysiloxane) using an Agilent autosampler (7693A). The samples were injected in a split ratio of 1:10 into a Restek 20782 deactivated glass-wool split liner. The injector port was established at 250ºC, the flow rate of helium carrier gas was set at 1 mL/min through the column. The temperature gradient was programmed at 60ºC as initial oven temperature (maintained for 1 min), increasing until 325ºC at a rate of 10ºC per minute. This temperature was kept for 10 min before cooling down. The retention time lock (RTL) relative to the internal standard C18:0 methyl ester peak was established at 19.66 min. The total time of the analysis run was 37.5 minutes. The parameters used in the Agilent 7250 QTOF mass spectrometer system were as follows: an electron ionization (EI) source set at -70 eV EI energy, 200ºC in the filament source temperature, 280ºC in the detector transfer line temperature, and 150ºC as quadrupole temperature. Finally, the mass spectrometer collected data within a 40-600 m/z range at a scan rate of 10 spectra/s (6,7).

2.2.6 Data Analysis

*2.2.6.1 Quality assurance procedure*

Further evaluation was carried out with Hotelling’s T2 Range Plot on the PCA-X model itself. Moreover, data matrix was used to perform control charts by plotting the total acquired signals versus sample acquisition one in order to check analytical precision. The variation within measurements was obtained by calculating the coefficient of variation (CV) of the QCs and the experimental sample groups. The different quality control and quality assurance procedures were carried out throughout the data processing steps (1,9).

The PCA-X scores plots presented a natural separation along the first component in all plots between the control (CT) groups (WTCT and CPT1ACT) and the group of WT mice with fibrosis induced by folic acid (WTFAN). However, the samples from the group with Cpt1a OE and fibrosis (CPT1AFAN) were scattered together with CT and WTFAN groups (Figure 2_Supp).


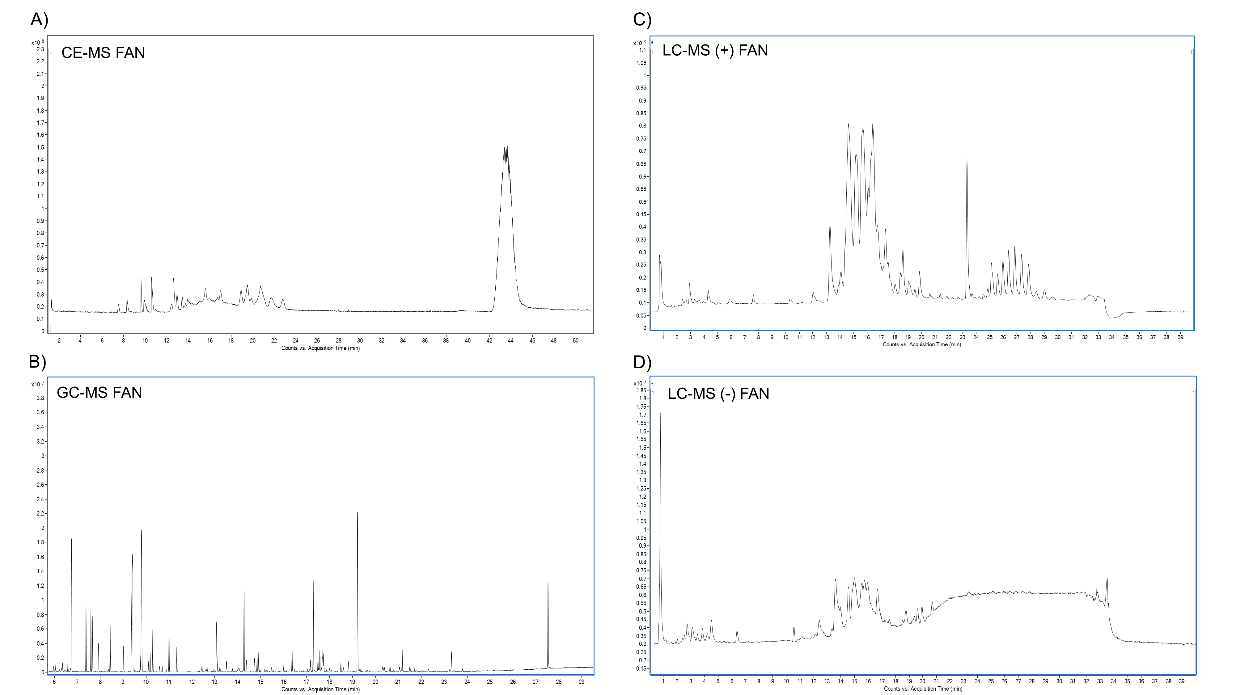


**Figure 1_Supp. Representative chromatograms of FAN model kidney tissue metabolic fingerprints measured by CE-MS, GC-MS, and LC-MS in positive and negative ESI mode analyses.** Figures A), B), C) and D) are representative total ion chromatograms and electropherograms belonging to a pool QC sample.

*2.2.6.2 GC-MS data pre-processing*

In the case of GC-MS data, MassHunter Workstation GC-MS Translator (B.04.01) was used to convert the raw data files into the appropriate format files for quantitative analysis. The spectral deconvolution and metabolite identification steps were carried out in the MassHunter Unknown Analysis (Tool 9.0. Agilent Technologies). This software searched in one commercial library, the Fiehn library version 2013, and in an “in-house” spectral library from CEMBIO based on Fiehn and NIST libraries. It was built with the Agilent PCDL Manager (B.08R.00) to assign a chemical identity to each signal detected. These identities are based on the comparison of RT, retention indices and spectra between the experimental information obtained in the deconvolution with the information of each compound present in the libraries. The obtained data were aligned and filtered with Mass Profiler Professional software (Version B.12.1, Agilent Technologies). Furthermore, these identities were exported to Agilent MassHunter Quantitative Analysis software (Version B.09.00, Agilent Technologies), where target ion and qualifiers were assigned to them. The integration and manual inspection of peaks were carried out to obtain the final data matrix with the abundance of each metabolite (10,11).

*2.2.6.3 LC-MS and CE-MS data pre-processing*

Data acquired from LC-MS (positive and negative modes) and CE-MS analysis were pre-processed with Agilent MassHunter Profinder Software (Ver. B.08.00 and Ver. B.10.00, Agilent Technologies). This software performs two steps to reduce data's complexity, remove background noises and unrelated ions to identify important variables (features) associated with data. The first step is the software's Molecular Feature Extraction (MFE) tool; this algorithm performs the deconvolution process of chromatograms to find all features in the data and aligns all ions across samples files using mass and retention time (RT) or migration time (MT) information. In this way, a single spectrum (related co-eluting ion signals as isotopes, common adducts, dimers or neutral loss are grouped) for each compound group is created and used in the next step. The following tool used in this software is Recursive Feature Extraction. This step takes the information generated in MFE and, using the Find by Ion (FbI) algorithm, improves the quality and reliability of the list of identified compounds, eliminating nonspecific information, and extracting the most important signals. In this way, a structured data matrix is created with the appropriate format and minimum false-positive and false-negative results. For LC-MS positive, LC-MS negative and CE-MS, data matrices were extensively cleaned by manual inspection, using Mass Hunter Profinder Software (Agilent Technologies) and Mass Hunter Qualitative Analysis software (Agilent Technologies) to check the quality of each metabolic feature (9,11,12).

*2.2.6.4 Data pre-treatment and statistical analysis*

When the GC-MS, LC-MS+, LC-MS-, and CE-MS matrices were in the Microsoft Excel program, blank subtraction and curation of the matrix removing detected duplicate features, different adducts from the same signal, salt clusters and fragment ions in CE-MS data to eliminate irrelevant information that could interfere in the data treatment process were carried out. In the data pre-treatment workflow, metabolic features detected in less than 50% of QC samples were excluded from the data matrix. Missing values in experimental samples were imputed using the K-nearest neighbors (kNN) algorithm (13). For the normalization step, a study of different normalization strategies was performed on each matrix due to the heterogeneity of the renal tissue and the presence of fibrosis (1,14). A multilevel normalization method was applied through a pre-acquisition normalization step during sample treatment, a signal drift correction using QC samples or IS (in case it was necessary), and finally a post-acquisition normalization strategy to remove unwanted biological variation and to reduce intra-group variability, applied as group-dependent method (4Gr) (1). Specifically, in the data of CE-MS a quantile normalization method was applied group by group (Quant_4Gr). The GC-MS matrix was normalized first by the IS (tricosane) and later using a Quant_4Gr mathematical normalization method. Finally, in both matrices from LC-MS, a median fold change method was applied group by group (medianFC_4Gr). Once the matrices were normalized and filtered, they were imported into SIMCA P+16 (Umetrics®, Umea, Sweden) for multivariate statistical analysis (MVA). Firstly, each matrix was scaled and transformed. Then, the PCA-X, and PLS-DA (Figure 2_Supp and Figure 3_Supp) models were constructed. All of them were constructed using the first predictive component and the first orthogonal component. First an unsupervised PCA-X model (Figure 2_Supp) was performed to have an initial overview of the stability and reliability of the data and to check preliminary trends and clustering in the experimental samples. Later, the supervised method partial least square-discriminant analysis (PLS-DA) was performed. The PLS-DA models reveal the global metabolic changes due to kidney fibrosis and the overexpression of the *Cpt1a* gene, and its quality is represented by the parameters R^2^ (explained variance) and Q^2^ (capability of prediction) (Figure 3_Supp).

*2.2.6.5 Metabolite identification*

In the database search, matched compounds were tentatively assigned based on accurate mass with a maximum mass error of 20 ppm, isotopic pattern distribution, the possibility of cation and anion formation, adduct formation and retention time or migration time based on the separation conditions. The MS/MS data analyzed by using the Lipid Annotator tool (Agilent Technologies). Putative results were manually checked by reviewing the MS/MS spectra and, if necessary, by comparing with available MS/MS spectra, including those of Metlin and Lipidmaps. For CE-MS data, a range of different voltages applied in the analysis was used to obtain in-source ion fragmentation characteristic patterns of the different metabolites.

2.2.7 RNA extraction and TaqMan gene expression analysis

The quality and quantity of RNA extracted were determined at 260 nm by a Nanodrop-1000 spectrophotometer (Thermo Fisher Scientific). Five hundred ng of total RNA extracted were processed to reverse transcription using the High-Capacity cDNA Reverse Transcription Kit (Thermo Fisher Scientific). PCR amplification was performed using TaqMan master (Thermo Fisher Scientific) with the Roche LightCycler 480 Real-Time PCR system (AB7900HT). The ABI TaqMan SDS v2.4 software was utilized to obtain Cq values for each gene. The Cq data were analyzed with Sat-Miner 4.2.8 software (Integromics). The ΔCt value was calculated by normalizing Ct values to the endogenous housekeeping gene GAPDH. Relative mRNA expression was determined using the 2−ΔΔCt method (15) and fold changes were normalized to the wildtype control condition values. The difference between two independent groups was examined with the Mann-Whitney U test. The p-value obtained was adjusted by the False Discovery Rate (FDR) obtaining q-value. A Q-value of 0.05 or less was considered statistically significant. All the TaqMan analysis procedures were performed in the Genomics Facility of the Fundación Parque Científico de Madrid.

**
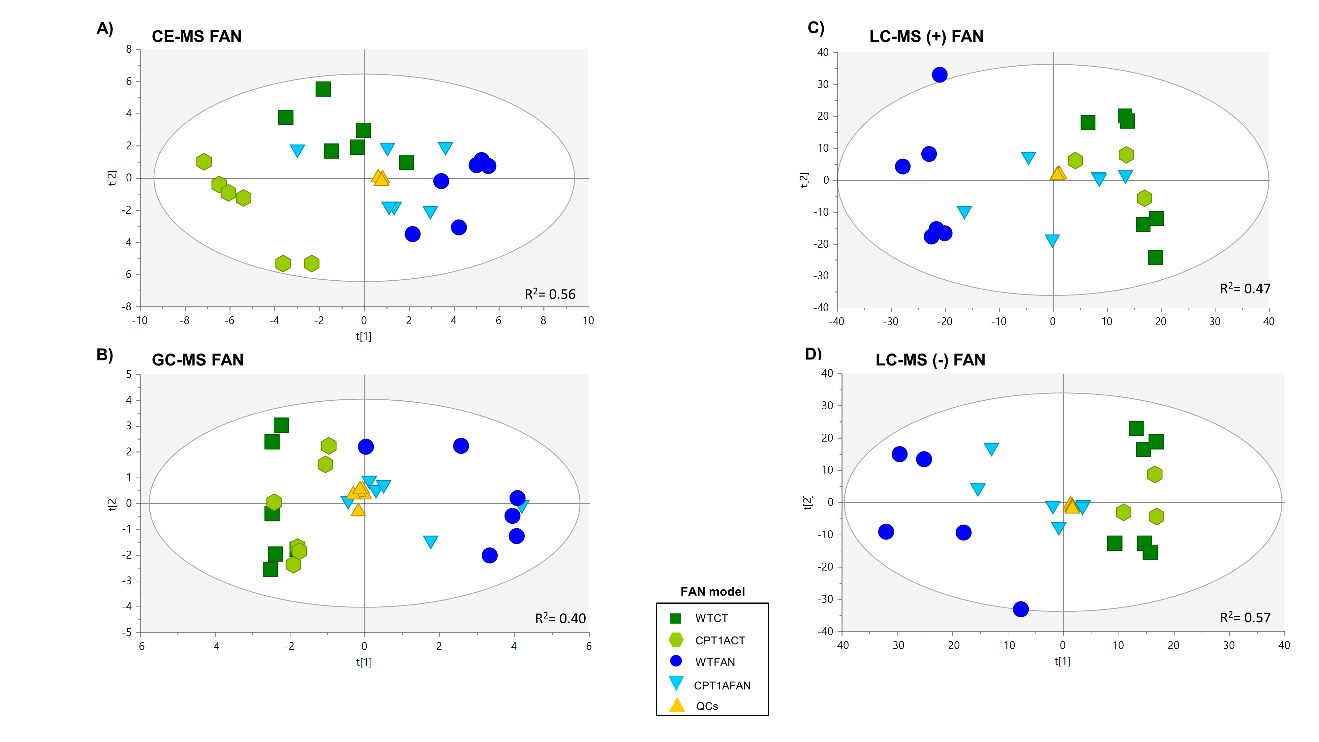
**

**Figure 2_Supp. PCA score plots for the three analytical platforms. A)** CE-MS data with Log_2_ transformation and Pareto scaling; a two-component model explains 38% (R_2_X=0.380) and 56% (R_2_X=0.559) variation in the 1^st^ and the 2^nd^ component *t_1_* and *t_2_*, respectively **B)** GC-MS data with Log_2_ transformation and Pareto scaling; a two-component model explains 26% (R_2_X=0.263) and 39% (R_2_X=0.392) variation in the 1^st^ and the 2^nd^ component *t_1_* and *t_2_*, respectively **C)** LC-MS (+) data with Log_2_ transformation Unit-variance (UV) scaling, a two-component model explains 26% (R_2_X=0.260) and 47% (R_2_X=0.474) variation in the 1^st^ and the 2^nd^ component *t_1_* and *t_2_*, respectively; **D)** LC-MS (-) data with Log_2_ transformation Unit-variance (UV) scaling, a two-component model explains 33% (R_2_X=0.326) and 57% (R_2_X=0.566) variation in the 1^st^ and the 2^nd^ component *t_1_* and *t_2_*, respectively.

**
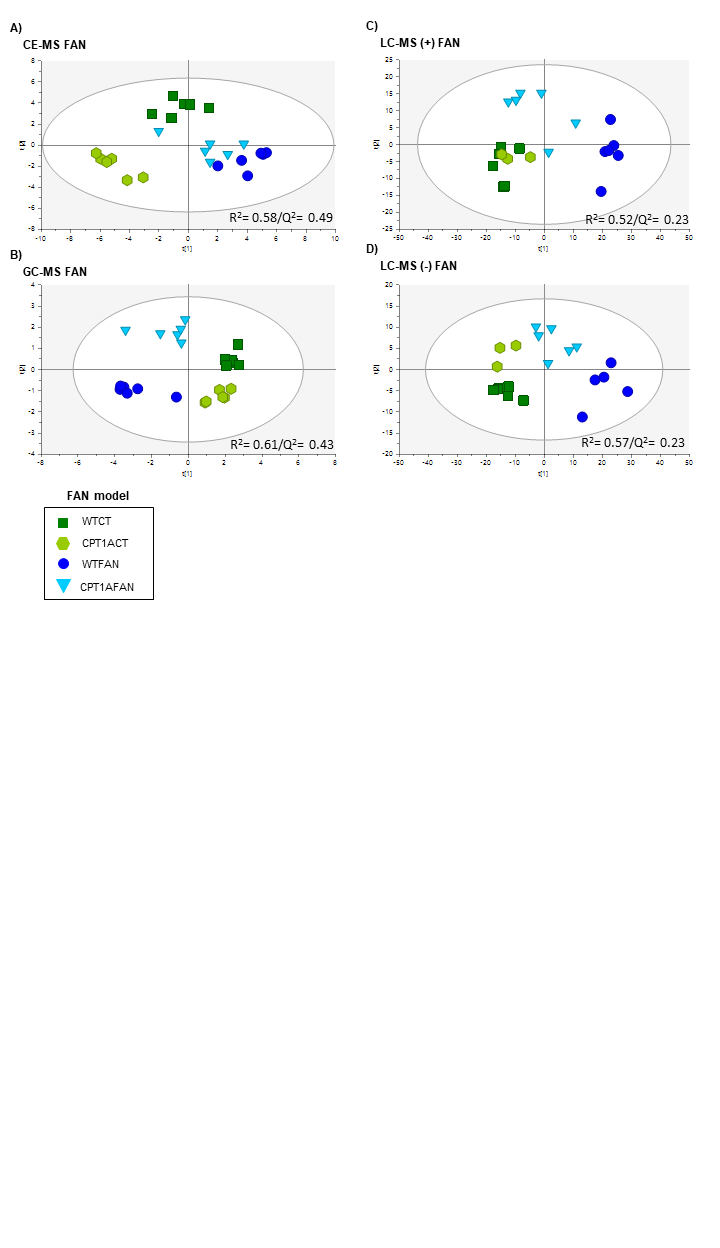
**

**Figure 3_Supp. Supervised PLS-DA score plots of each analytical platform for the four experimental groups.** PLS-DA models have been built on normalized data and after applying the same scaling and/or transformation in PCA-X plots. The legend for the experimental groups is: dark green squares to WTCT group, light green hexagons to CPT1ACT group, dark blue circles to WTFAN and light blue inverted triangles to CPT1AFAN. **A)** PLS-DA CE-MS for the FAN model with Pareto scale and Logarithm transformation (R2X(cum) 0.549 R2Y(cum) 0.577 Q2(cum) 0.485); **B)** PLS-DA GC-MS for the FAN model with Pareto scale and Logarithm transformation (R2X(cum) 0.378, R2Y(cum) 0.606 Q2(cum) 0.429); **C)** PLS-DA LC-MS positive for the FAN model with Unit-variance (UV) scale (R2X(cum) 0.422, R2Y(cum) 0.518 Q2(cum) 0.23); **D)** PLS-DA LC-MS negative for the FAN model with UV scale and Logarithm transformation (R2X(cum) 0.407, R2Y(cum) 0.568 Q2(cum) 0.233).

**A)**


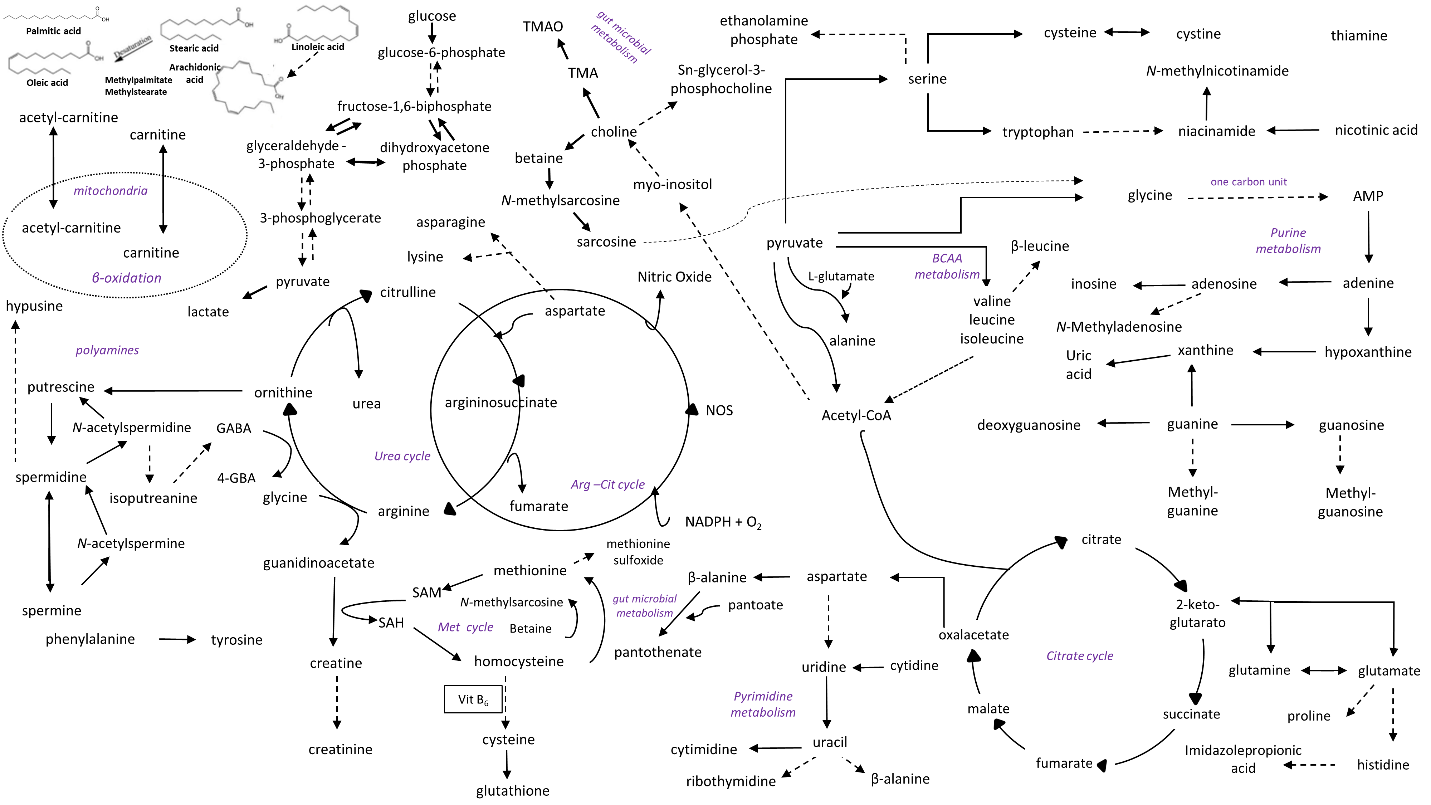


**B)**


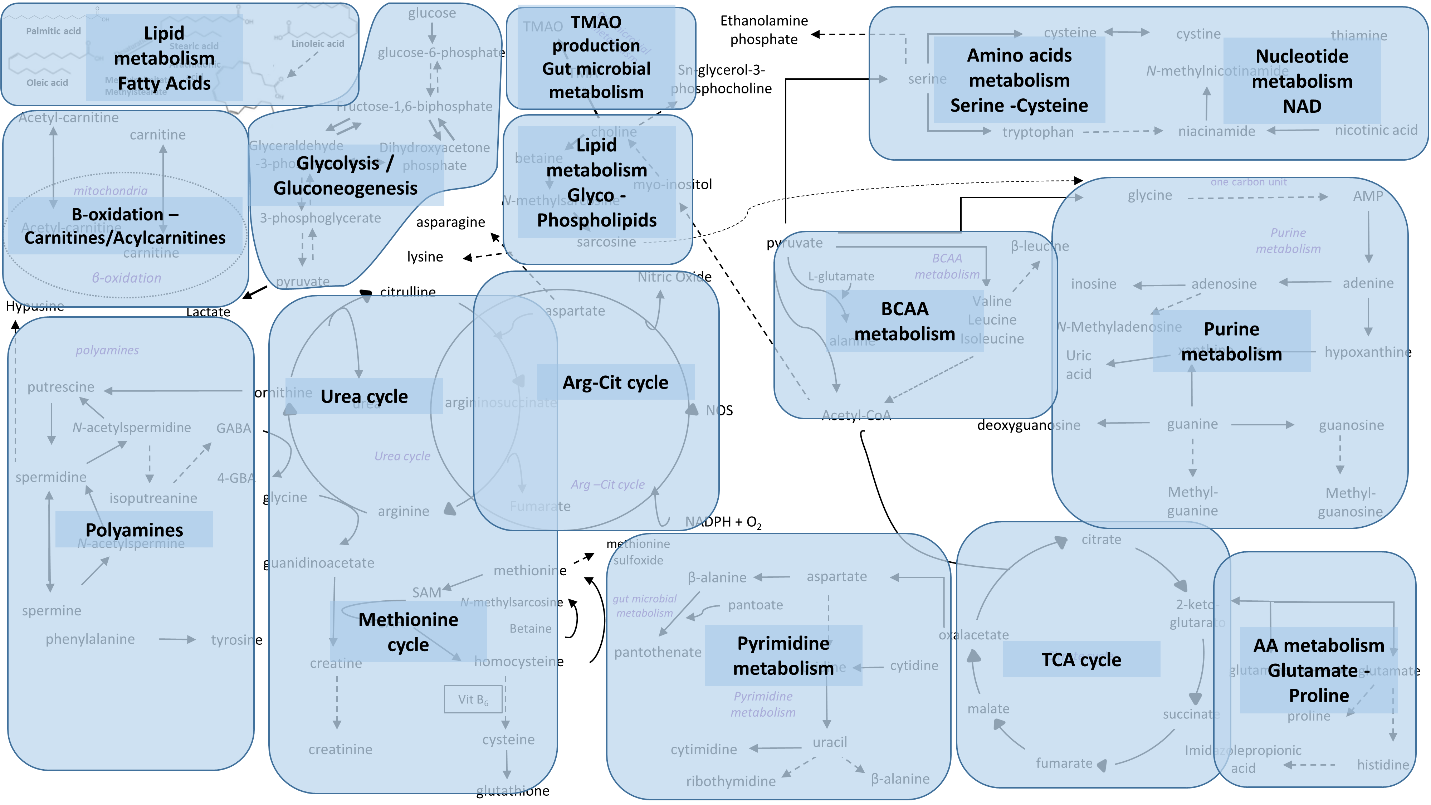


**Figure 4_Supp. Schematic representation of altered metabolites related to fibrosis and expected FAO-GOF.** A) Metabolism’s general scheme of metabolites with significant changes obtained in the different comparisons of both fibrosis biological models. B) Grouping of altered metabolites into different metabolic pathways and cycles in which the altered metabolites are involved.

**Table 1_Supp. Annotated metabolites found as statistically significant at any of the comparisons performed between the different groups in the FAN fibrosis model.**

| **Compound** | **Mass** | **RT** | **RMT** | **Formula** | **Analytical Platform** | **ESI mode** | **Adduct** | **Mass Error** | **ID** | **ID** | **QCs** |
| --- | --- | --- | --- | --- | --- | --- | --- | --- | --- | --- | --- |
|  |  |  |  |  |  |  |  |  | **Source** | **Level** | **CV (%)** |
| TCA cycle | | | | | | | | | | | |
| Pyruvic acid | 88.016 | 6.58 | -- | C3H4O3 | GC-MS | -- | -- | -- | F | 2 | 25.2 |
| Lactic acid | 90.0318 | 6.73 | -- | C3H6O3 | GC-MS | -- | -- | -- | F | 2 | 4 |
| Citric acid | 192.027 | 16.48 | -- | C6H8O7 | GC-MS | -- | -- | -- | F | 2 | 5.6 |
| Fumaric acid | 116.0109 | 10.84 | -- | C4H4O4 | GC-MS | -- | -- | -- | F | 2 | 7.6 |
| Malic acid | 134.0215 | 12.68 | -- | C4H6O5 | GC-MS | -- | -- | -- | F | 2 | 7.9 |
| Urea cycle & Citrulline -NO cycle | | | | | | | | | | | |
| Argininosuccinic acid | 272.1127 | 15.49 | 0.7 | C10H16N4O5 | CE-MS | (+) | [M+H]+ | 12 | ISF | 3 | 3.3 |
| Arginine | 174.1117 | 13.1 | 0.59 | C6H14N4O2 | CE-MS | (+) | [M+H]+ | 4 | ISF | 2 | 2.3 |
| Urea | 60.0324 | 9.39 | -- | CH4N2O | GC-MS | -- | -- | -- | F | 2 | 0 |
| Ornithine | 132.0899 | 12.52 | 0.56 | C5H12N2O2 | CE-MS | (+) | [M+H]+ | 2 | ISF | 2 | 1.4 |
| Glycine | 75.0321 | 15.21 | 0.68 | C2H5NO2 | CE-MS | (+) | [M+H]+ | 10 | ISF | 2 | 2.2 |
| Guanidinoacetate | 117.0544 | 15.08 | 0.68 | C3H7N3O2 | CE-MS | (+) | [M+H]+ | 9 | ISF | 2 | 4.3 |
| Folate cycle & Methionine cycle | | | | | | | | | | | |
| Methionine | 149.0527 | 19.77 | 0.89 | C5H11NO2S | CE-MS | (+) | [M+H]+ | 3 | ISF | 2 | 7.4 |
| S-adenosyl-homocysteine | 384.1226 | 16.19 | 0.73 | C14H20N6O5S | CE-MS | (+) | [M+H]+ | 11 | ISF | 2 | 3.9 |
| *N,N,N*-trimethyl-glycine (Betaine) | 117.079 | 21.13 | 0.95 | C5H12NO2 | CE-MS | (+) | [M+H]+ | 4 | ISF | 2 | 0 |
| Choline | 104.1071 | 12.32 | 0.55 | C5H14NO | CE-MS | (+) | [M+H]+ | 11 | ISF | 2 | 0 |
| Amino acids | | | | | | | | | | | |
| Alanine | 89.048 | 16.57 | 0.75 | C3H7NO2 | CE-MS | (+) | [M+H]+ | 14 | ISF | 3 | 2.6 |
| Proline | 115.0633 | 20.05 | 0.9 | C5H9NO2 | CE-MS | (+) | [M+H]+ | 9 | ISF | 2 | 1.2 |
| Valine | 117.0791 | 18.45 | 0.83 | C5H11NO2 | CE-MS | (+) | [M+H]+ | 7 | ISF | 3 | 2.5 |
| Isoleucine/Leucine | 131.0944 | 19 | 0.85 | C6H13NO2 | CE-MS | (+) | [M+H]+ | 1 | ISF | 3 | 0 |
| Histidine | 155.0696 | 13.4 | 0.6 | C6H9N3O2 | CE-MS | (+) | [M+H]+ | 5 | ISF | 2 | 2.1 |
| Phenylalanine | 165.0791 | 20.48 | 0.92 | C9H11NO2 | CE-MS | (+) | [M+H]+ | 9 | ISF | 2 | 0.5 |
| Tyrosine | 181.0739 | 21.08 | 0.95 | C9H11NO3 | CE-MS | (+) | [M+H]+ | 3 | ISF | 2 | 1.4 |
| Asparagine | 132.0521 | 19.31 | 0.87 | C4H8N2O3 | CE-MS | (+) | [M+H]+ | 6 | ISF | 2 | 3.7 |
| Lysine | 146.1056 | 12.62 | 0.57 | C6H14N2O2 | CE-MS | (+) | [M+H]+ | 1 | ISF | 2 | 9.1 |
| Serine | 105.0428 | 18.38 | 0.83 | C3H7NO3 | CE-MS | (+) | [M+H]+ | 8 | ISF | 2 | 0 |
| Glutamine | 146.0686 | 19.94 | 0.9 | C5H10N2O3 | CE-MS | (+) | [M+H]+ | 0 | ISF | 2 | 0 |
| Glutamic | 147.0536 | 20.24 | 0.91 | C5H9NO4 | CE-MS | (+) | [M+H]+ | 1 | ISF | 2 | 0 |
| Aspartic acid | 133.0373 | 21.35 | 0.96 | C4H7NO4 | CE-MS | (+) | [M+H]+ | 5 | ISF | 2 | 0 |
| Modified Amino Acids (MAAs) | | | | | | | | | | | |
| 5-hydroxy-lysine | 162.1009 | 13.17 | 0.59 | C6H14N2O3 | CE-MS | (+) | [M+H]+ | 4 | ISF | 2 | 5.6 |
| N2-Acetyl-lysine | 188.1159 | 18.18 | 0.82 | C8H16N2O3 | CE-MS | (+) | [M+H]+ | 2 | ISF | 2 | 5 |
| Polyamines | | | | | | | | | | | |
| Putrescine | 88.1002 | 8.46 | 0.38 | C4H12N2 | CE-MS | (+) | [M+H]+ | 3 | ISF | 2 | 4.2 |
| Spermidine | 145.1579 | 8.17 | 0.37 | C7H19N3 | CE-MS | (+) | [M+H]+ | 4 | ISF | 2 | 4.1 |
| Spermine | 202.2166 | 8.1 | 0.37 | C10H26N4 | CE-MS | (+) | [M+H]+ | 0 | ISF | 2 | 6.4 |
| *N1*-acetylspermidine | 187.1678 | 11.52 | 0.52 | C9H21N3O | CE-MS | (+) | [M+H]+ | 3 | ISF | 3 | 3.7 |
| Purines metabolism | | | | | | | | | | | |
| Adenosine | 267.0969 | 18.34 | 0.83 | C10H13N5O4 | CE-MS | (+) | [M+H]+ | 3 | ISF | 2 | 1.3 |
| *N*-methyl-adenosine | 281.1127 | 18.43 | 0.83 | C11H15N5O4 | CE-MS | (+) | [M+H]+ | 17 | ISF | 3 | 4.5 |
| Hypoxanthine | 136.0385 | 16.37 | -- | C5H4N4O | GC-MS | -- | -- | -- | F | 2 | 4.3 |
| Inosine | 268.0805 | 23.29 | -- | C10H12N4O5 | GC-MS | -- | -- | -- | F | 2 | 3.9 |
| Uric acid | 168.0283 | 19.27 | -- | C5H4N4O3 | GC-MS | -- | -- | -- | F | 2 | 10.9 |
| Fatty acyls | | | | | | | | | | | |
| Carnitine and Acylcarnitines | | | | | | | | | | | |
| Carnitine | 161.1052 | 15.58 | 0.7 | C7H15NO3 | CE-MS | (+) | [M+H]+ | 3 | ISF | 2 | 1.6 |
| Tetradecanoyl-carnitine (CAR 14:0) | 371.3035 | 1.88 | -- | C21H41NO4 | LC-MS | (+) | [M+H]+ | 4 | LA | 2 | 1.8 |
| oleoylcarnitine (CAR 18:1) | 425.3505 | 3.09 | -- | C25H47NO4 | LC-MS | (+) | [M+H]+ | 1 | LA | 2 | 2.8 |
| linoleoylcarnitine (CAR 18:2) | 423.3348 | 2.44 | -- | C25H45NO4 | LC-MS | (+) | [M+H]+ | 11 | LA | 2 | 4.7 |
| Arachidonoyl-carnitine (CAR 20:4) | 447.3349 | 2.46 | -- | C27H45NO4 | LC-MS | (+) | [M+H]+ | 3 | LA | 2 | 1.7 |
| Cervonyl carnitine (CAR 22:6) | 471.3348 | 2.41 | -- | C29H45NO4 | LC-MS | (+) | [M+H]+ | 3 | LA | 2 | 3.3 |
| Fatty acids and conjugates | | | | | | | | | | | |
| Myristic acid (FA 14:0) | 228.3709 | 16.84 | -- | C14H28O2 | GC-MS | -- | -- | -- | F | 2 | 8.1 |
| Palmitic acid (FA 16:0) | 256.4241 | 18.82 | -- | C16H32O2 | GC-MS | -- | -- | -- | F | 2 | 7 |
| Stearic acid (FA 18:0) | 284.2715 | 20.61 | -- | C18H36O2 | GC-MS | -- | -- | -- | F | 2 | 12 |
| Linoleic acid (FA 18:2) | 280.2402 | 20.37 | -- | C18H32O2 | GC-MS | -- | -- | -- | F | 2 | 5.1 |
| Docosapentaenoic acid (FA 22:5) (DPA) | 330.2553 | 5.4 | -- | C22H34O2 | LC-MS | (-) | [M-H]- | 2 | DB | 3 | 10 |
| Fatty esters | | | | | | | | | | | |
| Methylpalmitate | 270.2558 | 17.44 | -- | C17H34O2 | GC-MS | -- | -- | -- | F | 2 | 3.9 |
| Methylarachidonate | 318.2567 | 6 | -- | C21H34O2 | LC-MS | (+) | [M+H]+ | 0 | LA | 2 | 2.1 |
| Glycerophospholipids | | | | | | | | | | | |
| Glycerophosphocholines | | | | | | | | | | | |
| *Monoacylglycerophosphocholines* | | | | | | | | | | | |
| LPC(P-16:0) | 479.3393 | 3.56 | -- | C24H50NO6P | LC-MS | (+) | [M+H]+ | 4 | LA | 2 | 4.1 |
| LPC(20:3) | 545.348 | 2.79 | -- | C28H52NO7P | LC-MS | (+) | [M+H]+ | 0 | LA | 2 | 7.8 |
| LPC(20:4) | 543.3329 | 2.42 | -- | C28H50NO7P | LC-MS | (+)/(-) | [M+H]+ | 1 | LA | 2 | 8.8 |
| LPC(22:6) | 567.3323 | 2.38 | -- | C30H50NO7P | LC-MS | (+)/(-) | [M+H]+ | 0 | LA | 2 | 12.7 |
| LPC(22:6) | 567.332 | 2.56 | -- | C30H50NO7P | LC-MS | (+)/(-) | [M+H]+ | 1 | LA | 2 | 14.6 |
| *Diacylglycerophosphocholines* | | | | | | | | | | | |
| PC(P-38:3) | 795.6149 | 17.46 | -- | C46H86NO7P | LC-MS | (+) | [M+H]+ | 1 | DB | 3 | 5.9 |
| PC(34:3) | 755.5457 | 13.85 | -- | C42H78NO8P | LC-MS | (+) | [M+H]+ | 1 | LA | 2 | 0.4 |
| PC(P-36:1) | 771.6138 | 17.26 | -- | C44H86NO7P | LC-MS | (+) | [M+H]+ | 0 | DB | 3 | 8.5 |
| PC(38:2) | 813.6255 | 17.74 | -- | C46H88NO8P | LC-MS | (+)/(-) | [M+H]+ | 1 | LA | 2 | 16.7 |
| PC(38:3) | 811.6105 | 16.95 | -- | C46H86NO8P | LC-MS | (+)/(-) | [M+H]+ | 2 | LA | 2 | 1.8 |
| PC(38:5) | 807.5739 | 15.21 | -- | C46H82NO8P | LC-MS | (+)/(-) | [M+H]+ | 5 | LA | 2 | 1 |
| PC(38:6) | 805.5633 | 14.48 | -- | C46H80NO8P | LC-MS | (+)/(-) | [M+H]+ | 2 | LA | 2 | 1.2 |
| PC(38:7) | 803.5443 | 13.5 | -- | C46H78NO8P | LC-MS | (+)/(-) | [M+H]+ | 3 | LA | 2 | 2.5 |
| PC(39:4) | 823.6114 | 17.22 | -- | C47H86NO8P | LC-MS | (+)/(-) | [M+H]+ | 3 | LA | 2 | 1.5 |
| PC(39:6) | 819.576 | 15.38 | -- | C47H82NO8P | LC-MS | (+)/(-) | [M+H]+ | 2 | LA | 2 | 4.9 |
| PC(40:5) | 835.6078 | 16.77 | -- | C48H86NO8P | LC-MS | (+)/(-) | [M+H]+ | 1 | LA | 2 | 6.9 |
| PC(40:5) | 835.6087 | 17.14 | -- | C48H86NO8P | LC-MS | (+)/(-) | [M+H]+ | 0 | LA | 2 | 7.4 |
| PC(40:6) | 833.5905 | 16.21 | -- | C48H84NO8P | LC-MS | (+)/(-) | [M+H]+ | 3 | LA | 2 | 1.6 |
| PC(40:7) | 831.5789 | 14.71 | -- | C48H82NO8P | LC-MS | (+)/(-) | [M+H]+ | 1 | LA | 2 | 1.5 |
| PC(40:7) | 831.58 | 15.02 | -- | C48H82NO8P | LC-MS | (+)/(-) | [M+H]+ | 3 | LA | 2 | 1.3 |
| PC(40:8) | 829.5628 | 13.92 | -- | C48H80NO8P | LC-MS | (+)/(-) | [M+H]+ | 1 | LA | 2 | 3.9 |
| PC(40:8) | 829.5592 | 14.47 | -- | C48H80NO8P | LC-MS | (+)/(-) | [M+H]+ | 3 | LA | 2 | 3.4 |
| PC(42:7) | 859.6054 | 16.61 | -- | C50H86NO8P | LC-MS | (+)/(-) | [M+H]+ | 4 | LA | 2 | 9.3 |
| PC(44:4) | 893.6944 | 20.78 | -- | C52H96NO8P | LC-MS | (+)/(-) | [M+H]+ | 8 | LA | 2 | 17 |
| PC(38:6) | 805.564 | 14.08 | -- | C46H80NO8P | LC-MS | (+)/(-) | [M+H]+ | 2 | LA | 2 | 3.3 |
| Glycerophosphoethanolamines | | | | | | | | | | | |
| *Monoacylglycerophosphoethanolamines* | | | | | | | | | | | |
| LPE(16:0) | 453.2833 | 2.94 | -- | C21H44NO7P | LC-MS | (+) | [M+H]+ | 5 | LA | 2 | 3.6 |
| LPE(18:0) | 481.317 | 4.31 | -- | C23H48NO7P | LC-MS | (+) | [M+H]+ | 1 | LA | 2 | 1.5 |
| LPE(18:1) | 479.301 | 3.33 | -- | C23H46NO7P | LC-MS | (+) | [M+H]+ | 0 | LA | 2 | 5 |
| LPE(20:4) | 501.2873 | 2.74 | -- | C25H44NO7P | LC-MS | (-) | [M-H]- | 3 | LA | 2 | 7 |
| LPE(22:5) | 527.3011 | 3.31 | -- | C27H46NO7P | LC-MS | (-) | [M-H]- | 0 | LA | 2 | 5 |
| *Diacylglycerophosphoethanolamines* | | | | | | | | | | | |
| PE(34:1) | 701.5309 | 15.78 | -- | C39H76NO8P | LC-MS | (+) | [M+H]+ | 7 | DB | 3 | 8.7 |
| PE(32:0) | 691.5149 | 15.16 | -- | C37H74NO8P | LC-MS | (+) | [M+H]+ | 0 | LA | 2 | 3.6 |
| PE(34:1) | 717.5321 | 15.75 | -- | C39H76NO8P | LC-MS | (+) | [M+H]+ | 2 | LA | 2 | 0.3 |
| PE(34:2) | 715.5162 | 14.66 | -- | C39H74NO8P | LC-MS | (+) | [M+H]+ | 2 | LA | 2 | 1.7 |
| PE(36:2) | 743.5496 | 16.36 | -- | C41H78NO8P | LC-MS | (+) | [M+H]+ | 4 | LA | 2 | 2.2 |
| PE(36:3) | 741.5332 | 15.23 | -- | C41H76NO8P | LC-MS | (+) | [M+H]+ | 3 | LA | 2 | 1.2 |
| PE(36:4) | 739.5167 | 14.73 | -- | C41H74NO8P | LC-MS | (+) | [M+H]+ | 2 | LA | 2 | 1.2 |
| PE(37:4) | 753.5315 | 15.6 | -- | C42H76NO8P | LC-MS | (+) | [M+H]+ | 1 | LA | 2 | 4.7 |
| PE(38:3) | 769.5587 | 17.23 | -- | C43H80NO8P | LC-MS | (-) | [M-H]- | 5 | LA | 2 | 3 |
| PE(38:5) | 765.5327 | 15.26 | -- | C43H76NO8P | LC-MS | (+) | [M+H]+ | 3 | LA | 2 | 1.1 |
| PE(38:6) | 763.5172 | 14.56 | -- | C43H74NO8P | LC-MS | (+) | [M+H]+ | 3 | LA | 2 | 1.3 |
| PE(38:6) | 747.5221 | 15.51 | -- | C43H74NO8P | LC-MS | (+) | [M+H]+ | 3 | LA | 2 | 1.2 |
| PE(40:6) | 791.5473 | 16.52 | -- | C45H78NO8P | LC-MS | (-) | [M-H]- | 1 | LA | 2 | 3 |
| PE(40:7) | 789.5363 | 15.11 | -- | C45H76NO8P | LC-MS | (+) | [M+H]+ | 7 | LA | 2 | 1.2 |
| PE(40:8) | 787.5169 | 14.25 | -- | C45H74NO8P | LC-MS | (-) | [M-H]- | 3 | LA | 2 | 2 |
| PE(38:4) | 767.5521 | 16.44 | -- | C43H78NO8P | LC-MS | (+) | [M+H]+ | 7 | LA | 2 | 1.2 |
| Glycerophosphoglycerols | | | | | | | | | | | |
| *Monoacylglycerophosphoglycerols* | | | | | | | | | | | |
| LPG(16:0) | 484.2809 | 2.8 | -- | C22H45O9P | LC-MS | (-) | [M-H]- | 1 | LA | 2 | 4 |
| LPG(22:6) | 556.28 | 2.3 | -- | C28H45O9P | LC-MS | (-) | [M-H]- | 0 | LA | 2 | 3 |
| LPG(22:6) | 556.2803 | 2.46 | -- | C28H45O9P | LC-MS | (-) | [M-H]- | 0 | LA | 2 | 4 |
| *Diacylglycerophosphoethanolamines* | | | | | | | | | | | |
| PG(36:3) | 772.5223 | 13.54 | -- | C42H77O10P | LC-MS | (-) | [M-H]- | 4 | LA | 2 | 1 |
| PG(36:4) | 770.5102 | 14.42 | -- | C42H75O10P | LC-MS | (-) | [M-H]- | 0 | LA | 2 | 19 |
| PG(36:4) | 770.5094 | 12.37 | -- | C42H75O10P | LC-MS | (-) | [M-H]- | 0 | LA | 2 | 10 |
| PG(40:7) | 820.5229 | 13.44 | -- | C46H77O10P | LC-MS | (-) | [M-H]- | 3 | LA | 2 | 3 |
| PG(40:8) | 818.5083 | 12.22 | -- | C46H75O10P | LC-MS | (-) | [M-H]- | 2 | LA | 2 | 2 |
| PG(42:10) | 842.5093 | 12.24 | -- | C48H75O10P | LC-MS | (-) | [M-H]- | 1 | LA | 2 | 2 |
| PG(42:10) | 866.5083 | 12.13 | -- | C48H75O10P | LC-MS | (-) | [M-H]- | 2 | LA | 2 | 3 |
| Glycerophosphoserines | | | | | | | | | | | |
| *Monoacylglycerophosphoserines* | | | | | | | | | | | |
| LPS(16:0) | 497.2754 | 2.88 | -- | C22H44NO9P | LC-MS | (-) | [M-H]- | 0 | LA | 2 | 3 |
| LPS(20:3) | 547.2884 | 3.48 | -- | C26H46NO9P | LC-MS | (-) | [M-H]- | 5 | LA | 2 | 11 |
| LPS(20:4) | 545.2746 | 2.4 | -- | C26H44NO9P | LC-MS | (-) | [M-H]- | 2 | LA | 2 | 5 |
| *Diacylglycerophosphoserines* | | | | | | | | | | | |
| PS(36:4) | 783.5041 | 12.83 | -- | C42H74NO10P | LC-MS | (+) | [M+H]+ | 1 | LA | 2 | 11.5 |
| PS(38:4) | 811.5403 | 14.55 | -- | C44H78NO10P | LC-MS | (+) | [M+H]+ | 5 | LA | 2 | 4.3 |
| PS(38:6) | 807.5066 | 14.39 | -- | C44H74NO10P | LC-MS | (-) | [M-H]- | 2 | LA | 2 | 3 |
| PS(38:6) | 807.5022 | 15.02 | -- | C44H74NO10P | LC-MS | (-) | [M-H]- | 4 | LA | 2 | 1 |
| PS(40:6) | 835.5349 | 14.43 | -- | C46H78NO10P | LC-MS | (+) | [M+H]+ | 2 | LA | 2 | 4.9 |
| Glycerophosphoinositols | | | | | | | | | | | |
| *Diacylglycerophosphoinositols* | | | | | | | | | | | |
| PI(36:4) | 858.5261 | 13.97 | -- | C45H79O13P | LC-MS | (-) | [M-H]- | 0 | LA | 2 | 4 |
| PI(40:6) | 910.5568 | 15.62 | -- | C49H83O13P | LC-MS | (-) | [M-H]- | 0 | LA | 2 | 7 |
| Glycerolipids | | | | | | | | | | | |
| Diradylglycerols | | | | | | | | | | | |
| *Diacylglyerols* | | | | | | | | | | | |
| DG(36:3) | 654.462 | 23.24 | -- | C39H70O5 | LC-MS | (+) | [M+K]+ | 1 | LA | 2 | 7.6 |
| DG(40:5) | 708.5079 | 18.85 | -- | C43H74O5 | LC-MS | (+) | [M+K]+ | 2 | DB | 3 | 4.8 |
| DG(40:8) | 702.4621 | 23.14 | -- | C43H68O5 | LC-MS | (+) | [M+K]+ | 1 | DB | 3 | 11.5 |
| DG(40:8) | 702.4616 | 23.56 | -- | C43H68O5 | LC-MS | (+) | [M+K]+ | 1 | DB | 3 | 6.6 |
| DG(36:4) | 654.4625 | 17.04 | -- | C39H68O5 | LC-MS | (+) | [M+K]+ | 1 | LA | 2 | 5 |
| DG(38:6) | 678.4619 | 16.87 | -- | C41H68O5 | LC-MS | (+) | [M+K]+ | 1 | LA | 2 | 6 |
| DG(40:7) | 704.48 | 17.36 | -- | C43H70O5 | LC-MS | (+) | [M+K]+ | 3 | DB | 3 | 3 |
| Triradylglycerols | | | | | | | | | | | |
| *Triacylglyerols* | | | | | | | | | | | |
| TG(38:1) | 702.5212 | 20.71 | -- | C41H76O6 | LC-MS | (+) | [M+K]+ | 2 | DB | 3 | 2.6 |
| TG(40:1) | 730.5502 | 21.81 | -- | C43H80O6 | LC-MS | (+) | [M+K]+ | 2 | DB | 3 | 0.3 |
| TG(40:2) | 728.5362 | 21.12 | -- | C43H78O6 | LC-MS | (+) | [M+K]+ | 1 | DB | 3 | 1.9 |
| TG(42:1) | 758.5806 | 22.79 | -- | C45H84O6 | LC-MS | (+) | [M+K]+ | 3 | DB | 3 | 3.5 |
| TG(42:2) | 756.5684 | 22.18 | -- | C45H82O6 | LC-MS | (+) | [M+K]+ | 2 | DB | 3 | 2.2 |
| TG(44:2) | 784.5988 | 23.13 | -- | C47H86O6 | LC-MS | (+) | [M+K]+ | 1 | DB | 3 | 2.6 |
| TG(46:1) | 814.6443 | 24.55 | -- | C49H92O6 | LC-MS | (+) | [M+K]+ | 1 | LA | 2 | 4.9 |
| TG(46:2) | 812.6293 | 23.93 | -- | C49H90O6 | LC-MS | (+) | [M+K]+ | 0 | LA | 2 | 4.9 |
| TG(48:1) | 842.6776 | 25.6 | -- | C51H96O6 | LC-MS | (+) | [M+K]+ | 1 | LA | 2 | 3.8 |
| TG(48:2) | 840.6614 | 24.86 | -- | C51H94O6 | LC-MS | (+) | [M+K]+ | 1 | LA | 2 | 4.2 |
| TG(48:3) | 838.6442 | 24.22 | -- | C51H92O6 | LC-MS | (+) | [M+K]+ | 1 | LA | 2 | 4.8 |
| TG(49:1) | 856.6912 | 26.19 | -- | C52H98O6 | LC-MS | (+) | [M+K]+ | 1 | LA | 2 | 1.4 |
| TG(49:2) | 854.675 | 25.39 | -- | C52H96O6 | LC-MS | (+) | [M+K]+ | 2 | LA | 2 | 2.9 |
| TG(49:3) | 852.6593 | 24.69 | -- | C52H94O6 | LC-MS | (+) | [M+K]+ | 2 | DB | 3 | 2.5 |
| TG(50:1) | 870.7101 | 26.86 | -- | C53H100O6 | LC-MS | (+) | [M+K]+ | 3 | LA | 2 | 3.1 |
| TG(50:2) | 868.6936 | 25.97 | -- | C53H98O6 | LC-MS | (+) | [M+K]+ | 2 | LA | 2 | 3.1 |
| TG(50:3) | 866.6777 | 25.2 | -- | C53H96O6 | LC-MS | (+) | [M+K]+ | 2 | LA | 2 | 2.8 |
| TG(50:4) | 848.6852 | 24.51 | -- | C53H94O6 | LC-MS | (+) | [M+Na]+ | 2 | LA | 2 | 4.9 |
| TG(51:1) | 884.7231 | 27.59 | -- | C54H102O6 | LC-MS | (+) | [M+K]+ | 4 | LA | 2 | 3.5 |
| TG(51:2) | 882.7071 | 26.62 | -- | C54H100O6 | LC-MS | (+) | [M+K]+ | 1 | LA | 2 | 1.7 |
| TG(51:3) | 880.6919 | 25.77 | -- | C54H98O6 | LC-MS | (+) | [M+K]+ | 2 | LA | 2 | 10.1 |
| TG(52:1) | 898.74 | 28.42 | -- | C55H104O6 | LC-MS | (+) | [M+K]+ | 2 | LA | 2 | 4.9 |
| TG(52:2) | 896.725 | 27.33 | -- | C55H102O6 | LC-MS | (+) | [M+K]+ | 1 | LA | 2 | 3 |
| TG(52:3) | 894.7093 | 26.37 | -- | C55H100O6 | LC-MS | (+) | [M+K]+ | 1 | LA | 2 | 2.4 |
| TG(52:4) | 892.6937 | 25.54 | -- | C55H98O6 | LC-MS | (+) | [M+K]+ | 2 | LA | 2 | 1.9 |
| TG(52:5) | 869.7466 | 24.82 | -- | C55H96O6 | LC-MS | (+) | [M+H4N]+ | 1 | LA | 2 | 5.2 |
| TG(53:2) | 910.7382 | 28.12 | -- | C56H104O6 | LC-MS | (+) | [M+K]+ | 5 | LA | 2 | 4.1 |
| TG(53:3) | 908.7225 | 27.07 | -- | C56H102O6 | LC-MS | (+) | [M+K]+ | 1 | LA | 2 | 2.4 |
| TG(53:4) | 906.7077 | 26.16 | -- | C56H100O6 | LC-MS | (+) | [M+K]+ | 0 | LA | 2 | 2.9 |
| TG(54:2) | 924.756 | 29 | -- | C57H106O6 | LC-MS | (+) | [M+K]+ | 2 | LA | 2 | 6.1 |
| TG(54:3) | 922.7408 | 27.84 | -- | C57H104O6 | LC-MS | (+) | [M+K]+ | 2 | LA | 2 | 3.2 |
| TG(54:4) | 920.7255 | 26.81 | -- | C57H102O6 | LC-MS | (+) | [M+K]+ | 0 | LA | 2 | 1.9 |
| TG(54:5) | 902.7331 | 25.92 | -- | C57H100O6 | LC-MS | (+) | [M+Na]+ | 1 | LA | 2 | 2.8 |
| TG(55:2) | 938.7682 | 29.98 | -- | C58H108O6 | LC-MS | (+) | [M+K]+ | 2 | DB | 3 | 7.8 |
| TG(55:3) | 936.7534 | 28.68 | -- | C58H106O6 | LC-MS | (+) | [M+K]+ | 5 | LA | 2 | 4.7 |
| TG(56:3) | 950.7699 | 29.58 | -- | C59H108O6 | LC-MS | (+) | [M+K]+ | 2 | LA | 2 | 5.8 |
| TG(56:4) | 927.823 | 28.3 | -- | C59H106O6 | LC-MS | (+) | [M+H4N]+ | 3 | LA | 2 | 6.6 |
| TG(56:6) | 923.7916 | 26.74 | -- | C59H102O6 | LC-MS | (+) | [M+H4N]+ | 3 | LA | 2 | 5.7 |
| Sphingolipids | | | | | | | | | | | |
| Ceramides | | | | | | | | | | | |
| *N-acylsphingosines (ceramides)* | | | | | | | | | | | |
| Cer(36:1;O2) | 609.533 | 15.22 | -- | C36H71NO3 | LC-MS | (-) | [M+CHO2]- | 1 | LA | 2 | 7 |
| Cer(40:1;O2) | 667.6112 | 19.39 | -- | C40H79NO3 | LC-MS | (-) | [M+CHO2]- | 1 | LA | 2 | 8 |
| Cer(41:1;O2) | 681.627 | 20.07 | -- | C41H81NO3 | LC-MS | (-) | [M+CHO2]- | 0 | LA | 2 | 4 |
| Cer(44:1;O2) | 715.6234 | 21.66 | -- | C44H87NO3 | LC-MS | (+) | [M+K]+ | 1 | DB | 3 | 2 |
| Cer(34:2;O2) | 535.4964 | 13.11 | -- | C34H65NO3 | LC-MS | (+) | [M+H]+ | 0 | LA | 2 | 1.1 |
| Cer(40:2;O2) | 619.5886 | 18.37 | -- | C40H77NO3 | LC-MS | (+) | [M+H]+ | 3 | LA | 2 | 1.3 |
| Cer(41:2;O2) | 679.6114 | 19.26 | -- | C41H79NO3 | LC-MS | (-) | [M+CHO2]- | 0 | LA | 2 | 8 |
| Cer(42:2;O2) | 647.6211 | 19.8 | -- | C42H81NO3 | LC-MS | (+) | [M+H]+ | 1 | LA | 2 | 0.5 |
| Cer(42:1;O2) | 645.6045 | 18.62 | -- | C42H83NO3 | LC-MS | (+) | [M+H]+ | 2 | LA | 2 | 1.1 |
| Neutral glycosphingolipids | | | | | | | | | | | |
| *Simple Glc series (Hexosylceramides)* | | | | | | | | | | | |
| HexCer(42:1;O2) | 865.645 | 19.12 | -- | C48H93NO8 | LC-MS | (+) | [M+K]+ | 5 | DB | 3 | 5.8 |
| HexCer(34:1;O2) | 721.5458 | 13.1 | -- | C40H77NO8 | LC-MS | (+) | [M+Na]+ | 1 | DB | 3 | 7.6 |
| HexCer(40:1;O2) | 805.6382 | 18.16 | -- | C46H89NO8 | LC-MS | (+) | [M+Na]+ | 3 | DB | 3 | 18.4 |
| HexCer(42:1;O2) | 849.6493 | 19.55 | -- | C48H93NO8 | LC-MS | (+) | [M+K]+ | 4 | DB | 3 | 4.8 |
| HexCer(42:2;O2) | 831.6554 | 18.36 | -- | C48H91NO8 | LC-MS | (+) | [M+Na]+ | 1 | DB | 3 | 5.6 |
| Phosphosphingolipids | | | | | | | | | | | |
| *Sphingomyelins* | | | | | | | | | | | |
| SM(40:2;O2) | 784.6475 | 17.49 | -- | C45H89N2O6P | LC-MS | (+)/(-) | [M+H]+ | 2 | LA | 2 | 2 |
| SM(41:1;O2) | 800.6776 | 19.14 | -- | C46H93N2O6P | LC-MS | (+)/(-) | [M+H]+ | 1 | LA | 2 | 1.8 |
| SM(34:2;O2) | 700.5528 | 12 | -- | C39H77N2O6P | LC-MS | (+)/(-) | [M+H]+ | 1 | LA | 2 | 1.8 |
| SM(38:1;O2) | 758.6351 | 16.81 | -- | C43H87N2O6P | LC-MS | (+)/(-) | [M+H]+ | 7 | LA | 2 | 4.5 |
| SM(38:2;O2) | 756.6131 | 15.77 | -- | C43H85N2O6P | LC-MS | (+)/(-) | [M+H]+ | 2 | LA | 2 | 7.8 |
| SM(40:1;O2) | 786.6621 | 18.39 | -- | C45H91N2O6P | LC-MS | (+)/(-) | [M+H]+ | 1 | LA | 2 | 2.6 |
| SM(42:1;O2) | 814.693 | 19.83 | -- | C47H95N2O6P | LC-MS | (+)/(-) | [M+H]+ | 0 | LA | 2 | 1.2 |
| SM(43:1;O2) | 828.7057 | 20.53 | -- | C48H97N2O6P | LC-MS | (+)/(-) | [M+H]+ | 3 | LA | 2 | 1.9 |
| Sterol Lipids | | | | | | | | | | | |
| Sterols | | | | | | | | | | | |
| *Cholesterol and derivatives* | | | | | | | | | | | |
| Cholesterol | 386.3548 | 27.52 | -- | C27H46O | GC-MS | -- | -- | -- | F | 2 | 8.3 |
| CE(18:1) | 688.5535 | 26.12 | -- | C45H78O2 | LC-MS | (+) | [M+K]+ | 4 | DB | 3 | 8.2 |
| CE(18:2) | 686.5385 | 25.17 | -- | C45H76O2 | LC-MS | (+) | [M+K]+ | 3 | DB | 3 | 5.7 |
| CE(20:3) | 712.5551 | 25.57 | -- | C47H78O2 | LC-MS | (+) | [M+K]+ | 1 | DB | 3 | 13.7 |
| CE(20:4) | 710.54 | 24.81 | -- | C47H76O2 | LC-MS | (+) | [M+K]+ | 0 | DB | 3 | 5.9 |
| CE(22:4) | 738.5689 | 25.97 | -- | C49H80O2 | LC-MS | (+) | [M+K]+ | 4 | DB | 3 | 12.3 |
| CE(22:6) | 734.5389 | 24.52 | -- | C49H76O2 | LC-MS | (+) | [M+K]+ | 2 | DB | 3 | 5.6 |

Compound names after annotation process (compound), monoisotopic mass (mass), retention time or migration time expressed in minutes (RT), relative migration time for CE-MS compounds based on the migration time of the internal standard (RMT), chemical formula, analytical platform used for the detection of the compound: LC-MS, GC-MS or CE-MS, ESI mode applied in the analysis (only for LC-MS and CE-MS): positive (+) or negative (-), adduct with which the compound is detected, mass error expressed in PPM (error), ID source (ID S): DB: Database, F: Fiehn Library, ISF: in-source fragmentation pattern, LA: Lipid Annotator (MS/MS), N: Nist Library. ID levels (ID L): 1 standard, 2 putative, 3 tentative, 4 molecular formula, 5 Unknown, based on [Schrimpe-Rutledge A. et al](https://pubs.acs.org/doi/10.1007/s13361-016-1469-y) classification (16). Coefficient of variation of the metabolite in QC samples (QCs CV). LPC(P-XX:Y): Plasmalogen lysophosphatidylcholine; PC(P-XX:Y): Plasmalogen phosphatidylcholine. All other coding, as referred in text.

**Table 2_Supp. Statistical values of annotated metabolites and lipids found as statistically significant by any comparison. In this table only the values for WTFAN *vs.* WTCT and CPT1FAN *vs.* WTFAN comparisons are represented.**

|  | **WTFAN vs WTCT** | | | | | | **CPT1AFAN vs WTFAN** | | | | | |
| --- | --- | --- | --- | --- | --- | --- | --- | --- | --- | --- | --- | --- |
| Compound | %Change | Log_2_FC | *p* | pBH | *p*(corr) | JK | %Change | Log_2_FC | *p* | pBH | *p*(corr) | JK |
| TCA cycle | | | | | | | | | | | | |
| Pyruvic acid | -59.7 | -1.3 | **0.009** | **0.028** | **-0.68** | **JK** | 75.6 | 0.8 | **0.063** | 0.197 | **0.54** | **JK** |
| Lactic acid | 15.5 | 0.2 | *0.214* | *0.336* | *0.27* | *NS* | 38.1 | 0.5 | **0.017** | 0.118 | **0.58** | **JK** |
| Citric acid | 425.2 | 2.4 | **0.009** | **0.028** | **0.86** | **JK** | 1.26 | 0 | *0.937* | *0.979* | *0.04* | *NS* |
| Fumaric acid | -3.5 | -0.1 | *0.582* | *0.727* | *-0.05* | *NS* | 40.3 | 0.5 | **0.006** | **0.063** | **0.72** | **JK** |
| Malic acid | 8.2 | 0.1 | *0.597* | *0.729* | *0.19* | *NS* | 39.3 | 0.5 | **0.022** | 0.139 | **0.72** | **JK** |
| Urea cycle & Citrulline -NO cycle | | | | | | | | | | | | |
| Argininosuccinic acid | -3.1 | 0 | *0.66* | *0.762* | *0.01* | *NS* | 27.1 | 0.3 | 0.093 | 0.296 | **0.69** | **JK** |
| Arginine | -23.5 | -0.4 | **0.002** | **0.012** | **0.83** | **JK** | 26.5 | 0.3 | **0.002** | **0.047** | **0.85** | **JK** |
| Urea | 160.7 | 1.4 | **0.002** | **0.01** | **0.95** | **JK** | -12.9 | -0.2 | *0.08* | *0.208* | *-0.38* | *NS* |
| Ornithine | -45 | -0.9 | *0.121* | *0.206* | **0.55** | **JK** | 53.3 | 0.6 | *0.288* | *0.522* | *0.57* | *NS* |
| Glycine | -25.2 | -0.4 | **0.004** | **0.022** | **0.81** | **JK** | 24.4 | 0.3 | **0.004** | **0.078** | **0.68** | **JK** |
| Guanidinoacetate | -72.1 | -1.8 | **0.002** | **0.012** | **0.87** | **JK** | 90.1 | 0.9 | *0.18* | *0.404* | *0.42* | *NS* |
| Folate cycle & Methionine cycle | | | | | | | | | | | | |
| Methionine | -12.4 | -0.2 | *0.013* | *0.049* | *0.35* | *NS* | 16.6 | 0.2 | *0.013* | *0.134* | *0.52* | *NS* |
| S-adenosyl- homocysteine (SAH) | -32.5 | -0.6 | **0.061** | **0.135** | **0.63** | **JK** | 3 | 0 | *0.558* | *0.727* | *0.04* | *NS* |
| *N,N,N*-trimethyl- glycine (Glycine-betaine) | -56.8 | -1.2 | **0.013** | **0.049** | **0.73** | **JK** | 56.6 | 0.6 | *0.221* | *0.463* | *0.38* | *NS* |
| Choline | -16.4 | -0.3 | *0.05* | *0.127* | *0.48* | *NS* | 7.8 | 0.1 | *0.372* | *0.591* | *0.45* | *NS* |
| Amino acids | | | | | | | | | | | | |
| Alanine | -29.8 | -0.5 | **0.002** | **0.012** | **0.96** | **JK** | 28.4 | 0.4 | **0.002** | **0.047** | **0.71** | **JK** |
| Proline | -30.5 | -0.5 | **0.002** | **0.012** | **0.84** | **JK** | 26.3 | 0.3 | **0.002** | **0.047** | **0.86** | **JK** |
| Valine | -32.3 | -0.6 | **0.002** | **0.012** | **0.83** | **JK** | 28.9 | 0.4 | **0.002** | **0.047** | **0.91** | **JK** |
| Isoleucine/Leucine | -11.2 | -0.2 | *0.426* | *0.523* | *0.46* | *NS* | 7.3 | 0.1 | *0.426* | *0.644* | *0.46* | *NS* |
| Histidine | -32.2 | -0.6 | **0.002** | **0.012** | **0.9** | **JK** | 27.2 | 0.3 | **0.009** | 0.11 | **0.82** | NS |
| Phenylalanine | -3 | 0 | *0.913* | *0.944* | *0.07* | *NS* | 4.7 | 0.1 | *0.658* | *0.808* | *0.12* | *NS* |
| Tyrosine | -21.1 | -0.3 | **0.002** | **0.012** | **0.79** | **JK** | 26.9 | 0.3 | **0.002** | **0.047** | **0.78** | NS |
| Asparagine | -13.5 | -0.2 | *0.069* | *0.144* | *0.51* | *NS* | 15.9 | 0.2 | *0.379* | *0.597* | *0.53* | *NS* |
| Lysine | -34.9 | -0.6 | **0.002** | **0.012** | **0.83** | **JK** | 30.8 | 0.4 | **0.019** | 0.156 | **0.68** | NS |
| Serine | -27.4 | -0.5 | **0.002** | **0.012** | **0.88** | **JK** | 24.4 | 0.3 | **0.002** | **0.047** | **0.82** | *NS* |
| Glutamine | -36.5 | -0.7 | **0.022** | **0.069** | **0.69** | **JK** | 33.4 | 0.4 | 0.165 | 0.404 | **0.54** | NS |
| Glutamic acid | 1.7 | 0 | *0.015* | *0.051* | *-0.23* | *NS* | 4.4 | 0.1 | *0.015* | *0.136* | *0* | *NS* |
| Aspartic acid | -28.8 | -0.5 | **0.002** | **0.012** | **0.93** | **JK** | 25.6 | 0.3 | **0.002** | **0.047** | **0.9** | **JK** |
| Modified Amino Acids (MAAs) | | | | | | | | | | | | |
| 5-hydroxy-lysine | -81.3 | -2.4 | **0.002** | **0.012** | **0.92** | **JK** | 183.2 | 1.5 | 0.065 | 0.238 | **0.6** | **JK** |
| N2-Acetyl-lysine | 35.7 | 0.4 | *0.169* | *0.255* | **-0.52** | **JK** | -31.9 | -0.6 | **0.002** | **0.047** | **-0.89** | **JK** |
| Polyamines | | | | | | | | | | | | |
| Putrescine | -17.4 | -0.3 | *0.667* | *0.762* | *0.157* | *NS* | -4.7 | -0.1 | *0.56* | *0.727* | *0.03* | *NS* |
| Spermidine | 51.7 | 0.6 | **0.039** | 0.105 | **-0.74** | **JK** | -17.3 | -0.3 | *0.216* | *0.463* | *-0.29* | *NS* |
| Spermine | -39.2 | -0.7 | **0.013** | **0.049** | **0.72** | **JK** | 27.5 | 0.4 | *0.223* | *0.463* | *0.29* | *NS* |
| *N1*-acetylspermidine | 626.1 | 2.9 | **0.0002** | **0.012** | **-0.9** | **JK** | -25.1 | -0.4 | *0.24* | *0.463* | *-0.16* | *NS* |
| Nucleosides, Nucleotides, and analogues | | | | | | | | | | | | |
| Adenosine | -38.8 | -0.7 | 0.056 | 0.134 | **0.64** | **JK** | 15.9 | 0.2 | *0.699* | *0.83* | *0.12* | *NS* |
| *N*-methyl-adenosine | 432.2 | 2.4 | **0.009** | **0.037** | **-0.82** | **JK** | -37.8 | -0.7 | *0.31* | *0.531* | *-0.15* | *NS* |
| Hypoxanthine | -36.6 | -0.7 | **0.002** | **0.01** | **-0.72** | **JK** | 28.8 | 0.4 | *0.221* | *0.394* | *0.5* | *NS* |
| Inosine | -59.3 | -1.3 | **0.002** | **0.01** | **-0.92** | **JK** | 33.3 | 0.4 | *0.063* | *0.197* | *0.56* | *NS* |
| Uric acid | 54.7 | 0.6 | *0.461* | *0.66* | *0.38* | *NS* | -13.2 | -0.2 | *0.563* | *0.7* | *-0.04* | *NS* |
| Fatty acyls | | | | | | | | | | | | |
| Carnitine and Acylcarnitines | | | | | | | | | | | | |
| Carnitine | -30.1 | -0.5 | **0.013** | **0.049** | **0.65** | **JK** | -2.02 | 0 | *0.781* | *0.886* | *0.01* | *NS* |
| Tetradecanoyl-carnitine (CAR 14:0) | -82.7 | -2.5 | **0.009** | **0.029** | **0.61** | **JK** | 63.2 | 0.7 | *0.699* | *0.877* | *0.3* | *NS* |
| oleoylcarnitine (CAR 18:1) | -70.4 | -1.8 | **0.002** | **0.013** | **0.92** | **JK** | 19.5 | 0.3 | *0.818* | *0.925* | *0.2* | *NS* |
| linoleoylcarnitine (CAR 18:2) | -70.2 | -1.8 | **0.002** | **0.013** | **0.88** | **JK** | 49.5 | 0.6 | *0.31* | *0.495* | *0.49* | *NS* |
| Arachidonoyl-carnitine (CAR 20:4) | -63.2 | -1.4 | **0.002** | **0.013** | **0.79** | **JK** | 16.9 | 0.2 | *0.31* | *0.495* | *0.35* | *NS* |
| Cervonyl carnitine (CAR 22:6) | -64.2 | -1.5 | **0.002** | **0.013** | **0.69** | **JK** | 51.5 | 0.6 | **0.015** | **0.057** | **0.78** | **JK** |
| Fatty acids and conjugates | | | | | | | | | | | | |
| Myristic acid (FA 14:0) | -40.8 | -0.8 | **0.022** | 0.061 | **-0.74** | **JK** | 32.4 | 0.4 | 0.054 | 0.196 | **0.61** | **JK** |
| Palmitic acid (FA 16:0) | -38.7 | -0.7 | **0.002** | **0.01** | **-0.81** | **JK** | 18.3 | 0.2 | *0.203* | *0.381* | *0.67* | *NS* |
| Stearic acid (FA 18:0) | -31.5 | -0.5 | **0.015** | **0.045** | -0.65 | NS | 19.9 | 0.3 | *0.29* | *0.481* | *0.42* | *NS* |
| Linoleic acid (FA 18:2) | -34.5 | -0.6 | **0.019** | 0.056 | **-0.68** | **JK** | 19.7 | 0.3 | *0.29* | *0.481* | *0.42* | *NS* |
| Docosapentaenoic acid (FA 22:5) (DPA) | -41.2 | -0.8 | **0.017** | 0.056 | **0.78** | **JK** | 31.7 | 0.4 | *0.126* | *0.336* | *0.59* | *NS* |
| Fatty esters | | | | | | | | | | | | |
| Methylpalmitate | -53.9 | -1.1 | **0.004** | **0.016** | **-0.83** | **JK** | 87.9 | 0.9 | **0.013** | 0.108 | **0.76** | **JK** |
| Methylarachidonate | -35 | -0.6 | **0.009** | **0.029** | **0.77** | **JK** | 30.7 | 0.4 | **0.026** | 0.08 | **0.65** | **JK** |
| Glycerophospholipids | | | | | | | | | | | | |
| Glycerophosphocholines | | | | | | | | | | | | |
| Monoacylglycerophosphocholines | | | | | | | | | | | | |
| LPC(P-16:0) | 89.9 | 0.9 | **0.041** | 0.087 | **-0.68** | **JK** | -11.2 | -0.2 | *0.31* | *0.495* | *-0.11* | *NS* |
| LPC(20:3) | -45.1 | -0.9 | **0.009** | **0.029** | **0.76** | **JK** | 95.8 | 1 | **0.009** | **0.043** | **0.79** | **JK** |
| LPC(20:4) | -21.8 | -0.4 | *0.31* | *0.431* | *0.44* | *NS* | 41.4 | 0.5 | **0.009** | **0.043** | **0.75** | **JK** |
| LPC(22:6) | -46.2 | -0.9 | **0.004** | **0.02** | **0.82** | **JK** | 76.3 | 0.8 | **0.015** | 0.057 | **0.73** | **JK** |
| LPC(22:6) | -51.4 | -1 | **0.004** | **0.02** | **0.81** | **JK** | 72.6 | 0.8 | **0.004** | **0.042** | **0.85** | **JK** |
| Diacylglycerophosphocholines | | | | | | | | | | | | |
| PC(P-38:3) | 48.4 | 0.6 | **0.004** | **0.02** | **-0.82** | **JK** | -28.5 | -0.5 | 0.065 | 0.15 | **-0.62** | **JK** |
| PC(34:3) | -36.5 | -0.7 | **0.002** | **0.013** | **0.75** | **JK** | 43.1 | 0.5 | **0.009** | **0.043** | **0.77** | **JK** |
| PC(P-36:1) | 65.6 | 0.7 | **0.041** | 0.087 | **-0.64** | NS | -51 | -1 | **0.009** | **0.043** | **-0.78** | **JK** |
| PC(38:2) | -31.9 | -0.6 | **0.004** | **0.02** | **0.78** | **JK** | 34.5 | 0.4 | **0.041** | 0.109 | **0.73** | **JK** |
| PC(38:3) | -50.3 | -1 | **0.002** | **0.013** | **0.83** | **JK** | 63.1 | 0.7 | **0.009** | **0.043** | **0.78** | **JK** |
| PC(38:5) | -30.2 | -0.5 | **0.002** | **0.013** | **0.79** | **JK** | 26.3 | 0.3 | **0.002** | **0.033** | **0.87** | **JK** |
| PC(38:6) | -42.6 | -0.8 | **0.002** | **0.013** | **0.75** | **JK** | 42.8 | 0.5 | **0.002** | **0.034** | **0.92** | **JK** |
| PC(38:7) | -36.2 | -0.6 | **0.009** | **0.029** | **0.68** | **JK** | 33.8 | 0.4 | 0.065 | 0.15 | **0.61** | **JK** |
| PC(39:4) | -37.3 | -0.7 | **0.009** | **0.029** | **0.7** | **JK** | 59.5 | 0.7 | **0.004** | **0.041** | **0.88** | **JK** |
| PC(39:6) | -68.5 | -1.7 | **0.002** | **0.013** | **0.9** | **JK** | 144.9 | 1.3 | **0.002** | **0.033** | **0.87** | **JK** |
| PC(40:5) | -29.7 | -0.5 | **0.026** | *0.06* | **0.72** | **JK** | 69.7 | 0.8 | **0.002** | **0.033** | **0.93** | **JK** |
| PC(40:5) | -67.8 | -1.6 | **0.002** | **0.013** | **0.92** | **JK** | 131.6 | 1.2 | **0.004** | **0.042** | **0.83** | **JK** |
| PC(40:6) | -50.5 | -1 | **0.002** | **0.013** | **0.91** | **JK** | 79.6 | 0.8 | **0.002** | **0.034** | **0.95** | **JK** |
| PC(40:7) | -36.8 | -0.7 | **0.026** | 0.06 | **0.69** | **JK** | 57.9 | 0.7 | **0.009** | **0.043** | **0.66** | **JK** |
| PC(40:7) | -44.8 | -0.9 | **0.002** | **0.013** | **0.9** | **JK** | 46 | 0.5 | **0.015** | *0.057* | **0.73** | **JK** |
| PC(40:8) | -47 | -0.9 | **0.002** | **0.013** | **0.91** | **JK** | 61.6 | 0.7 | **0.002** | **0.033** | **0.93** | **JK** |
| PC(40:8) | -24.6 | -0.4 | *0.818* | *0.873* | *0.34* | *NS* | 36.5 | 0.4 | **0.004** | **0.041** | **0.82** | **JK** |
| PC(42:7) | -32.4 | -0.6 | **0.041** | 0.087 | **0.6** | **JK** | 64.7 | 0.7 | **0.004** | **0.041** | **0.79** | **JK** |
| PC(44:4) | 21.6 | 0.3 | *0.485* | *0.591* | *-0.26* | *NS* | 89.8 | 0.9 | **0.041** | 0.109 | **0.59** | NS |
| PC(38:6) | -51.7 | -1 | **0.004** | **0.02** | **0.83** | **JK** | 63.7 | 0.7 | **0.004** | **0.042** | **0.85** | **JK** |
| Glycerophosphoethanolamines | | | | | | | | | | | | |
| Monoacylglycerophosphoethanolamines | | | | | | | | | | | | |
| LPE(16:0) | -44.2 | -0.8 | **0.009** | **0.029** | **0.8** | **JK** | 48.6 | 0.6 | **0.009** | **0.043** | **0.74** | **JK** |
| LPE(18:0) | -40 | -0.7 | **0.004** | **0.02** | **0.84** | **JK** | 53.4 | 0.6 | **0.002** | **0.033** | **0.88** | **JK** |
| LPE(18:1) | -49.8 | -1 | **0.002** | **0.013** | **0.86** | **JK** | 43 | 0.5 | 0.065 | 0.15 | **0.57** | NS |
| LPE(20:4) | -41.3 | -0.8 | **0.004** | **0.021** | **0.76** | **JK** | 36.9 | 0.5 | 0.177 | 0.417 | **0.5** | **JK** |
| LPE(22:5) | -42.6 | -0.8 | 0.126 | 0.217 | **0.6** | **JK** | 11.2 | 0.2 | *0.247* | *0.504* | *0.21* | *NS* |
| Diacylglycerophosphoethanolamines | | | | | | | | | | | | |
| PE(34:1) | 2.7 | 0 | *0.589* | *0.686* | *-0.01* | *NS* | -21.4 | -0.3 | *0.937* | *0.979* | *-0.08* | *NS* |
| PE(32:0) | -41.4 | -0.8 | **0.009** | **0.029** | **0.8** | **JK** | 23.9 | 0.3 | 0.065 | 0.15 | **0.54** | **JK** |
| PE(34:1) | -36.7 | -0.7 | **0.002** | **0.013** | **0.87** | **JK** | 20.2 | 0.3 | **0.002** | **0.033** | **0.86** | **JK** |
| PE(34:2) | -50.7 | -1 | **0.002** | **0.013** | **0.89** | **JK** | 60.7 | 0.7 | **0.004** | **0.041** | **0.8** | **JK** |
| PE(36:2) | -50.5 | -1 | **0.002** | **0.013** | **0.92** | **JK** | 48.8 | 0.6 | **0.002** | **0.033** | **0.91** | **JK** |
| PE(36:3) | -62.2 | -1.4 | **0.002** | **0.013** | **0.87** | **JK** | 59.4 | 0.7 | **0.015** | 0.057 | **0.66** | **JK** |
| PE(36:4) | -62.4 | -1.4 | **0.002** | **0.013** | **0.92** | **JK** | 77 | 0.8 | **0.004** | **0.041** | **0.79** | **JK** |
| PE(37:4) | -66.4 | -1.6 | **0.002** | **0.013** | **0.91** | **JK** | 123.7 | 1.2 | **0.002** | **0.033** | **0.8** | **JK** |
| PE(38:3) | -41.9 | -0.8 | **0.004** | **0.021** | **0.83** | **JK** | 32.7 | 0.4 | **0.03** | 0.177 | **0.7** | **JK** |
| PE(38:5) | -68.1 | -1.6 | **0.002** | **0.013** | **0.94** | **JK** | 77.1 | 0.8 | **0.009** | **0.043** | **0.78** | **JK** |
| PE(38:6) | -55.2 | -1.2 | **0.002** | **0.013** | **0.76** | **JK** | 60.3 | 0.7 | **0.002** | **0.034** | **0.94** | **JK** |
| PE(38:6) | -41.5 | -0.8 | **0.009** | **0.029** | **0.8** | **JK** | 41.4 | 0.5 | **0.041** | 0.109 | **0.63** | **JK** |
| PE(40:6) | -39.1 | -0.7 | **0.004** | **0.021** | **0.94** | **JK** | 43.1 | 0.5 | **0.004** | 0.064 | **0.88** | **JK** |
| PE(40:7) | -58.3 | -1.3 | **0.002** | **0.013** | **0.89** | **JK** | 48.8 | 0.6 | **0.041** | 0.109 | **0.61** | NS |
| PE(40:8) | -43.5 | -0.8 | **0.004** | **0.021** | **0.94** | **JK** | 28.3 | 0.4 | **0.03** | 0.177 | **0.66** | **JK** |
| PE(38:4) | -43.7 | -0.8 | **0.002** | **0.013** | **0.88** | **JK** | 43.4 | 0.5 | **0.002** | 0.033 | **0.97** | **JK** |
| Glycerophosphoglycerols | | | | | | | | | | | | |
| Monoacylglycerophosphoglycerols | | | | | | | | | | | | |
| LPG(16:0) | -42.9 | -0.8 | **0.004** | **0.021** | **0.85** | **JK** | 36.3 | 0.4 | 0.126 | 0.336 | **0.56** | **JK** |
| LPG(22:6) | 121.2 | 1.1 | **0.004** | **0.021** | **-0.91** | **JK** | -27.3 | -0.5 | *0.247* | *0.504* | *-0.54* | *NS* |
| LPG(22:6) | 116.7 | 1.1 | **0.004** | **0.021** | **-0.84** | **JK** | -24.4 | -0.4 | *0.247* | *0.504* | *-0.44* | *NS* |
| Diacylglycerophosphoethanolamines | | | | | | | | | | | | |
| PG(36:3) | 87.6 | 0.9 | 0.662 | *0.733* | *-0.26* | *NS* | -79.1 | -2.3 | **0.017** | 0.123 | **-0.81** | **JK** |
| PG(36:4) | -44.7 | -0.9 | **0.009** | **0.033** | **0.81** | **JK** | 50.6 | 0.6 | **0.017** | 0.123 | **0.73** | **JK** |
| PG(36:4) | 55.6 | 0.6 | 0.662 | *0.733* | *-0.21* | *NS* | -79.7 | -2.3 | **0.017** | 0.123 | **-0.78** | **JK** |
| PG(40:7) | 156.4 | 1.4 | **0.017** | 0.056 | **-0.75** | **JK** | -64.8 | -1.5 | **0.017** | 0.123 | **-0.86** | **JK** |
| PG(40:8) | 147.3 | 1.3 | 0.052 | 0.117 | **-0.69** | **JK** | -57.2 | -1.2 | **0.017** | 0.123 | **-0.81** | **JK** |
| PG(42:10) | 231.6 | 1.7 | **0.004** | **0.021** | **-0.92** | **JK** | -35.5 | -0.6 | 0.126 | 0.336 | **-0.57** | **JK** |
| PG(42:10) | 672.2 | 2.9 | **0.004** | **0.021** | **-0.91** | **JK** | -30.1 | -0.5 | *0.329* | *0.576* | *-0.36* | *NS* |
| Glycerophosphoserines | | | | | | | | | | | | |
| Monoacylglycerophosphoserines | | | | | | | | | | | | |
| LPS(16:0) | -39.6 | -0.7 | **0.009** | **0.033** | **0.8** | **JK** | 27.6 | 0.4 | *0.177* | *0.417* | *0.42* | *NS* |
| LPS(20:3) | -45.8 | -0.9 | **0.004** | **0.021** | **0.84** | **JK** | 26.6 | 0.3 | *0.177* | *0.417* | *0.44* | *NS* |
| LPS(20:4) | -40.4 | -0.7 | **0.009** | **0.033** | **0.76** | **JK** | 25.5 | 0.3 | *0.247* | *0.504* | *0.38* | *NS* |
| Diacylglycerophosphoserines | | | | | | | | | | | | |
| PS(36:4) | -62.1 | -1.4 | **0.002** | **0.013** | **0.86** | **JK** | 44.6 | 0.5 | 0.132 | 0.254 | **0.52** | **JK** |
| PS(38:4) | -38.4 | -0.7 | **0.002** | **0.013** | **0.81** | **JK** | 23.5 | 0.3 | **0.004** | **0.041** | **0.81** | **JK** |
| PS(38:6) | -63.1 | -1.4 | **0.004** | **0.021** | **0.85** | **JK** | 23.2 | 0.3 | **0.03** | 0.177 | **0.69** | **JK** |
| PS(38:6) | -38.8 | -0.7 | **0.004** | **0.021** | **0.93** | **JK** | 68 | 0.7 | *0.126* | *0.336* | *0.44* | *JK* |
| PS(40:6) | -45.4 | -0.9 | **0.002** | **0.013** | **0.88** | **JK** | 45.5 | 0.5 | **0.004** | **0.041** | **0.83** | **JK** |
| Glycerophosphoinositols | | | | | | | | | | | | |
| Diacylglycerophosphoinositols | | | | | | | | | | | | |
| PI(36:4) | -38.7 | -0.7 | **0.004** | **0.021** | **0.88** | **JK** | 38.4 | 0.5 | **0.017** | 0.123 | **0.67** | **JK** |
| PI(40:6) | -37.9 | -0.7 | **0.03** | 0.081 | **0.7** | **JK** | 80.3 | 0.9 | **0.017** | 0.123 | **0.67** | **JK** |
| Glycerolipids | | | | | | | | | | | | |
| Diradylglycerols | | | | | | | | | | | | |
| Diacylglyerols | | | | | | | | | | | | |
| DG(36:3) | -66.8 | -1.6 | **0.002** | **0.013** | **0.87** | **JK** | 105.4 | 1 | **0.002** | **0.033** | **0.77** | **JK** |
| DG(40:5) | 9.1 | 0.1 | *0.485* | *0.591* | *-0.1* | *NS* | -10 | -0.2 | *0.485* | *0.684* | *-0.2* | *NS* |
| DG(40:8) | -61.4 | -1.4 | **0.002** | **0.013** | **0.73** | **JK** | 109.8 | 1.1 | **0.015** | **0.057** | **0.76** | **JK** |
| DG(40:8) | -56.7 | -1.2 | **0.015** | **0.041** | **0.62** | **JK** | 88.6 | 0.9 | **0.002** | **0.033** | **0.84** | **JK** |
| DG(36:4) | -50.2 | -1 | 0.093 | 0.17 | **0.63** | **JK** | 73.1 | 0.8 | **0.015** | **0.057** | **0.73** | **JK** |
| DG(38:6) | -42 | -0.8 | *0.093* | *0.17* | *0.54* | *NS* | 90.4 | 0.9 | **0.009** | **0.043** | **0.78** | **JK** |
| DG(40:7) | 102 | 1 | *0.589* | *0.686* | *-0.34* | *NS* | -36.8 | -0.7 | *0.818* | *0.925* | *-0.33* | *NS* |
| Triradylglycerols | | | | | | | | | | | | |
| Triacylglyerols | | | | | | | | | | | | |
| TG(38:1) | -95.8 | -4.6 | **0.004** | **0.02** | **0.7** | **JK** | 2492.2 | 4.7 | **0.009** | **0.043** | **0.63** | **JK** |
| TG(40:1) | -94.9 | -4.3 | **0.002** | **0.013** | **0.66** | **JK** | 1761.3 | 4.2 | **0.015** | 0.057 | **0.64** | **JK** |
| TG(40:2) | -96.5 | -4.8 | **0.004** | **0.02** | **0.68** | **JK** | 3004.6 | 5 | **0.015** | 0.057 | **0.67** | **JK** |
| TG(42:1) | -79.2 | -2.3 | **0.009** | **0.029** | **0.68** | **JK** | 303.8 | 2 | **0.026** | 0.08 | **0.66** | **JK** |
| TG(42:2) | -92.7 | -3.8 | **0.004** | **0.02** | **0.67** | **JK** | 1272.3 | 3.8 | **0.026** | 0.08 | **0.67** | **JK** |
| TG(44:2) | -80.8 | -2.4 | **0.004** | **0.02** | **0.69** | **JK** | 404.6 | 2.3 | **0.009** | **0.043** | **0.68** | **JK** |
| TG(46:1) | -75.4 | -2 | **0.009** | **0.029** | **0.67** | **JK** | 266.6 | 1.9 | **0.041** | 0.109 | **0.65** | **JK** |
| TG(46:2) | -77 | -2.1 | **0.009** | **0.029** | **0.67** | **JK** | 337.4 | 2.1 | **0.026** | 0.08 | **0.67** | **JK** |
| TG(48:1) | -69 | -1.7 | **0.009** | **0.029** | **0.67** | **JK** | 179.3 | 1.5 | **0.065** | 0.15 | **0.64** | **JK** |
| TG(48:2) | -77.2 | -2.1 | **0.009** | **0.029** | **0.67** | **JK** | 348.8 | 2.2 | **0.041** | 0.109 | **0.66** | **JK** |
| TG(48:3) | -77.1 | -2.1 | **0.015** | **0.041** | **0.65** | **JK** | 419.1 | 2.4 | **0.026** | 0.08 | **0.66** | **JK** |
| TG(49:1) | -45.3 | -0.9 | **0.004** | **0.02** | **0.82** | **JK** | 114.6 | 1.1 | **0.009** | **0.043** | **0.75** | **JK** |
| TG(49:2) | -52.2 | -1.1 | **0.009** | **0.029** | **0.77** | **JK** | 171.7 | 1.4 | **0.009** | **0.043** | **0.7** | **JK** |
| TG(49:3) | -57.8 | -1.2 | **0.009** | **0.029** | **0.71** | **JK** | 221.6 | 1.7 | **0.009** | **0.043** | **0.68** | **JK** |
| TG(50:1) | -58.6 | -1.3 | **0.009** | **0.029** | **0.71** | **JK** | 115.5 | 1.1 | **0.026** | 0.08 | **0.65** | **JK** |
| TG(50:2) | -65.9 | -1.6 | **0.009** | **0.029** | **0.72** | **JK** | 192.2 | 1.5 | **0.026** | 0.08 | **0.68** | **JK** |
| TG(50:3) | -74 | -1.9 | **0.009** | **0.029** | **0.69** | **JK** | 350.5 | 2.2 | **0.026** | 0.08 | **0.68** | **JK** |
| TG(50:4) | -71.8 | -1.8 | **0.015** | **0.041** | **0.63** | **JK** | 387.7 | 2.3 | **0.041** | 0.109 | **0.65** | **JK** |
| TG(51:1) | -54.2 | -1.1 | **0.004** | **0.02** | **0.75** | **JK** | 170.7 | 1.4 | **0.009** | **0.043** | **0.74** | **JK** |
| TG(51:2) | -63.1 | -1.4 | **0.009** | **0.029** | **0.75** | **JK** | 233.3 | 1.7 | **0.009** | **0.043** | **0.73** | **JK** |
| TG(51:3) | -67.7 | -1.6 | **0.004** | **0.02** | **0.7** | **JK** | 330.1 | 2.1 | **0.009** | **0.043** | **0.72** | **JK** |
| TG(52:1) | -67.6 | -1.6 | **0.009** | **0.029** | **0.66** | **JK** | 198.1 | 1.6 | **0.041** | 0.109 | **0.64** | **JK** |
| TG(52:2) | -59.7 | -1.3 | **0.009** | **0.029** | **0.72** | **JK** | 162.1 | 1.4 | **0.009** | **0.043** | **0.68** | **JK** |
| TG(52:3) | -60.6 | -1.3 | **0.009** | **0.029** | **0.73** | **JK** | 185.9 | 1.5 | **0.009** | **0.043** | **0.7** | **JK** |
| TG(52:4) | -63.9 | -1.5 | **0.015** | **0.041** | **0.68** | **JK** | 259.6 | 1.8 | **0.009** | **0.043** | **0.69** | **JK** |
| TG(52:5) | -72.8 | -1.9 | **0.041** | 0.087 | **0.58** | *NS* | 495.4 | 2.6 | **0.009** | **0.043** | **0.67** | **JK** |
| TG(53:2) | -67.1 | -1.6 | **0.004** | **0.02** | **0.69** | **JK** | 282.4 | 1.9 | **0.015** | 0.057 | **0.71** | **JK** |
| TG(53:3) | -68.1 | -1.6 | **0.004** | **0.02** | **0.7** | **JK** | 309.7 | 2 | **0.009** | **0.043** | **0.72** | **JK** |
| TG(53:4) | -67.3 | -1.6 | **0.009** | **0.029** | **0.66** | **JK** | 330.6 | 2.1 | **0.009** | **0.043** | **0.7** | **JK** |
| TG(54:2) | -69.4 | -1.7 | **0.009** | **0.029** | **0.67** | **JK** | 273.4 | 1.9 | **0.009** | **0.043** | **0.64** | **JK** |
| TG(54:3) | -61 | -1.4 | **0.009** | **0.029** | **0.71** | **JK** | 191.2 | 1.5 | **0.009** | **0.043** | **0.67** | **JK** |
| TG(54:4) | -54.1 | -1.1 | **0.009** | **0.029** | **0.68** | **JK** | 182.3 | 1.5 | **0.009** | **0.043** | **0.66** | **JK** |
| TG(54:5) | -62.8 | -1.4 | **0.026** | **0.06** | **0.58** | *NS* | 321.7 | 2.1 | **0.009** | **0.043** | **0.65** | **JK** |
| TG(55:2) | -69.9 | -1.7 | **0.004** | **0.02** | **0.6** | *NS* | 352.2 | 2.2 | **0.015** | 0.057 | **0.72** | **JK** |
| TG(55:3) | -71.4 | -1.8 | **0.009** | **0.029** | **0.64** | **JK** | 379.8 | 2.3 | **0.009** | **0.043** | **0.72** | **JK** |
| TG(56:3) | -65.3 | -1.5 | **0.015** | **0.041** | **0.64** | **JK** | 336.7 | 2.1 | **0.009** | **0.043** | **0.68** | **JK** |
| TG(56:4) | -64.4 | -1.5 | **0.026** | 0.06 | **0.57** | *NS* | 390.4 | 2.3 | **0.009** | **0.043** | **0.68** | **JK** |
| TG(56:6) | 4.7 | 0.1 | *0.589* | *0.686* | *-0.03* | *NS* | 129 | 1.2 | 0.065 | *0.15* | 0.41 | NS |
| Sphingolipids | | | | | | | | | | | | |
| Ceramides | | | | | | | | | | | | |
| N-acylsphingosines (ceramides) | | | | | | | | | | | | |
| Cer(36:1;O2) | -46.3 | -0.9 | **0.004** | **0.021** | **0.76** | **JK** | -4.5 | -0.1 | *0.537* | 0.727 | *-0.23* | *NS* |
| Cer(40:1;O2) | -35.2 | -0.6 | **0.009** | **0.034** | **0.84** | **JK** | 2.2 | 0 | *0.662* | *0.816* | *-0.01* | *NS* |
| Cer(41:1;O2) | -29.1 | -0.5 | 0.247 | 0.363 | 0.46 | **JK** | 4.6 | 0.1 | *0.429* | *0.645* | *0.03* | *NS* |
| Cer(44:1;O2) | 135.5 | 1.2 | *0.699* | *0.783* | *-0.45* | *NS* | 0.9 | 0 | *0.699* | *0.877* | *0.01* | *NS* |
| Cer(34:2;O2) | -34.2 | -0.6 | **0.015** | **0.041** | **0.68** | **JK** | 28.6 | 0.4 | *0.065* | *0.15* | **0.64** | *NS* |
| Cer(40:2;O2) | -43 | -0.8 | **0.002** | **0.013** | **0.88** | **JK** | 6.9 | 0.1 | *1* | *1* | *0.13* | *NS* |
| Cer(41:2;O2) | -49.2 | -1 | 0.126 | 0.217 | **0.53** | **JK** | 5.4 | 0.1 | *0.792* | *0.889* | *0.03* | *NS* |
| Cer(42:2;O2) | -31.8 | -0.6 | **0.002** | **0.013** | **0.8** | **JK** | 28.8 | 0.4 | **0.002** | **0.034** | **0.81** | **JK** |
| Cer(42:1;O2) | -31.3 | -0.5 | **0.002** | **0.013** | **0.86** | **JK** | 21.6 | 0.3 | **0.026** | 0.08 | **0.66** | *NS* |
| Neutral glycosphingolipids | | | | | | | | | | | | |
| Simple Glc series | | | | | | | | | | | | |
| HexCer(42:1;O2) | 62.3 | 0.7 | 0.132 | 0.225 | **-0.58** | *NS* | 17.2 | 0.2 | *0.485* | *0.684* | *0.23* | *NS* |
| HexCer(34:1;O2) | 107.2 | 1.1 | **0.009** | **0.029** | **-0.71** | **JK** | -9.1 | -0.1 | *0.485* | *0.684* | *-0.11* | *NS* |
| HexCer(40:1;O2) | 82.8 | 0.9 | **0.002** | **0.013** | **-0.79** | **JK** | -25 | -0.4 | **0.026** | 0.08 | **-0.53** | NS |
| HexCer(42:1;O2) | 50.7 | 0.6 | **0.015** | **0.041** | **-0.65** | **JK** | 8 | 0.1 | *0.699* | *0.877* | *0.14* | *NS* |
| HexCer(42:2;O2) | 64.6 | 0.7 | **0.015** | **0.041** | **-0.6** | **JK** | 12.5 | 0.2 | *0.937* | *0.979* | *0.12* | *NS* |
| Phosphosphingolipids | | | | | | | | | | | | |
| Sphingomyelins | | | | | | | | | | | | |
| SM(40:2;O2) | -40.5 | -0.7 | **0.009** | **0.029** | **0.77** | **JK** | -1.2 | 0 | *0.818* | *0.925* | *-0.1* | *NS* |
| SM(41:1;O2) | -38.7 | -0.7 | **0.004** | **0.02** | **0.83** | **JK** | 29.4 | 0.4 | **0.009** | **0.043** | **0.73** | **JK** |
| SM(34:2;O2) | -37.4 | -0.7 | **0.002** | **0.013** | **0.9** | **JK** | 28.6 | 0.4 | **0.015** | 0.057 | **0.75** | **JK** |
| SM(38:1;O2) | -41.2 | -0.8 | **0.009** | **0.029** | **0.78** | **JK** | 30.1 | 0.4 | 0.093 | 0.197 | **0.61** | NS |
| SM(38:2;O2) | -47.7 | -0.9 | **0.004** | **0.02** | **0.75** | **JK** | 0.6 | 0 | *0.589* | *0.794* | *0.13* | *NS* |
| SM(40:1;O2) | -40.5 | -0.7 | **0.004** | **0.02** | **0.74** | **JK** | 16.9 | 0.2 | **0.009** | **0.043** | **0.7** | **JK** |
| SM(42:1;O2) | -42.8 | -0.8 | 0.065 | 0.126 | **0.59** | NS | 37.2 | 0.5 | **0.004** | **0.042** | **0.72** | **JK** |
| SM(43:1;O2) | -32.8 | -0.6 | 0.065 | 0.126 | **0.62** | **JK** | 48.8 | 0.6 | **0.004** | **0.041** | **0.83** | **JK** |
| Sterol Lipids | | | | | | | | | | | | |
| Sterols | | | | | | | | | | | | |
| Cholesterol and derivatives | | | | | | | | | | | | |
| Cholesterol | -10.2 | -0.2 | *0.281* | *0.418* | *-0.28* | *NS* | 27.7 | 0.4 | *0.08* | *0.208* | *0.46* | *NS* |
| CE(18:1) | 457.3 | 2.5 | **0.004** | **0.02** | **-0.74** | **JK** | -47.4 | -0.9 | *0.093* | *0.197* | *-0.36* | *NS* |
| CE(18:2) | 287.8 | 2 | **0.002** | **0.013** | **-0.85** | **JK** | -37.3 | -0.7 | *0.065* | *0.15* | *-0.5* | *NS* |
| CE(20:3) | 388.8 | 2.3 | **0.004** | **0.02** | **-0.77** | **JK** | -56.6 | -1.2 | **0.026** | 0.08 | **-0.56** | NS |
| CE(20:4) | 396.1 | 2.3 | **0.004** | **0.02** | **-0.85** | **JK** | -47.5 | -0.9 | 0.065 | 0.15 | **-0.62** | NS |
| CE(22:4) | 2120.1 | 4.5 | **0.009** | **0.029** | **-0.57** | **JK** | -63.1 | -1.4 | *0.093* | *0.197* | *-0.37* | *NS* |
| CE(22:6) | 543 | 2.7 | **0.004** | **0.02** | **-0.71** | **JK** | -45.7 | -0.9 | *0.24* | *0.411* | *-0.39* | *NS* |

Compound names after annotation process (compound); %Change in the specific comparison obtained as Cases vs. Controls; logarithm in base two of the fold change obtained as Cases vs. Controls (Log_2_FC), consequently in the %Change and Log_2_FCthe negative sign (-) indicates that the metabolite is less abundant in the case group than in the control group, and the positive sign (+) means that the metabolites is more abundant in the case group than in control group; *p* value Mann Whitney U test (p), *p* value obtained with Benjamini-Hochberg correction test (pBH), values of correlation coefficient (p(corr)) and jackknife confidence interval (JK). The statistically significant values are highlighted in bold in each comparison, those metabolites that are not significant are in italics. Note that a distinction between univariate and multivariate statistics is made to highlight significance. LPC(P-XX:Y): Plasmalogen lysophosphatidylcholine; PC(P-XX:Y): Plasmalogen phosphatidylcholine. All other coding, as referred in text.

**Table 3_Supp. Statistical values of annotated metabolites and lipids found as statistically significant at any comparison. In this table only the values for CPT1AFAN vs. CPT1ACT and CPT1ACT vs. WTCT comparisons are represented.**

|  | **CPT1AFAN vs CPT1ACT** | | | | | | **CPT1ACT vs WTCT** | | | | | |
| --- | --- | --- | --- | --- | --- | --- | --- | --- | --- | --- | --- | --- |
| Compound | %Change | Log_2_FC | *p* | *p*BH | *p(*corr) | JK | %Change | Log_2_FC | *p* | *p*BH | *p*(corr) | JK |
| TCA cycle | | | | | | | | | | | | |
| Pyruvic acid | -5.5 | -0.1 | *0.563* | *0.7* | *-0.18* | *NS* | -25.1 | -0.4 | *0.461* | *0.764* | *-0.3* | *NS* |
| Lactic acid | 21.9 | 0.3 | *0.219* | *0.378* | *0.38* | *NS* | 30.8 | 0.4 | *0.154* | *0.469* | *0.37* | *NS* |
| Citric acid | 129.7 | 1.2 | **0.041** | 0.129 | **0.6** | NS | 131.6 | 1.2 | *0.784* | *0.918* | *0.18* | *NS* |
| Fumaric acid | 45.2 | 0.5 | **0.009** | **0.044** | **0.72** | **JK** | -6.8 | -0.1 | *0.667* | *0.849* | *-0.23* | *NS* |
| Malic acid | 55.4 | 0.6 | **0.035** | 0.118 | **0.63** | NS | -3 | 0 | *0.855* | *0.954* | *-0.12* | *NS* |
| Urea cycle & Citrulline -NO cycle | | | | | | | | | | | | |
| Argininosuccinic acid | 76.7 | 0.8 | **0.009** | **0.023** | **-0.78** | **JK** | -30.3 | -0.5 | *0.29* | *0.402* | *-0.6* | *NS* |
| Arginine | 20.6 | 0.3 | **0.006** | **0.023** | **-0.82** | **JK** | -19.7 | -0.3 | **0.009** | **0.027** | **-0.79** | **JK** |
| Urea | 123.3 | 1.2 | **0.002** | **0.017** | **0.92** | **JK** | 1.7 | 0 | *0.768* | *0.927* | *0.16* | *NS* |
| Ornithine | 9.2 | 0.1 | *0.816* | *0.875* | *-0.15* | *NS* | -22.8 | -0.4 | *0.699* | *0.77* | *-0.3* | *NS* |
| Glycine | 11.9 | 0.2 | *0.032* | *0.083* | *-0.48* | *NS* | -16.8 | -0.3 | *0.032* | *0.078* | *-0.65* | *JK* |
| Guanidinoacetate | -39.6 | -0.7 | 0.061 | 0.136 | **0.69** | **JK** | -12.2 | -0.2 | *0.351* | *0.462* | *-0.35* | *NS* |
| Folate cycle & Methionine cycle | | | | | | | | | | | | |
| Methionine | 29 | 0.4 | **0.002** | **0.013** | **-0.84** | **JK** | -20.9 | -0.3 | **0.015** | **0.04** | **-0.68** | **JK** |
| S-adenosyl- homocysteine (SAH) | -11.6 | -0.2 | *0.485* | *0.627* | *0.47* | *NS* | -21.4 | -0.3 | *0.065* | *0.135* | *-0.4* | *NS* |
| *N,N,N*-trimethyl- glycine (glycine-Betaine) | -32.6 | -0.6 | 0.061 | 0.136 | **0.63** | **JK** | 0.5 | 0 | *0.892* | *0.922* | *0.07* | *NS* |
| Choline | 17.6 | 0.2 | **0.009** | **0.023** | -0.53 | NS | -23.3 | -0.4 | *0.193* | *0.299* | *-0.62* | *JK* |
| Amino acids | | | | | | | | | | | | |
| Alanine | 2.5 | 0 | *0.734* | *0.813* | *-0.01* | *NS* | -12.1 | -0.2 | *0.011* | *0.033* | *-0.79* | *JK* |
| Proline | 17.3 | 0.2 | **0.002** | **0.013** | -0.78 | NS | -25.2 | -0.4 | **0.002** | **0.011** | **-0.81** | **JK** |
| Valine | 12.2 | 0.2 | *0.091* | *0.189* | *-0.66* | *NS* | -22.3 | -0.4 | *0.117* | *0.216* | *-0.71* | *JK* |
| Isoleucine/Leucine | 24.2 | 0.3 | **0.002** | **0.013** | **-0.78** | **JK** | -23.3 | -0.4 | *0.193* | *0.299* | *-0.72* | *JK* |
| Histidine | 14.6 | 0.2 | *0.372* | *0.539* | *-0.49* | *NS* | -24.8 | -0.4 | **0.002** | **0.011** | **-0.77** | **JK** |
| Phenylalanine | 44.3 | 0.5 | **0.009** | **0.023** | **-0.83** | **JK** | -29.7 | -0.5 | **0.002** | **0.011** | **-0.85** | **JK** |
| Tyrosine | 28.9 | 0.4 | **0.006** | **0.023** | **-0.78** | **JK** | -22.3 | -0.4 | **0.002** | **0.011** | **-0.84** | **JK** |
| Asparagine | 35.8 | 0.4 | **0.002** | **0.013** | **-0.81** | **JK** | -26.2 | -0.4 | **0.006** | **0.025** | **-0.84** | **JK** |
| Lysine | -1.4 | 0 | *0.909* | *0.937* | *-0.08* | *NS* | -13.7 | -0.2 | *0.117* | *0.216* | *-0.52* | *JK* |
| Serine | 13.3 | 0.2 | *0.461* | *0.615* | *-0.55* | *NS* | -20.3 | -0.3 | **0.002** | **0.011** | **-0.77** | **JK** |
| Glutamine | 3.1 | 0 | *0.784* | *0.864* | *-0.08* | *NS* | -17.8 | -0.3 | *0.165* | *0.281* | *-0.43* | *NS* |
| Glutamic | 17.4 | 0.2 | **0.002** | **0.013** | -0.58 | NS | -9.5 | -0.1 | *0.002* | *0.011* | *-0.75* | *JK* |
| Aspartic acid | 1.25 | 0 | *0.931* | *0.948* | *-0.16* | *NS* | -11.7 | -0.2 | *0.006* | *0.025* | *-0.69* | *JK* |
| Modified Amino Acids (MAAs) | | | | | | | | | | | | |
| 5-hydroxy-lysine | -24.3 | -0.4 | 0.132 | 0.25 | **0.52** | **JK** | -30.1 | -0.5 | *0.31* | *0.41* | *-0.34* | *JK* |
| N2-Acetyl-lysine | 119.9 | 1.1 | **0.004** | **0.016** | **-0.8** | **JK** | -58 | -1.3 | **0.009** | **0.027** | **-0.72** | **JK** |
| Polyamines | | | | | | | | | | | | |
| Putrescine | -34.5 | -0.6 | 0.058 | 0.136 | **0.71** | **JK** | 20.3 | 0.3 | *0.563* | *0.668* | *0.41* | *NS* |
| Spermidine | 53.1 | 0.6 | **0.004** | **0.016** | **-0.82** | **JK** | -18 | -0.3 | *0.039* | *0.091* | *-0.62* | *NS* |
| Spermine | 6.5 | 0.1 | *0.805* | *0.874* | *-0.02* | *NS* | -27.2 | -0.5 | *0.063* | *0.135* | *-0.55* | *JK* |
| *N1*-acetylspermidine | 679.6 | 3 | **0.002** | **0.013** | **-0.94** | **JK** | -30.2 | -0.5 | *0.18* | *0.283* | *-0.53* | *JK* |
| Nucleosides, Nucleotides, and analogues | | | | | | | | | | | | |
| Adenosine | -5.3 | -0.1 | *1* | *1* | *0.31* | *NS* | -25.1 | -0.4 | *0.056* | *0.127* | *-0.52* | *JK* |
| *N*-methyl-adenosine | 337.8 | 2.1 | **0.004** | **0.016** | **-0.87** | **JK** | -24.4 | -0.4 | *0.223* | *0.334* | *-0.5* | *JK* |
| Hypoxanthine | 10.4 | 0.1 | *0.206* | *0.367* | *0.09* | *NS* | -26 | -0.4 | **0.002** | **0.05** | **-0.64** | **JK** |
| Inosine | -41.25 | -0.8 | **0.009** | **0.044** | **-0.81** | **JK** | -7.6 | -0.1 | *0.567* | *0.796* | *-0.19* | *NS* |
| Uric acid | 87.1 | 0.9 | **0.024** | *0.089* | **0.73** | **JK** | -28.3 | -0.5 | *0.288* | *0.557* | *-0.32* | *NS* |
| Fatty acyls | | | | | | | | | | | | |
| Carnitine and Acylcarnitines | | | | | | | | | | | | |
| Carnitine | -2.9 | 0 | *0.45* | *0.612* | *0.11* | *NS* | -29.5 | -0.5 | 0.009 | 0.027 | -0.71 | JK |
| Tetradecanoyl-carnitine (CAR 14:0) | -35.4 | -0.6 | *0.167* | *0.563* | *0.36* | *NS* | -56.2 | -1.2 | *1* | *1* | *-0.39* | *NS* |
| oleoylcarnitine (CAR 18:1) | -63.5 | -1.5 | *0.095* | *0.46* | *0.73* | *NS* | -3.2 | 0 | *0.714* | *1* | *-0.1* | *NS* |
| linoleoylcarnitine (CAR 18:2) | -57.7 | -1.2 | *0.048* | *0.429* | *0.85* | *NS* | 4.6 | 0.1 | *0.905* | *1* | *0.03* | *NS* |
| Arachidonoyl-carnitine (CAR 20:4) | -46.8 | -0.9 | *0.167* | *0.563* | *0.63* | *NS* | -19.1 | -0.3 | *0.381* | *1* | *-0.28* | *NS* |
| Cervonyl carnitine (CAR 22:6) | -43.5 | -0.8 | *0.095* | *0.46* | *0.74* | *NS* | -4.1 | -0.1 | *1* | *1* | *-0.11* | *NS* |
| Fatty acids and conjugates | | | | | | | | | | | | |
| Myristic acid (FA 14:0) | 7.3 | 0.1 | *0.688* | *0.775* | *0.24* | *NS* | -27 | -0.5 | *0.206* | *0.497* | *-0.42* | *JK* |
| Palmitic acid (FA 16:0) | 0.32 | 0 | *0.942* | *0.958* | *0.05* | *NS* | -27.7 | -0.5 | *0.037* | *0.238* | *-0.54* | *NS* |
| Stearic acid (FA 18:0) | 11.4 | 0.2 | *0.457* | *0.588* | *0.24* | *NS* | -26 | -0.4 | *0.162* | *0.483* | *-0.41* | *NS* |
| Linoleic acid (FA 18:2) | 14.8 | 0.2 | *0.229* | *0.386* | *0.34* | *NS* | -31.7 | -0.6 | *0.024* | *0.173* | *-0.58* | *JK* |
| Docosapentaenoic acid (FA 22:5) (DPA) | -21 | -0.3 | *0.167* | *0.393* | *0.61* | *NS* | -1.92 | 0 | *0.714* | *1* | *-0.19* | *NS* |
| Fatty esters | | | | | | | | | | | | |
| Methylpalmitate | 6.5 | 0.1 | *0.667* | *0.768* | *0.19* | *NS* | -18.8 | -0.3 | *0.225* | *0.497* | *-0.32* | *NS* |
| Methylarachidonate | -5.2 | -0.1 | *0.905* | *0.969* | *0.34* | *NS* | -10.4 | -0.2 | *0.714* | *1* | *-0.35* | *NS* |
| Glycerophospholipids | | | | | | | | | | | | |
| Glycerophosphocholines | | | | | | | | | | | | |
| Monoacylglycerophosphocholines | | | | | | | | | | | | |
| LPC(P-16:0) | 88.2 | 0.9 | **0.024** | 0.396 | **-0.6** | **JK** | -10.3 | -0.2 | *0.381* | *1* | *-0.43* | *NS* |
| LPC(20:3) | -6 | -0.1 | *0.905* | *0.969* | *0.11* | *NS* | 14.3 | 0.2 | *0.548* | *1* | *0.27* | *NS* |
| LPC(20:4) | 18.2 | 0.2 | *0.167* | *0.563* | *-0.48* | *NS* | -6.4 | -0.1 | *1* | *1* | *-0.16* | *NS* |
| LPC(22:6) | -19.6 | -0.3 | *0.381* | *0.738* | *0.44* | *NS* | 17.8 | 0.2 | *0.548* | *1* | *0.36* | *NS* |
| LPC(22:6) | -16.2 | -0.3 | *0.262* | *0.643* | *0.59* | *NS* | 0.1 | 0 | *1* | *1* | *-0.06* | *NS* |
| Diacylglycerophosphocholines | | | | | | | | | | | | |
| PC(P-38:3) | 20.4 | 0.3 | *0.714* | *0.896* | *-0.27* | *NS* | -11.9 | -0.2 | *0.381* | *1* | *-0.45* | *NS* |
| PC(34:3) | -10.7 | -0.2 | *0.548* | *0.783* | *0.25* | *NS* | 1.8 | 0 | *1* | *1* | *0.03* | *NS* |
| PC(P-36:1) | 49.1 | 0.6 | *0.381* | *0.738* | *-0.39* | *NS* | -45.6 | -0.9 | **0.048** | 1 | **-0.53** | **JK** |
| PC(38:2) | -16.1 | -0.3 | *0.262* | *0.643* | *0.47* | *NS* | 9.2 | 0.1 | *0.381* | *1* | *0.26* | *NS* |
| PC(38:3) | -3.6 | -0.1 | *1* | *1* | *0.1* | *NS* | -15.9 | -0.2 | *0.381* | *1* | *-0.34* | *NS* |
| PC(38:5) | -8.2 | -0.1 | *0.095* | *0.46* | *0.67* | *JK* | -3.9 | -0.1 | *0.905* | *1* | *-0.24* | *NS* |
| PC(38:6) | -16.6 | -0.3 | **0.048** | 0.429 | **0.86** | **JK** | -1.7 | 0 | *0.905* | *1* | *-0.12* | *NS* |
| PC(38:7) | -5.1 | -0.1 | *0.714* | *0.896* | *0.25* | *NS* | -10.1 | -0.2 | *0.905* | *1* | *-0.33* | *NS* |
| PC(39:4) | 0.6 | 0 | *0.905* | *0.969* | *-0.01* | *NS* | -0.5 | 0 | *1* | *1* | *-0.03* | *NS* |
| PC(39:6) | -38.5 | -0.7 | *0.095* | *0.46* | **0.67** | *NS* | 25.2 | 0.3 | 0.548 | 1 | 0.39 | NS |
| PC(40:5) | -2.4 | 0 | *0.905* | *0.969* | *0.14* | *NS* | 22.3 | 0.3 | 0.167 | 1 | **0.56** | NS |
| PC(40:5) | -28.4 | -0.5 | *0.262* | *0.643* | **0.63** | *NS* | 4.1 | 0.1 | *1* | *1* | *0.14* | *NS* |
| PC(40:6) | -18.6 | -0.3 | *0.167* | *0.563* | **0.6** | *NS* | 9.3 | 0.1 | *0.548* | *1* | *0.22* | *NS* |
| PC(40:7) | -6.6 | -0.1 | *0.714* | *0.896* | *0.29* | *NS* | 6.9 | 0.1 | *0.714* | *1* | *0.03* | *NS* |
| PC(40:7) | -18.9 | -0.3 | *0.095* | *0.46* | **0.7** | JK | -0.7 | 0 | *1* | *1* | *-0.12* | *NS* |
| PC(40:8) | -19.4 | -0.3 | *0.095* | *0.46* | **0.72** | *NS* | 6.4 | 0.1 | *0.905* | *1* | *0.1* | *NS* |
| PC(40:8) | -14.4 | -0.2 | 0.048 | *0.429* | **0.67** | *NS* | 20.2 | 0.3 | *0.548* | *1* | *0.1* | *NS* |
| PC(42:7) | -23.7 | -0.4 | *0.262* | *0.643* | *0.43* | *NS* | 45.9 | 0.5 | *0.095* | *1* | **0.52** | *NS* |
| PC(44:4) | 119.6 | 1.1 | **0.024** | *0.396* | **-0.74** | JK | 5.1 | 0.1 | *0.548* | *1* | *0.02* | *NS* |
| PC(38:6) | -15 | -0.2 | *0.262* | *0.643* | **0.57** | *NS* | -6.9 | -0.1 | *0.714* | *1* | *-0.24* | *NS* |
| Glycerophosphoethanolamines | | | | | | | | | | | | |
| Monoacylglycerophosphoethanolamines | | | | | | | | | | | | |
| LPE(16:0) | -17.5 | -0.3 | 0.167 | 0.563 | 0.57 | NS | 0.6 | 0 | 0.905 | 1 | -0.05 | NS |
| LPE(18:0) | -4.3 | -0.1 | 0.548 | 0.783 | 0.24 | NS | -3.9 | -0.1 | 0.714 | 1 | -0.21 | NS |
| LPE(18:1) | -24.5 | -0.4 | 0.095 | 0.46 | 0.62 | NS | -4.8 | -0.1 | 0.905 | 1 | -0.23 | NS |
| LPE(20:4) | -23.7 | -0.4 | **0.024** | 0.183 | **0.66** | **JK** | 0.6 | 0 | 0.714 | 1 | 0.02 | NS |
| LPE(22:5) | -42.3 | -0.8 | **0.024** | 0.183 | **0.78** | **JK** | 10.4 | 0.1 | 0.714 | 1 | 0.19 | NS |
| Diacylglycerophosphoethanolamines | | | | | | | | | | | | |
| PE(34:1) | 20.3 | 0.3 | *0.262* | *0.643* | -0.28 | *NS* | -32.9 | -0.6 | **0.048** | 1 | **-0.68** | **JK** |
| PE(32:0) | -23.7 | -0.4 | **0.048** | 0.429 | **0.74** | **JK** | -4.9 | -0.1 | *0.905* | *1* | *-0.24* | *NS* |
| PE(34:1) | -20.6 | -0.3 | **0.048** | 0.429 | **0.85** | **JK** | -4 | -0.1 | *0.548* | *1* | *-0.24* | *NS* |
| PE(34:2) | -29.9 | -0.5 | **0.024** | 0.396 | **0.72** | **JK** | 13.1 | 0.2 | *0.381* | *1* | *0.35* | *NS* |
| PE(36:2) | -25.5 | -0.4 | **0.024** | 0.396 | **0.83** | **JK** | -1.1 | 0 | *0.714* | *1* | *-0.08* | *NS* |
| PE(36:3) | -39.5 | -0.7 | **0.024** | 0.396 | **0.82** | **JK** | -0.3 | 0 | *0.714* | *1* | *-0.03* | *NS* |
| PE(36:4) | -27.9 | -0.5 | **0.048** | 0.429 | **0.76** | **JK** | -7.6 | -0.1 | *0.381* | *1* | *-0.34* | *NS* |
| PE(37:4) | -25.5 | -0.4 | *0.262* | *0.643* | *0.5* | *NS* | 0.8 | 0 | *1* | *1* | *0* | *NS* |
| PE(38:3) | -22.2 | -0.4 | **0.048** | 0.223 | **0.78** | **JK** | -0.9 | 0 | *0.905* | *1* | *0.07* | *NS* |
| PE(38:5) | -37.9 | -0.7 | **0.048** | 0.429 | **0.85** | **JK** | -9.1 | -0.1 | *0.548* | *1* | *-0.35* | *NS* |
| PE(38:6) | -27.2 | -0.5 | **0.024** | 0.396 | **0.9** | **JK** | -1.3 | 0 | *0.714* | *1* | *-0.11* | *NS* |
| PE(38:6) | -16 | -0.3 | 0.095 | 0.46 | **0.49** | **JK** | -1.7 | 0 | *0.714* | *1* | *-0.08* | *NS* |
| PE(40:6) | -19.3 | -0.3 | **0.048** | 0.223 | **0.7** | **JK** | 8 | 0.1 | *0.548* | *1* | *0.44* | *NS* |
| PE(40:7) | -32.8 | -0.6 | **0.024** | 0.396 | **0.77** | **JK** | -7.6 | -0.1 | *0.714* | *1* | *-0.27* | *NS* |
| PE(40:8) | -24.3 | -0.4 | **0.024** | 0.183 | **0.74** | **JK** | -4.1 | -0.1 | *0.905* | *1* | *-0.26* | *NS* |
| PE(38:4) | -14.6 | -0.2 | **0.024** | 0.396 | **0.9** | **JK** | -5.5 | -0.1 | *0.548* | *1* | *-0.29* | *NS* |
| Glycerophosphoglycerols | | | | | | | | | | | | |
| Monoacylglycerophosphoglycerols | | | | | | | | | | | | |
| LPG(16:0) | -17.4 | -0.3 | *0.381* | *0.58* | *0.44* | *NS* | -5.7 | -0.1 | *0.548* | *1* | *-0.38* | *NS* |
| LPG(22:6) | 117.2 | 1.1 | 0.167 | 0.393 | **-0.53** | **JK** | -25.9 | -0.4 | 0.381 | 1 | **-0.67** | **JK** |
| LPG(22:6) | 118.6 | 1.1 | *0.381* | *0.58* | *-0.45* | *NS* | -25.1 | -0.4 | 0.381 | 1 | **-0.67** | **JK** |
| Diacylglycerophosphoethanolamines | | | | | | | | | | | | |
| PG(36:3) | -28.4 | -0.5 | 0.167 | 0.393 | **0.58** | NS | -45.4 | -0.9 | *1* | *1* | *-0.38* | *NS* |
| PG(36:4) | -16.5 | -0.3 | *0.381* | *0.58* | *0.47* | *NS* | -0.2 | 0 | *1* | *1* | *-0.01* | *NS* |
| PG(36:4) | -44.5 | -0.8 | 0.095 | 0.295 | **0.59** | NS | -43.2 | -0.8 | *1* | *1* | *-0.12* | *NS* |
| PG(40:7) | 22.2 | 0.3 | *0.714* | *0.832* | *-0.12* | *NS* | -26.1 | -0.4 | *0.714* | *1* | *-0.43* | *NS* |
| PG(40:8) | 31.7 | 0.4 | *0.548* | *0.714* | *-0.18* | *NS* | -19.7 | -0.3 | *1* | *1* | *-0.12* | *NS* |
| PG(42:10) | 135.5 | 1.2 | 0.167 | 0.393 | **-0.54** | **JK** | -9.2 | -0.1 | *1* | *1* | *-0.24* | *NS* |
| PG(42:10) | 544.9 | 2.7 | **0.048** | 0.223 | **-0.72** | **JK** | -16.3 | -0.3 | *0.381* | *1* | *-0.37* | *NS* |
| Glycerophosphoserines | | | | | | | | | | | | |
| Monoacylglycerophosphoserines | | | | | | | | | | | | |
| LPS(16:0) | -28.8 | -0.5 | *0.095* | 0.295 | **0.64** | **JK** | 8.2 | 0.1 | *0.548* | *1* | *0.49* | *NS* |
| LPS(20:3) | -30.1 | -0.5 | **0.024** | 0.183 | **0.69** | NS | -1.7 | 0 | *0.714* | *1* | *-0.21* | *NS* |
| LPS(20:4) | -22.8 | -0.4 | *0.095* | 0.295 | **0.59** | NS | -3 | 0 | *1* | *1* | *-0.17* | *NS* |
| Diacylglycerophosphoserines | | | | | | | | | | | | |
| PS(36:4) | -36.4 | -0.7 | **0.024** | 0.396 | **0.81** | **JK** | -13.7 | -0.2 | *0.548* | *1* | *-0.4* | *NS* |
| PS(38:4) | -13.4 | -0.2 | 0.167 | 0.563 | **0.76** | **JK** | -12.1 | -0.2 | *0.548* | *1* | *-0.39* | *NS* |
| PS(38:6) | -21.4 | -0.3 | **0.024** | 0.183 | **0.8** | **JK** | -4.1 | -0.1 | *0.905* | *1* | *-0.23* | *NS* |
| PS(38:6) | -42.4 | -0.8 | **0.024** | 0.183 | **0.63** | **JK** | 7.8 | 0.1 | *0.548* | *1* | *0.44* | *NS* |
| PS(40:6) | -26.2 | -0.4 | **0.024** | 0.396 | **0.79** | **JK** | 7.7 | 0.1 | *0.548* | *1* | *0.1* | *NS* |
| Glycerophosphoinositols | | | | | | | | | | | | |
| Diacylglycerophosphoinositols | | | | | | | | | | | | |
| PI(36:4) | -15.1 | -0.2 | **0.048** | 0.223 | **0.65** | **JK** | -0.1 | 0 | *1* | *1* | *-0.08* | *NS* |
| PI(40:6) | -5.9 | -0.1 | *0.548* | *0.714* | *0.12* | *NS* | 19 | 0.3 | *0.262* | *1* | *0.33* | *NS* |
| Glycerolipids | | | | | | | | | | | | |
| Diradylglycerols | | | | | | | | | | | | |
| Diacylglyerols | | | | | | | | | | | | |
| DG(36:3) | -35.8 | -0.6 | 0.095 | 0.46 | **0.7** | **JK** | 6.1 | 0.1 | *0.905* | *1* | *0.08* | *NS* |
| DG(40:5) | -35.1 | -0.6 | **0.024** | 0.396 | **0.85** | **JK** | 51.4 | 0.6 | **0.024** | *1* | **0.87** | **JK** |
| DG(40:8) | -12.1 | -0.2 | *0.548* | *0.783* | *0.35* | *NS* | -7.8 | -0.1 | *0.905* | *1* | *-0.2* | *NS* |
| DG(40:8) | -0.5 | 0 | *1* | *1* | *0.03* | *NS* | -17.9 | -0.3 | *1* | *1* | *-0.26* | *NS* |
| DG(36:4) | -31.7 | -0.6 | **0.048** | 0.429 | **0.71** | **JK** | 26.3 | 0.3 | *0.548* | *1* | *0.33* | *NS* |
| DG(38:6) | -34.3 | -0.6 | 0.167 | 0.563 | **0.67** | **JK** | 68 | 0.7 | 0.167 | 1 | **0.63** | **JK** |
| DG(40:7) | -19 | -0.3 | 0.262 | 0.643 | **0.58** | *NS* | 57.7 | 0.7 | **0.024** | 1 | **0.85** | **JK** |
| Triradylglycerols | | | | | | | | | | | | |
| Triacylglyerols | | | | | | | | | | | | |
| TG(38:1) | 74.1 | 0.8 | *1* | *1* | *-0.23* | *NS* | -37 | -0.7 | *0.381* | *1* | *-0.19* | *NS* |
| TG(40:1) | 75.9 | 0.8 | *1* | *1* | *-0.24* | *NS* | -45.8 | -0.9 | *0.262* | *1* | *-0.22* | *NS* |
| TG(40:2) | 46.5 | 0.6 | *1* | *1* | *-0.15* | *NS* | -25.1 | -0.4 | *0.905* | *1* | *-0.11* | *NS* |
| TG(42:1) | 48.7 | 0.6 | *1* | *1* | *-0.23* | *NS* | -43.6 | -0.8 | *0.262* | *1* | *-0.28* | *NS* |
| TG(42:2) | 42.7 | 0.5 | *1* | *1* | *-0.15* | *NS* | -30.2 | -0.5 | *0.548* | *1* | *-0.13* | *NS* |
| TG(44:2) | 27.3 | 0.3 | *1* | *1* | *-0.12* | *NS* | -24.1 | -0.4 | *0.548* | *1* | *-0.13* | *NS* |
| TG(46:1) | 62.4 | 0.7 | *0.905* | *0.969* | *-0.31* | *NS* | -44.5 | -0.8 | *0.167* | *1* | *-0.32* | *NS* |
| TG(46:2) | 36 | 0.4 | *0.905* | *0.969* | *-0.17* | *NS* | -26.1 | -0.4 | *0.381* | *1* | *-0.16* | *NS* |
| TG(48:1) | 69.5 | 0.8 | *0.381* | *0.738* | *-0.4* | *NS* | -49 | -1 | *0.167* | *1* | *-0.41* | *NS* |
| TG(48:2) | 65.9 | 0.7 | *0.905* | *0.969* | *-0.3* | *NS* | -38.4 | -0.7 | *0.548* | *1* | *-0.27* | *NS* |
| TG(48:3) | 41.8 | 0.5 | *0.905* | *0.969* | *-0.18* | *NS* | -16 | -0.3 | *0.905* | *1* | *-0.1* | *NS* |
| TG(49:1) | 64.9 | 0.7 | 0.381 | 0.738 | **-0.57** | NS | -28.8 | -0.5 | 0.095 | 1 | **-0.62** | **JK** |
| TG(49:2) | 77.7 | 0.8 | *0.548* | *0.783* | -0.46 | *NS* | -27 | -0.5 | 0.167 | 1 | -0.43 | **JK** |
| TG(49:3) | 48.1 | 0.6 | *0.905* | *0.969* | -0.27 | *NS* | -8.3 | -0.1 | *0.905* | *1* | *-0.14* | *NS* |
| TG(50:1) | 61.9 | 0.7 | 0.381 | 0.738 | **-0.5** | NS | -44.9 | -0.9 | *0.095* | *1* | *-0.51* | *NS* |
| TG(50:2) | 59.3 | 0.7 | *0.381* | *0.738* | -0.39 | *NS* | -37.5 | -0.7 | *0.262* | *1* | *-0.37* | *NS* |
| TG(50:3) | 61.3 | 0.7 | *0.548* | *0.783* | -0.3 | *NS* | -27.4 | -0.5 | *0.714* | *1* | *-0.21* | *NS* |
| TG(50:4) | 60.4 | 0.7 | *0.714* | *0.896* | -0.25 | *NS* | -14.3 | -0.2 | *0.905* | *1* | *-0.11* | *NS* |
| TG(51:1) | 88.9 | 0.9 | 0.381 | 0.738 | **-0.57** | NS | -34.3 | -0.6 | 0.095 | 1 | -0.5 | **JK** |
| TG(51:2) | 94.8 | 1 | *0.548* | *0.783* | -0.51 | *NS* | -36.9 | -0.7 | 0.381 | 1 | -0.45 | **JK** |
| TG(51:3) | 92.4 | 0.9 | *0.548* | *0.783* | -0.43 | *NS* | -27.8 | -0.5 | *0.714* | *1* | *-0.29* | *NS* |
| TG(52:1) | 148.6 | 1.3 | *0.381* | *0.738* | **-0.59** | **JK** | -61.1 | -1.4 | *0.167* | *1* | *-0.52* | *NS* |
| TG(52:2) | 65.8 | 0.7 | *0.548* | *0.783* | -0.48 | *NS* | -36.3 | -0.7 | *0.262* | *1* | *-0.42* | *NS* |
| TG(52:3) | 51.3 | 0.6 | *0.548* | *0.783* | -0.38 | *NS* | -25.5 | -0.4 | *0.381* | *1* | *-0.29* | *NS* |
| TG(52:4) | 57.1 | 0.7 | *0.714* | *0.896* | -0.33 | *NS* | -17.3 | -0.3 | *0.548* | *1* | *-0.18* | *NS* |
| TG(52:5) | 87.5 | 0.9 | *0.714* | *0.896* | -0.32 | *NS* | -13.8 | -0.2 | *0.714* | *1* | *-0.13* | *NS* |
| TG(53:2) | 133 | 1.2 | 0.548 | 0.783 | **-0.55** | NS | -46.1 | -0.9 | 0.262 | 1 | -0.46 | **JK** |
| TG(53:3) | 108.4 | 1.1 | 0.548 | 0.783 | **-0.5** | NS | -37.2 | -0.7 | *0.381* | *1* | *-0.38* | *NS* |
| TG(53:4) | 93.3 | 1 | *0.548* | *0.783* | -0.43 | *NS* | -27.2 | -0.5 | *0.381* | *1* | *-0.27* | *NS* |
| TG(54:2) | 178.5 | 1.5 | 0.548 | 0.783 | **-0.56** | **JK** | -59 | -1.3 | 0.262 | 1 | -0.5 | NS |
| TG(54:3) | 81.1 | 0.9 | *0.548* | *0.783* | -0.49 | *NS* | -37.3 | -0.7 | 0.381 | 1 | -0.41 | NS |
| TG(54:4) | 57.5 | 0.7 | *0.548* | *0.783* | -0.39 | *NS* | -17.6 | -0.3 | *0.714* | *1* | *-0.21* | *NS* |
| TG(54:5) | 97 | 1 | *0.548* | *0.783* | -0.41 | *NS* | -20.4 | -0.3 | *0.548* | *1* | *-0.21* | *NS* |
| TG(55:2) | 212.6 | 1.6 | 0.381 | 0.738 | **-0.59** | NS | -56.4 | -1.2 | *0.095* | *1* | *-0.45* | *NS* |
| TG(55:3) | 183.5 | 1.5 | 0.548 | 0.783 | **-0.57** | NS | -51.6 | -1 | 0.167 | 1 | -0.43 | NS |
| TG(56:3) | 204 | 1.6 | 0.548 | 0.783 | **-0.58** | **JK** | -50.2 | -1 | 0.167 | 1 | -0.45 | NS |
| TG(56:4) | 233.5 | 1.7 | 0.548 | 0.783 | **-0.57** | NS | -47.7 | -0.9 | 0.262 | 1 | -0.39 | NS |
| TG(56:6) | 486.5 | 2.6 | 0.024 | 0.396 | **-0.66** | **JK** | -59.1 | -1.3 | 0.167 | 1 | -0.42 | NS |
| Sphingolipids | | | | | | | | | | | | |
| Ceramides | | | | | | | | | | | | |
| N-acylsphingosines (ceramides) | | | | | | | | | | | | |
| Cer(36:1;O2) | -30.9 | -0.5 | *0.905* | *0.962* | **0.5** | *NS* | -25.8 | -0.4 | *0.381* | *1* | **-0.52** | *NS* |
| Cer(40:1;O2) | -29.8 | -0.5 | *0.095* | *0.295* | **0.76** | *NS* | -5.6 | -0.1 | *0.714* | *1* | *-0.42* | *NS* |
| Cer(41:1;O2) | -31.9 | -0.6 | *0.024* | *0.183* | **0.81** | **JK** | 8.9 | 0.1 | *0.548* | *1* | *0.19* | *NS* |
| Cer(44:1;O2) | -42.8 | -0.8 | *0.381* | *0.738* | **0.58** | *NS* | 315.5 | 2.1 | *0.048* | *1* | **0.65** | *NS* |
| Cer(34:2;O2) | 0.7 | 0 | *0.905* | *0.969* | *0.04* | *NS* | -15.9 | -0.2 | *0.714* | *1* | *-0.34* | *NS* |
| Cer(40:2;O2) | -14.3 | -0.2 | *0.381* | *0.738* | *0.42* | *NS* | -28.8 | -0.5 | *0.167* | *1* | **-0.71** | **JK** |
| Cer(41:2;O2) | -38.9 | -0.7 | *0.048* | *0.223* | **0.63** | **JK** | -12.4 | -0.2 | *1* | *1* | *-0.05* | *NS* |
| Cer(42:2;O2) | 4.3 | 0.1 | *0.548* | *0.783* | *-0.08* | *NS* | -15.8 | -0.2 | *0.167* | *1* | **-0.58** | **JK** |
| Cer(42:1;O2) | -4.1 | -0.1 | *0.548* | *0.783* | *0.31* | *NS* | -12.9 | -0.2 | *0.167* | *1* | **-0.6** | *NS* |
| Neutral glycosphingolipids | | | | | | | | | | | | |
| Simple Glc series | | | | | | | | | | | | |
| HexCer(42:1;O2) | 44.5 | 0.5 | **0.024** | 0.396 | **-0.69** | **JK** | 31.7 | 0.4 | **0.024** | 1 | **0.75** | **JK** |
| HexCer(34:1;O2) | 60.5 | 0.7 | 0.095 | 0.46 | **-0.51** | NS | 17.3 | 0.2 | *0.548* | *1* | *0.22* | *NS* |
| HexCer(40:1;O2) | 10.4 | 0.1 | *0.167* | *0.563* | *-0.24* | *NS* | 24.2 | 0.3 | **0.048** | 1 | 0.33 | NS |
| HexCer(42:1;O2) | 40.7 | 0.5 | *0.262* | *0.643* | *-0.41* | *NS* | 15.7 | 0.2 | *0.714* | *1* | *0.21* | *NS* |
| HexCer(42:2;O2) | 100.1 | 1 | 0.095 | 0.46 | -0.51 | NS | -7.5 | -0.1 | *0.714* | *1* | *-0.22* | *NS* |
| Phosphosphingolipids | | | | | | | | | | | | |
| Sphingomyelins | | | | | | | | | | | | |
| SM(40:2;O2) | -21.7 | -0.4 | 0.095 | 0.46 | **0.73** | NS | -25 | -0.4 | 0.167 | 1 | **-0.56** | **JK** |
| SM(41:1;O2) | -25.3 | -0.4 | **0.024** | 0.396 | **0.78** | **JK** | 6.1 | 0.1 | *0.905* | *1* | *0.16* | *NS* |
| SM(34:2;O2) | -5.8 | -0.1 | *0.905* | *0.969* | *0.38* | *NS* | -14.5 | -0.2 | 0.167 | 1 | **-0.71** | **JK** |
| SM(38:1;O2) | -20 | -0.3 | 0.095 | 0.46 | **0.67** | NS | -4.3 | -0.1 | *0.905* | *1* | *-0.18* | *NS* |
| SM(38:2;O2) | -28.1 | -0.5 | 0.167 | 0.563 | **0.65** | NS | -26.8 | -0.5 | *0.167* | *1* | *-0.41* | *NS* |
| SM(40:1;O2) | -24.4 | -0.4 | **0.024** | 0.396 | **0.92** | NS | -8 | -0.1 | *0.905* | *1* | *-0.24* | *NS* |
| SM(42:1;O2) | -19.7 | -0.3 | **0.048** | 0.429 | **0.8** | **JK** | -2.3 | 0 | *1* | *1* | *-0.09* | *NS* |
| SM(43:1;O2) | -22.1 | -0.4 | 0.167 | 0.563 | **0.63** | NS | 28.4 | 0.4 | *0.167* | *1* | *0.48* | *NS* |
| Sterol Lipids | | | | | | | | | | | | |
| Sterols | | | | | | | | | | | | |
| Cholesterol and derivatives | | | | | | | | | | | | |
| Cholesterol | 16 | 0.2 | *0.461* | *0.594* | *0.23* | *NS* | -1.1 | 0 | *0.665* | *0.849* | *-0.04* | *NS* |
| CE(18:1) | 88.3 | 0.9 | *1* | *1* | *-0.22* | *NS* | 55.6 | 0.6 | *0.714* | *1* | *0.33* | *NS* |
| CE(18:2) | -16.8 | -0.3 | *0.548* | *0.783* | *0.09* | *NS* | 192.2 | 1.5 | 0.548 | 1 | **0.64** | **JK** |
| CE(20:3) | 27.7 | 0.4 | *0.905* | *0.969* | *-0.17* | *NS* | 66 | 0.7 | *0.714* | *1* | *0.34* | *NS* |
| CE(20:4) | 22.2 | 0.3 | *0.905* | *0.969* | *-0.22* | *NS* | 113 | 1.1 | 0.548 | 1 | **0.53** | *NS* |
| CE(22:4) | 653.1 | 2.9 | *0.714* | *0.896* | *-0.28* | *NS* | 8.6 | 0.1 | *0.381* | *1* | *-0.03* | *NS* |
| CE(22:6) | 128.9 | 1.2 | *0.381* | *0.738* | *-0.43* | *NS* | 52.6 | 0.6 | *0.714* | *1* | *0.29* | *NS* |

Compound names after annotation process (compound); %Change in the specific comparison obtained as Cases vs. Controls; logarithm in base two of the fold change obtained as Cases vs. Controls (Log2FC), consequently in the %Change and Log2FCthe negative sign (-) indicates that the metabolite is less abundant in the case group than in the control group, and the positive sign (+) means that the metabolites is more abundant in the case group than in control group; p value Mann Whitney U test (p), p value obtained with Benjamini-Hochberg correction test (pBH), values of correlation coefficient (p(corr)) and jackknife confidence interval (JK). The statistically significant values are highlighted in bold in each comparison, those metabolites that are not significant are in italics. Note that a distinction between univariate and multivariate statistics is made to highlight significance. LPC(P-XX:Y): Plasmalogen lysophosphatidylcholine; PC(P-XX:Y): Plasmalogen phosphatidylcholine. All other coding, as referred in text.

|  | |  | **CPT1A FAN vs CPT1A CT** | | | **CPT1A CT** | | |
| --- | --- | --- | --- | --- | --- | --- | --- | --- |
|  |  |  |  |  |  | **vs WT CT** | | |
| **Metabolic Route** | **Gene Name** | | **Fold Change** | **q-value** | | **Fold Change** | | **q-value** |
| **TCA Cycle** | | ***Acly*** | 0.93 | | 0.8344 | 0.86 | 0.5115 | |
|  |  | ***Cs*** | 0.94 | | 0.8441 | 1.01 | 0.9654 | |
| **Urea cycle** | | ***Ass1*** | 0.96 | | 0.9306 | 2.15 | 0.0161 | |
| **Folate and methionine cycles** | | ***Bhmt*** | 0.97 | | 0.9218 | 0.62 | 0.0142 | |
|  |  | ***Gnmt*** | 0.36 | | 0.0211 | 0.86 | 0.5031 | |
| **MAAs** | | ***Plod3*** | 1.21 | | 0.6856 | 1.29 | 0.4938 | |
| **Polyamines** | | ***Odc1*** | 2.38 | | 0.0283 | 1.54 | 0.1110 | |
|  |  | ***Sms*** | 1.64 | | 0.0057 | 0.76 | 0.2236 | |
|  |  | ***Azin1*** | 1.22 | | 0.7143 | 1.76 | 0.3004 | |
| **Lipid Metabolism** | | ***Fasn*** | 0.75 | | 0.3033 | 1.07 | 0.6627 | |
|  |  | ***Acsm2*** | 0.60 | | 0.3106 | 1.59 | 0.3236 | |
|  |  | ***Dgat2*** | 0.90 | | 0.8055 | 1.65 | 0.1661 | |
|  |  | ***Srebf1*** | 1.87 | | 0.1833 | 1.07 | 0.7583 | |

**Table 4_Supp. TaqMan analysis results of gene expression from genes of the studied metabolic pathways and lipid metabolism in CPT1AFAN vs CPT1ACT, and CPT1CT vs. WTCT comparisons.**

Acly: ATP-citrate lyase, Cs: citrate synthase, Ass1: Argininosuccinate synthetase 1, Bhmt: Betaine-homocysteine S-methyltransferase, Gnmt: glycine N-methyltransferase, Plod3: Procollagen-Lysine,2-Oxoglutarate 5-Dioxygenase 3, Odc1: Ornithine decarboxylase, Sms: Spermine synthase, Azin1: Antizyme Inhibitor 1, Fasn: Fatty acid synthase, *Acsm2*: Acyl-CoA synthetase medium chain family member 2, *Dgat2*: diacylglycerol O-acyltransferase 2, *Srebf1*: sterol regulatory element-binding protein 1. mRNA levels of these genes were determined by qRT-PCR using TaqMan qPCR probes in kidneys from FAN model. Statistical significance in each comparison was determined using non-parametric two-tailed Mann-Whitney test and FDR adjustment. Relative mRNA expression was determined using 2^-ΔΔCt^ method and fold changes were normalized to values of the control group. Fold change higher than 1 indicates that the gene is upregulated in case group, foldchange equal to 1 means no differences in the expression of the gene, and foldchanges between 0 and 1 indicates a downregulation in case group in comparison with control group.
