# Supplementary figures and images for: Impact of renal tubular Cpt1a overexpression on the kidney metabolome in the folic acid-induced fibrosis mouse model
Source: Front Mol Biosci. 2023 Jun 12;10:1161036. doi: 10.3389/fmolb.2023.1161036 (PMC10291237; doi:10.3389/fmolb.2023.1161036)

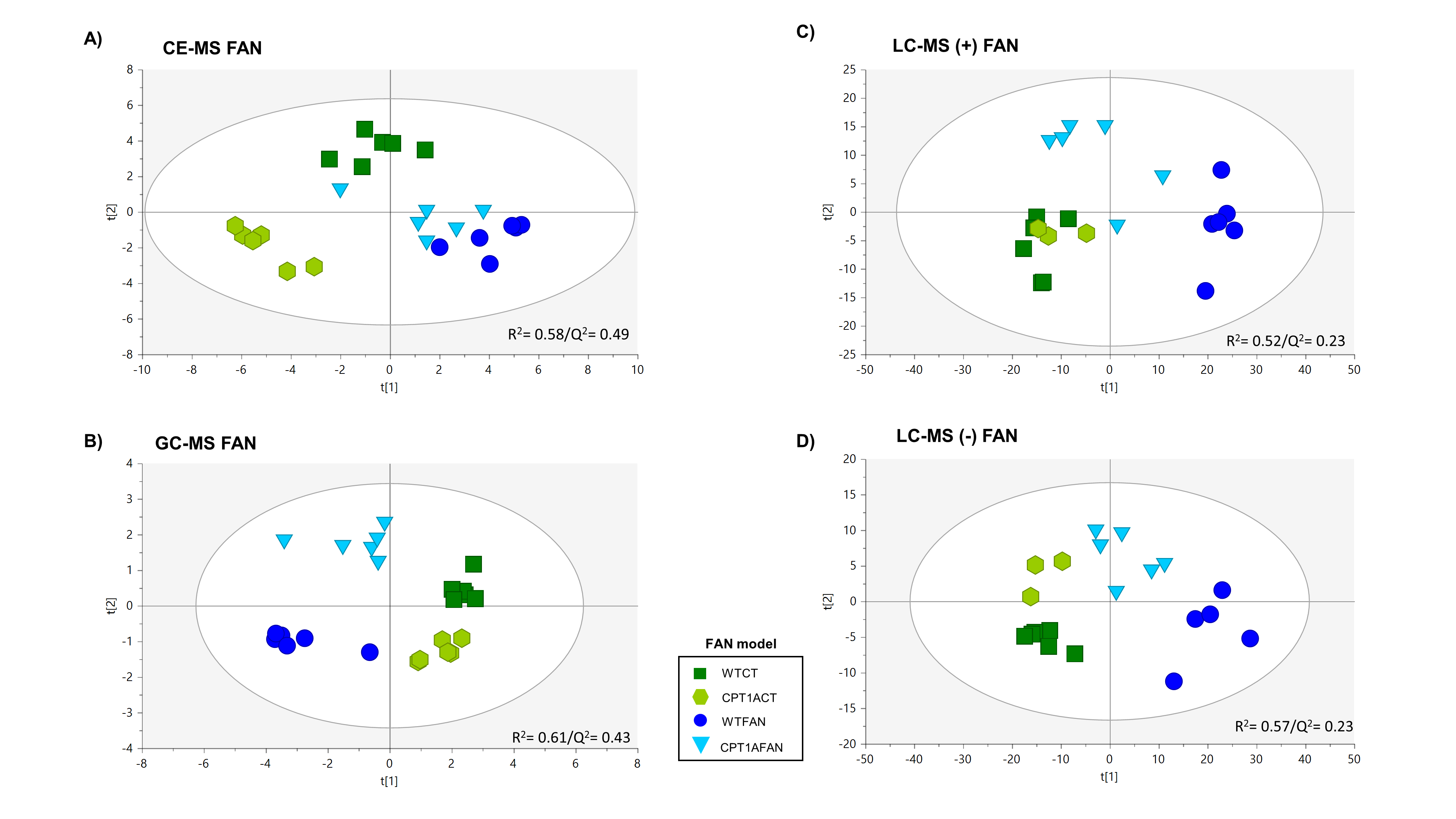

Supplement: Supplementary file 2 [file Image3.tif]

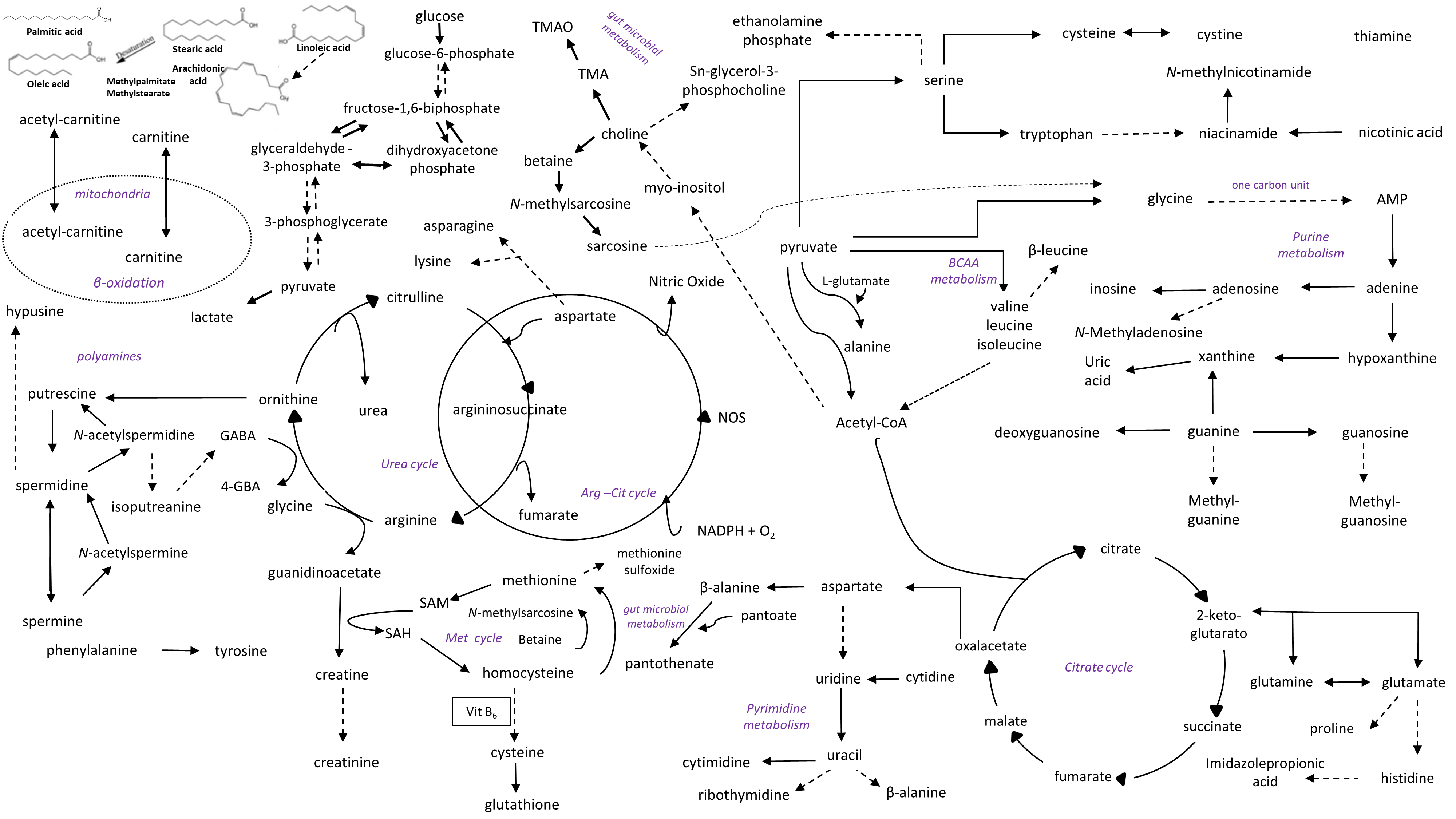

Supplement: Supplementary file 3 [file Image4.tif]

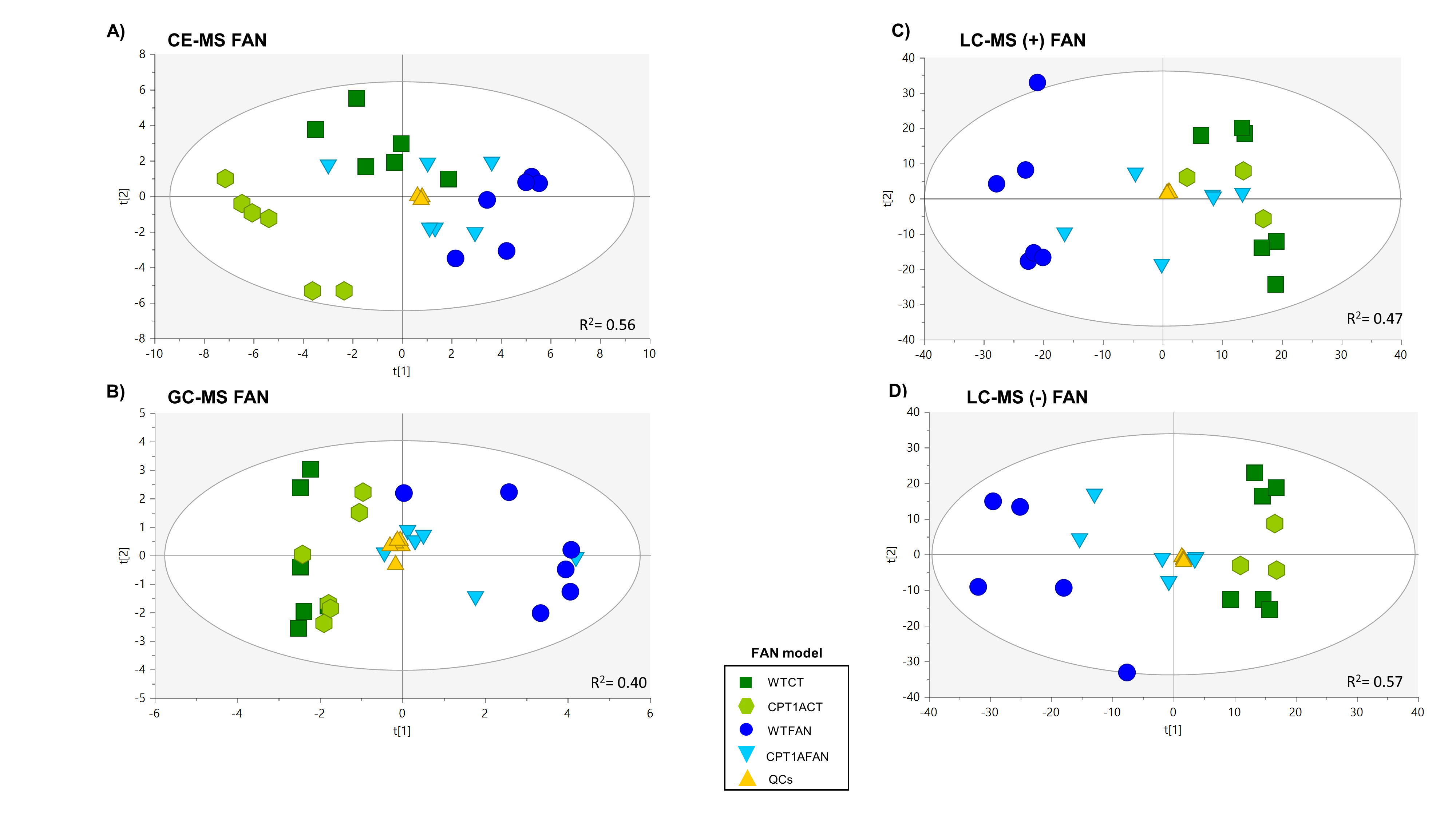

Supplement: Supplementary file 4 [file Image2.tif]

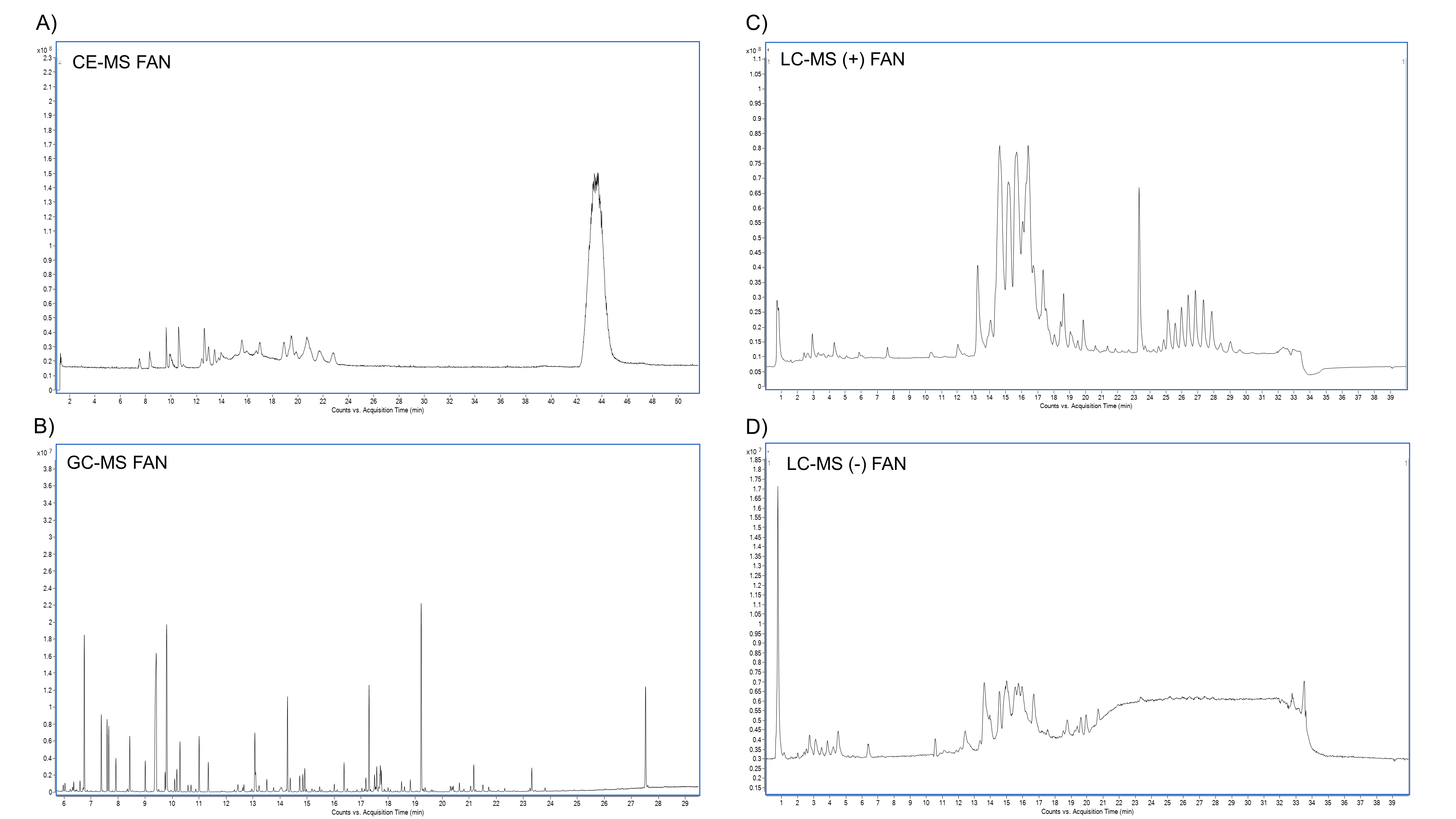

Supplement: Supplementary file 5 [file Image1.tif]
